# Supplementary material for: Switchable supramolecular helices for asymmetric stereodivergent catalysis
Source: Nat Commun. 2024 May 15;15:4116. doi: 10.1038/s41467-024-48412-z (PMC11096402; doi:10.1038/s41467-024-48412-z)
Supplement: Supplementary file 1 — Supplementary Information [file 41467_2024_48412_MOESM1_ESM.pdf]

Supplementary information

Switchable supramolecular helices for asymmetric stereodivergent catalysis

Ran Chen,[†] Ahmad Hammoud,[†] Paméla Aoun,[†] Mayte A. Martínez-Aguirre,[†] Nicolas Vanthuyne,[‡] Régina Maruchenko,[†] Patrick Brocorens,[§] Laurent Bouteiller,[†] and Matthieu Raynal[†,*]

† Sorbonne Université, CNRS, Institut Parisien de Chimie Moléculaire, Equipe Chimie des Polymères, 4 Place Jussieu, 75005 Paris (France) e-mail: matthieu.raynal@sorbonne-universite.fr.

‡ Aix Marseille Université, Centrale Marseille, CNRS, iSm2, UMR 7313, 13397 Marseille Cedex 20 (France)

§ Service de Chimie des Matériaux Nouveaux, Institut de Recherche sur les Matériaux, Université de Mons, Place du Parc, 20, B-7000, Mons (Belgium)

Supplementary Figs. 1-9 and Supplementary Tables 1-4 ____ 2

Control of the selectivity of the reaction by the catalyst (Supplementary Figs. 10-11) ____ 16

HPLC separation and characterization of the enantiopure stereoisomers of **APnol** (Supplementary Figs. 12-15, Supplementary Table 5) ____ 18

Determination of the absolute and relative configurations of **APnol** (Supplementary Figs. 16-19) 21

Determination of the ee, dr, ee1 and ee2 from the HPLC traces (Supplementary Fig. 20) ____ 25

Selected ^{1}H NMR spectra (Supplementary Figs. 21-24) ____ 26

Selected HPLC traces (Supplementary Figs. 25-40) ____ 28

Characterization of the catalytic products (Supplementary Figs. 41-46) ____ 36

Synthesis of 3-vinylacetophenone, **VPnone** (Supplementary Figs. 47-48) ____ 40

Catalytic procedures ____ 42

Preparation of the solution for CD analyses of the BTA pre-catalyst (CD data for Fig. 2) ____ 54

Supplementary references ____ 55

Supplementary Figs. 1-9 and Supplementary Tables 1-4

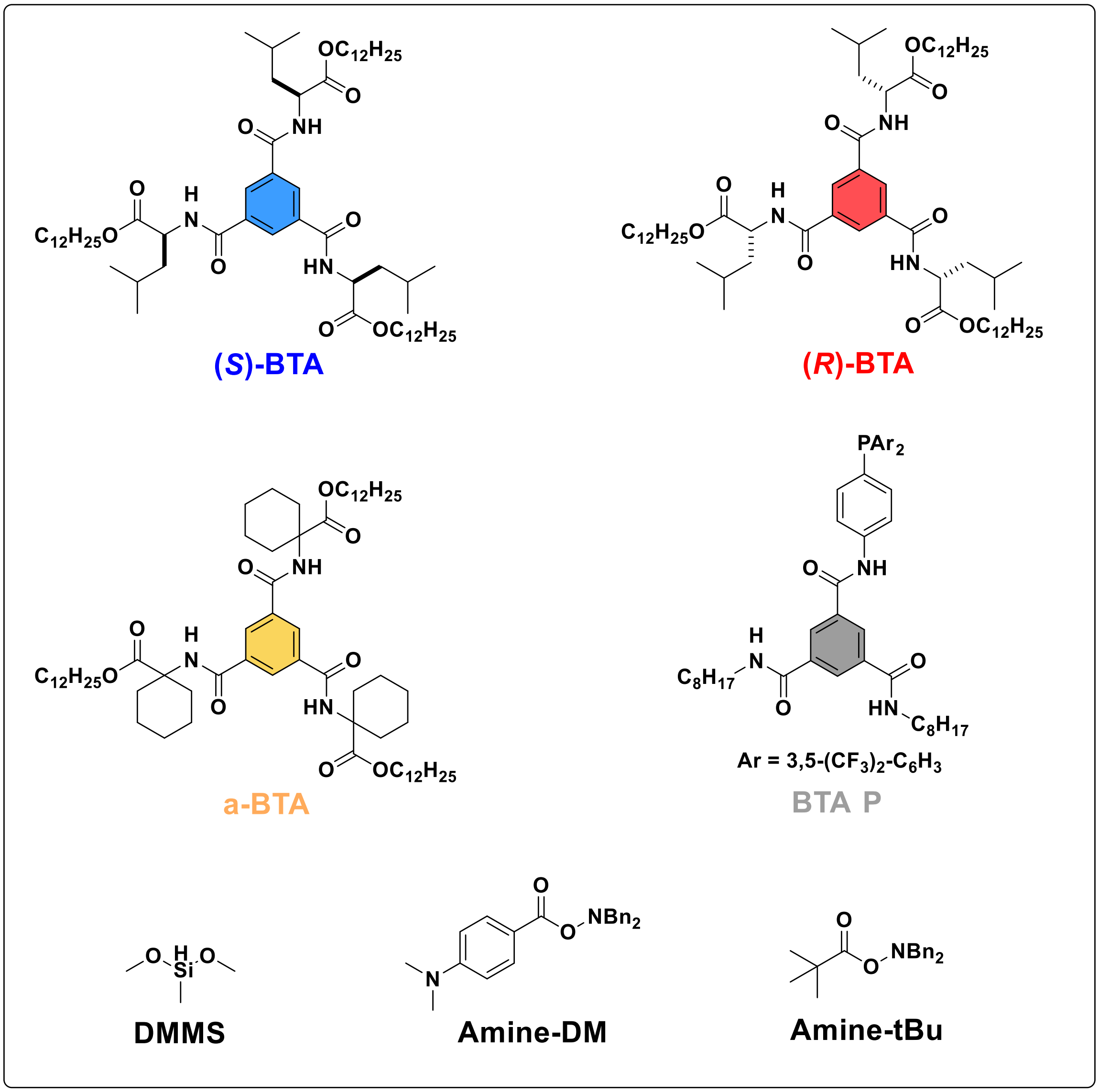

Supplementary Fig. 1. Chemical structures of BTA monomers and catalytic materials used in this study. Throughout this paper the fraction of enantiopure BTA monomers in the BTA helical catalysts is defined as follows: f_s= [**(*S*)-BTA**]/([**(*S*)-BTA**]+[**BTA P**]+[**a-BTA**]) when the only enantiopure monomer is **(*S*)-BTA**, f_s= [**(*R*)-BTA**]/([**(*R*)-BTA**]+[**BTA P**]+[**a-BTA**]) when the only enantiopure monomer is **(*R*)-BTA**, f_s= ([**(*S*)-BTA**]+[**(*R*)-BTA**])/([**(*S*)-BTA**]+[**(*R*)-BTA**]+[**BTA P**]+[**a-BTA**]) when both enantiopure monomers are present.

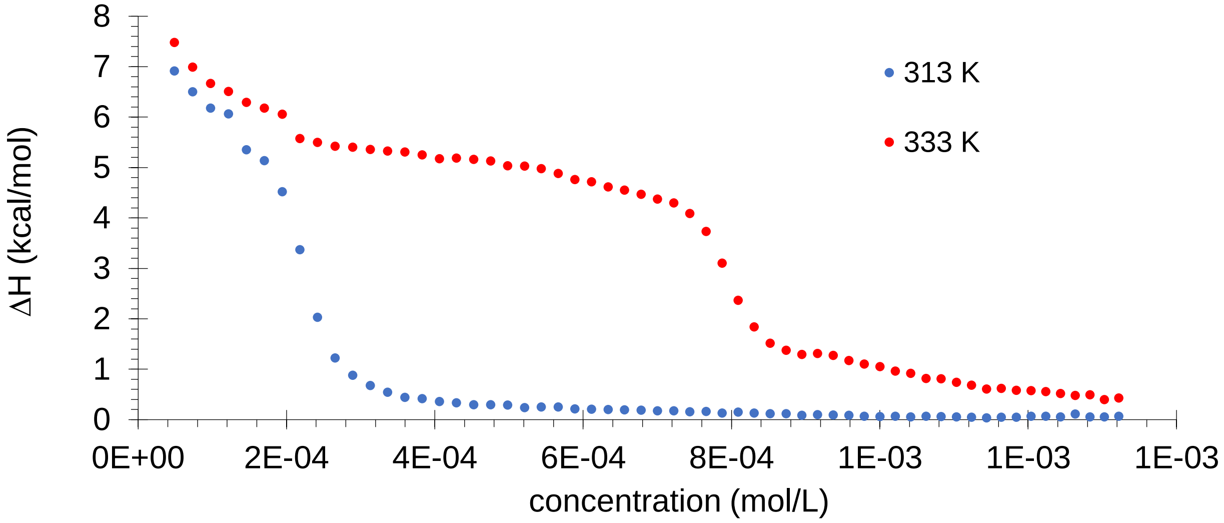

Supplementary Fig. 2. Stability of the supramolecular helical BTA catalyst in toluene at two different temperatures as determined by ITC. ITC enthalpograms are obtained for the mixture of BTA monomers in toluene injected into pure toluene, versus total ester BTA concentration in the cell at the given temperature. BTA monomer solution: **BTA P** (2.8 mM), **a-BTA** (2.8 mM), **(*S*)-BTA** (1.4 mM) corresponding to a total BTA concentration of 7.0 mM.

Comments on Supplementary Fig. 2: Critical concentrations (c*) are extracted from the ITC enthalpograms at the first point for which no more hydrogen bonds are disrupted, i.e. a value close to ΔH = 0 is reached. It yields the value of c*≈ 0.4 mM and 1.3 mM at 293 K and 313 K, respectively.

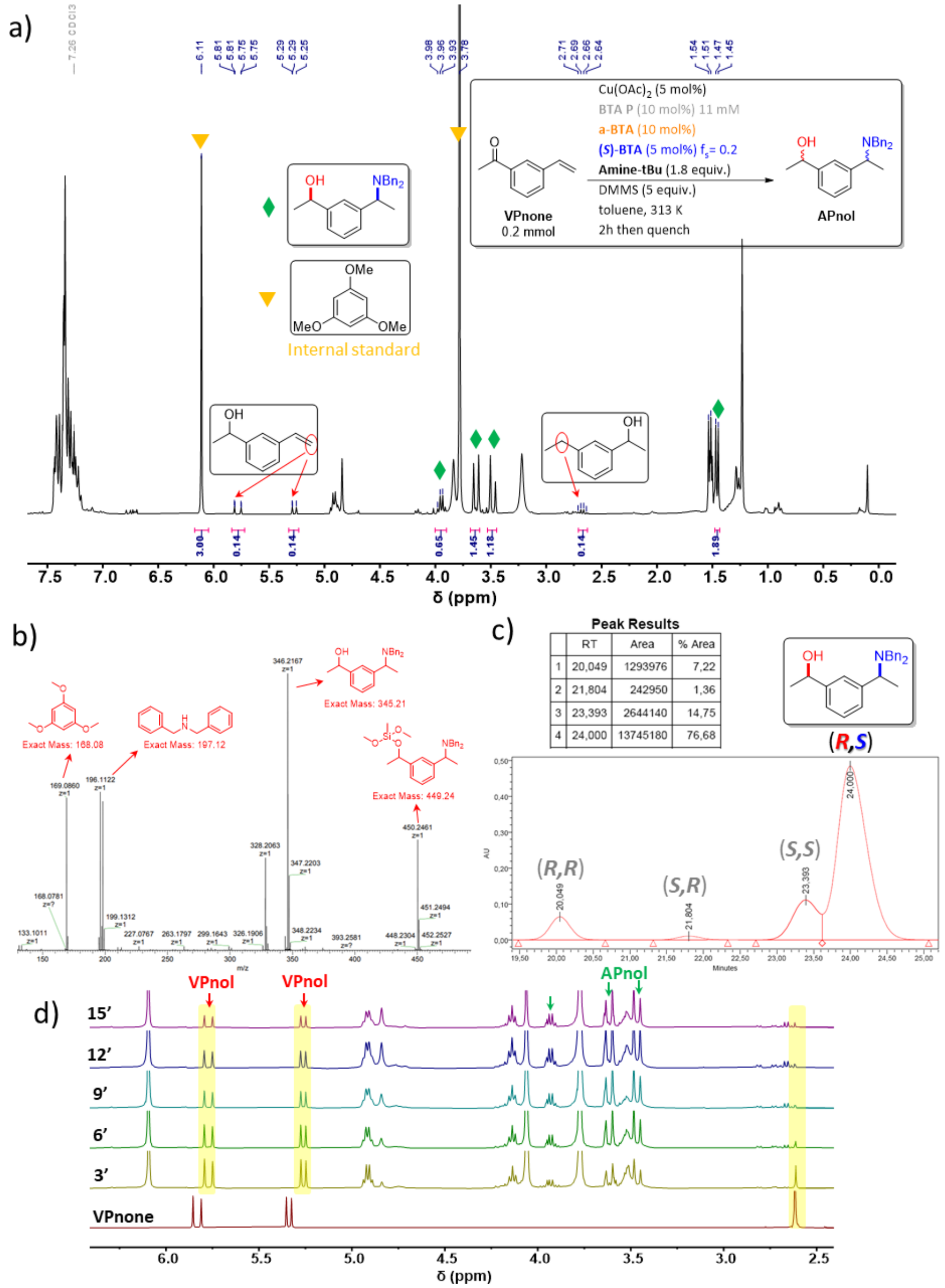

Supplementary Fig. 3. Hydrosilylation (HS)/hydroamination (HA) cascade transformation of **VPnone** with the BTA helical catalyst (concomitant reactions). ^{1}H NMR spectrum (a), MS spectrum (b) and chiral HPLC trace (c) of the crude sample obtained under standard conditions of Supplementary Table 1. **APnol** is obtained in *ca.* 66% yield and (***R*,*S***)**-APnol** is the main stereoisomer (97% ee, dr of 3.6:1). The crude NMR spectrum is shown together with selected NMR signals for **APnol** (green diamonds), **VPnol** (14%) and **EPnol** (*ca.* 10%). d) ^{1}H NMR monitoring of the reaction under the same conditions indicating that the ketone group (disappearance of the singlet at 2.6 ppm corresponding to the methyl group of **VPnone**) is consumed more rapidly than the vinyl moiety (signals at 5.4 and 5.8 ppm). The non-labelled signals at δ= 4.9 ppm and 1.5 ppm correspond to the methine and methyl groups group (resp.) next to OH and are thus common to **APnol**, **VPnol** and **EPnol**.

Supplementary Table 1. Screened conditions for the HS/HA cascade transformation of VPnone with BTA helical catalysts (concomitant reactions). Entry 8 corresponds to our optimized conditions for the concomitant process (Table 1, entry 1).

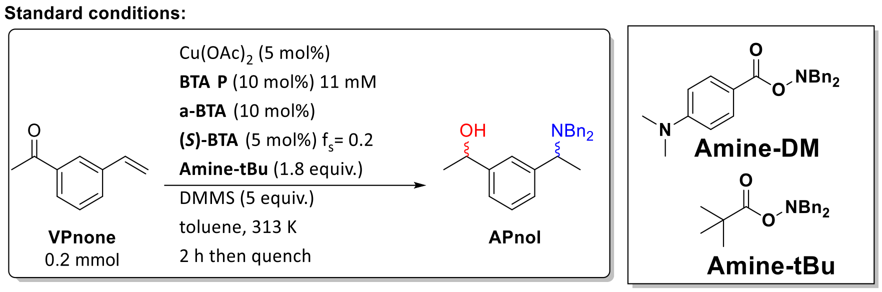

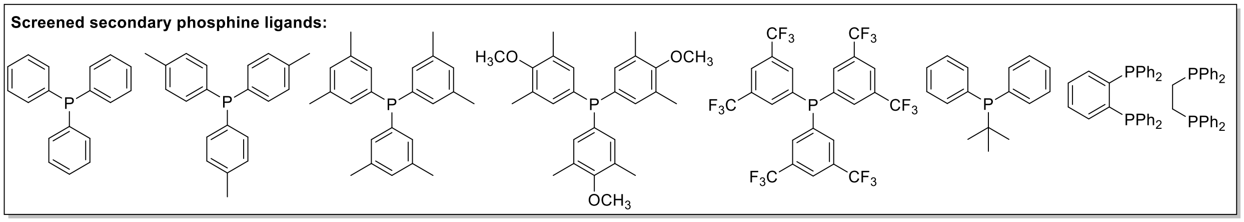

entry	deviation from standard conditions	NMR yield in APnol (± 5%)	% of main stereoisomer of APnol	ee, dr	ee1 (HS), ee2 (HA)
1	no	66%	77% (*R*,*S*)	97%, 3.6:1	68%, 83%
2	**Amine-DM** instead of **Amine-tBu**	63%	76% (*R*,*S*)	96%, 3.4:1	68%, 80%
3	+PPh_3 (5 mol%)	63%	73% (*R*,*S*)	95%, 2.9:1	64%, 77%
4	+$P(4\text{-}CH_3\text{-}C_6H_4)_3$ (5 mol%)	67%	71% (*R*,*S*)	93%, 2.7:1	64%, 72%
5	+$P(3,5\text{-}(CH_3)_2\text{-}C_6H_3)_3$ (5 mol%)	67%	70% (*R*,*S*)	93%, 2.7:1	67%, 69%
6	+$P(3,5\text{-}(CH_3)_2\text{-}4\text{-}OCH_3\text{-}C_6H_2)_3$ (5 mol%)	73%	72% (*R*,*S*)	94%, 2.9:1	67%, 73%
7	+$P(3,5\text{-}(CF_3)_2\text{-}C_6H_3)_3$ (2.5 mol%)	71%	78% (*R*,*S*)	96%, 3.8:1	70%, 83%
8	+$P(3,5\text{-}(CF_3)_2\text{-}C_6H_3)_3$ (5 mol%)	76%	78% (*R*,*S*)	96%, 3.8:1	69%, 84%
9	+$P(3,5\text{-}(CF_3)_2\text{-}C_6H_3)_3$ (10 mol%)	72%	77% (*R*,*S*)	96%, 3.9:1	71%, 83%
10	+*t*$BuPPh_2$ (5 mol%)	61%	73% (*R*,*S*)	95%, 3.0:1	63%, 79%
11	+$PPh_2CH_2CH_2PPh_2$ (5 mol%)	51%	65% (*R*,*S*)	89%, 2.2:1	58%, 64%
12	+*o*-$PPh_2C_6H_4PPh_2$ (5 mol%)	65%	64% (*R*,*S*)	89%, 2.2:1	65%, 56%
13	+$P(3,5\text{-}(CF_3)_2\text{-}C_6H_3)_3$ (5 mol%), under N_2	76%	79% (*R*,*S*)	97%, 4.1:1	71%, 84%
14	+$P(3,5\text{-}(CF_3)_2\text{-}C_6H_3)_3$ (5 mol%), without **a-BTA**[a]	61%	70% (*R*,*S*)	90%, 2.7:1	72%, 60%
15	+$P(3,5\text{-}(CF_3)_2\text{-}C_6H_3)_3$ (5 mol%), with **a-BTA** (5 mol%) instead of 10 mol%[b]	58%	76% (*R*,*S*)	96%, 3.4:1	68%, 80%

[a] f_s= 0.33. [b] f_s= 0.25. Control experiments for probing the importance of **a-BTA** are highlighted in grey. See below the comments on the catalytic screening of the different parameters.

Comments on Supplementary Table 1:

Influence of the amine electrophile: Comparable catalytic performance is observed between 4-(((dibenzylamino)oxy)carbonyl)-*N*,*N*-dimethylaniline (**Amine-DM**), an electron-rich amine transfer reagent commonly employed in Cu-H based processes,[1] and **Amine-tBu** (entry 2). The latter was selected for further screening given its higher solubility under our reaction conditions (toluene, 313 K).

Influence of the secondary ligand: A set of tertiary phosphines was next evaluated as secondary ligands, a common strategy used to enhance the performance of Cu-H type catalysts.[1,2] Triphenylphosphine (entry 3), electron-rich monophosphines (entries 4-6, 10) and tested diphosphine ligands (dppe and dppbz, entries 11-12) exhibit either no or detrimental effect. However, a slight but significant improvement in the yield in **APnol**, is observed upon addition of tris[3,5-bis(trifluoromethyl)phenyl]phosphine ($P(3,5\text{-}(CF_3)_2\text{-}C_6H_3)_3$) (entries 7-9). One equivalent of $P(3,5\text{-}(CF_3)_2\text{-}C_6H_3)_3$ relatively to copper proved to be the best stoichiometry (entry 8).

Influence of the atmosphere: Conducting the reaction under nitrogen does not significantly change the catalytic outcome of the concomitant process (entry 13).

Influence of **a-BTA**: From the ee of the individual steps, it can be seen that **a-BTA** significantly improves the ee of the HA reaction (compare ee2 in entries 1 and 14) but not of the HS step. The role of **a-BTA** for the cascade reaction appears to be similar to that observed previously in the HA of styrene.[3] The amount of **a-BTA** in the catalytic mixtures can be decreased by two, *i.e.* [**a-BTA**]:[**BTA P**]= 0.5 instead of 1 in the standard conditions, leading to a small decrease of the yield but to the same selectivity (entry 15), consistently with our recent findings.[4]

Supplementary Table 2. HS/HA cascade transformation of VPnone with BTA helical catalysts (concomitant reactions): screening of aromatic solvents.

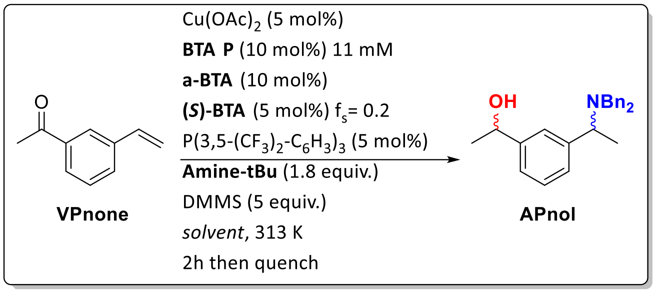

entry	solvent	NMR yield in APnol (± 5%)	% of main stereoisomer of APnol	ee, dr	ee1 (HS), ee2 (HA)
1	toluene	76%	78% (*R*,*S*)	96%, 3.8:1	69%, 84%
2	trifluorotoluene	82%	75% (*R*,*S*)	98%, 3.2:1	61%, 88%
3	4-xylene	73%	77% (*R*,*S*)	94%, 3.8:1	69%, 80%
4	mesitylene	69%	79% (*R*,*S*)	98%, 4.1:1	70%, 87%
5	chlorobenzene	62%	74% (*R*,*S*)	96%, 3.1:1	66%, 78%

Supplementary Table 3. Isolated yields for the HS/HA cascade transformation of VPnone with BTA helical catalysts under optimized conditions (concomitant reactions).

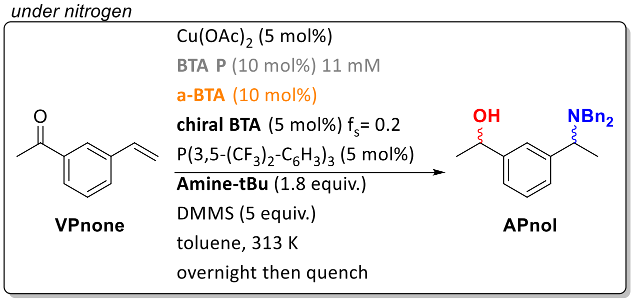

entry	schematic representation of the catalyst	isolated yield in **APnol**	% of main stereoisomer of **APnol**	ee, dr	ee1 (HS), ee2 (HA)
1	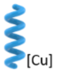	51%	77% (*R*,*S*)	96% (*R*,*S*), 3.6:1	68%, 83%
2	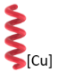	48%	79% (*S*,*R*)	97% (*S*,*R*), 4.2:1	-70%, -86%

Selecting the optimized conditions of Table 1, the concomitant HS/HA reaction was performed under nitrogen on an approximatively 1 mmol scale yielding **(*R*,*S*)-APnol** in 51% isolated yield,[1] 96% ee and a dr of 3.6:1 (entry 1). Engaging **(*R*)-BTA** (red helix) instead of **(S)-BTA** (blue helix) in the catalytic mixture leads to **(*S*,*R*)-APnol** with similar yield and selectivity as expected for homochiral BTA helical catalysts adopting opposite screw-sense preferences (entry 2).

[1] The fact that the isolated yield in **APnol** is lower than the NMR yield is due to difficult separation of **APnol** from **EPnol** and **VPnol**.

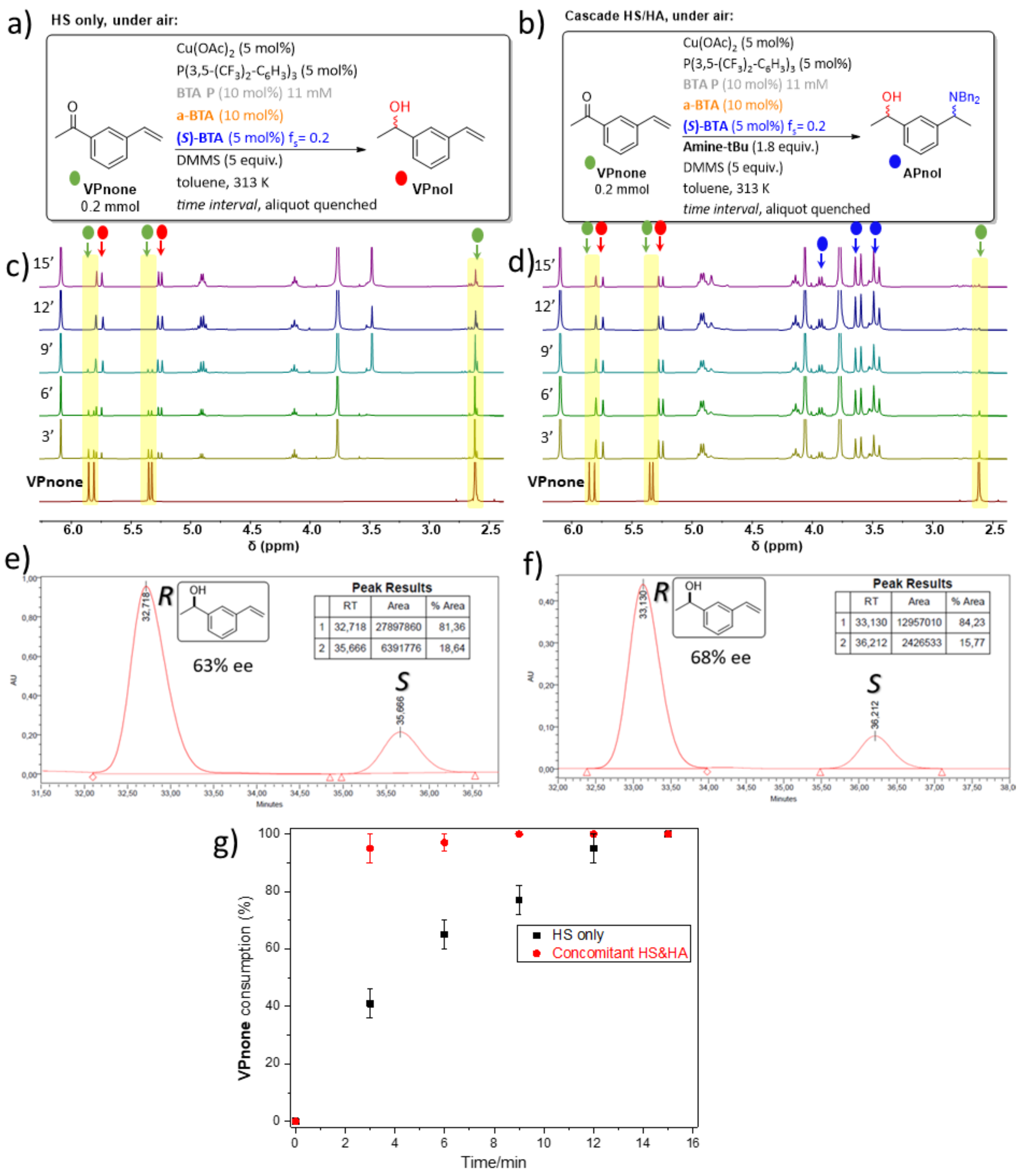

Supplementary Fig. 4. Monitoring of the hydrosilylation only and of the hydrosilylation/hydroamination concomitant reactions under air. Reaction schemes (a-b), ^{1}H NMR spectra (c-d) (**VPnone**, **VPnol** and **APnol** signals are labelled with green, red and blue ovals, respectively), and HPLC traces of isolated **VPnol** (e-f) from HS only and concomitant HS/HA reactions, respectively. Plot of the consumption of the ketone function (δ= 2.6 ppm) in the HS only and concomitant HS/HA reactions *versus* time as deduced from the ^{1}H NMR spectra in S2c and S2d (g). Error bars reflect the uncertainty in the integration of the NMR signal related to hydrogens of the vinyl group.

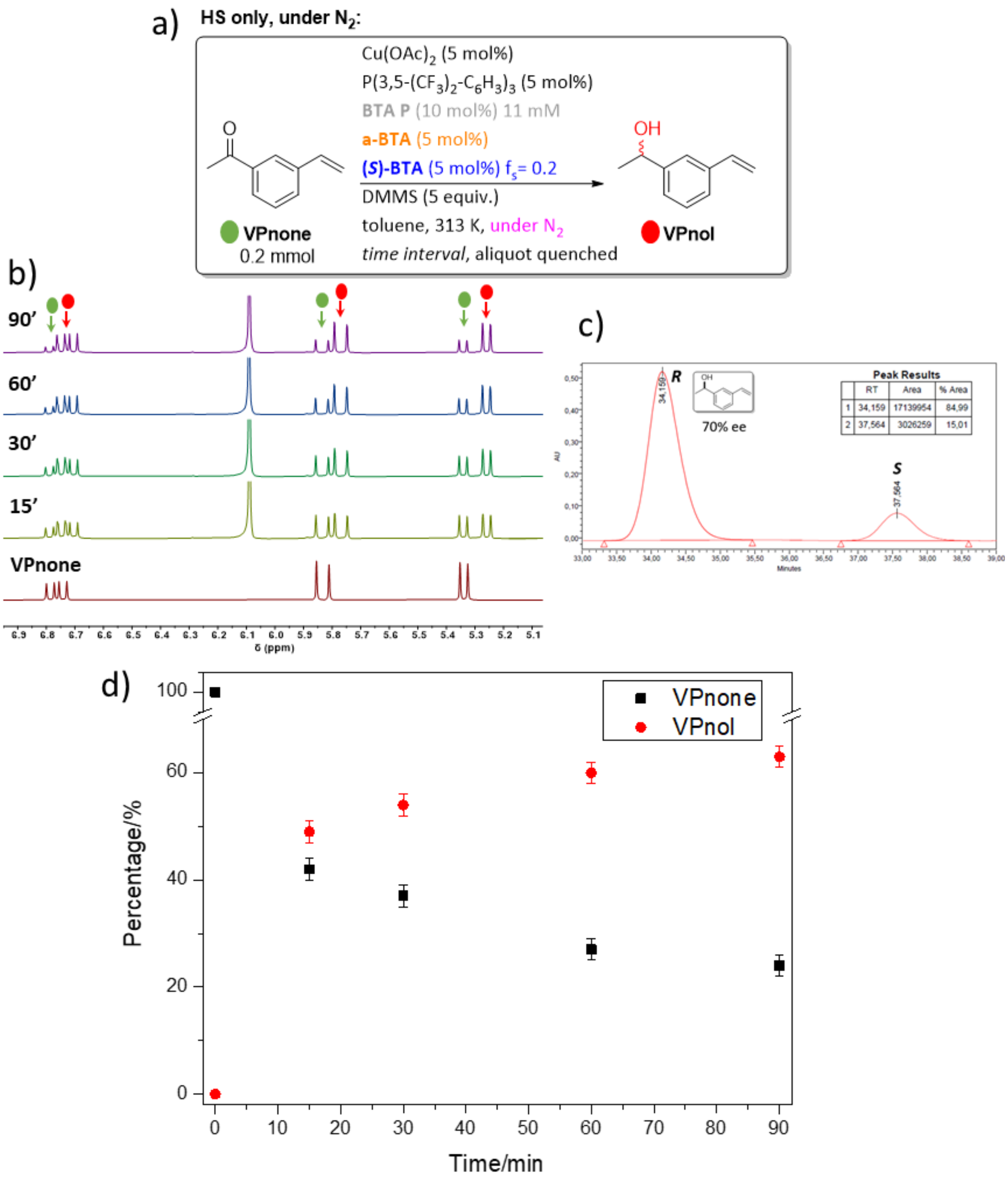

Supplementary Fig. 5. Monitoring of the hydrosilylation only reaction under nitrogen. Reaction scheme (a), ^{1}H NMR spectra (b), and HPLC trace of isolated **VPnol** (c). Plot of the consumption of **VPnone** and the production of **VPnol** over time under these conditions (d). Error bars reflect the uncertainty in the integration of the NMR signal related to hydrogens of the vinyl group.

Comments on the data of Supplementary Figs. 4-5 about the rate and selectivity of the hydrosilylation reaction: In absence of **Amine-tBu** (hydrosilylation only reaction), we found out that **VPnone** is fully consumed in *ca.* 12 minutes and that **(*R*)-VPnol** is formed in *ca.* 70% yield and 63% ee; **EPnol** (*ca.* 12%) is detected as the main byproduct (Supplementary Fig. 4a,c,e,g). Upon comparing NMR traces for the hydrosilylation only reaction and the hydrosilylation/hydroamination concomitant process (Supplementary Fig. 4b,d,f,g), it appears that the rate of the ketone reduction is drastically higher in the latter case, *i.e.* that hydrosilylation of the ketone function occurs more rapidly when **Amine-tBu** is present from the beginning in the catalytic mixture. It suggests different catalytic active species for the ketone reduction depending whether the amine transfer reagent is present or not. In addition, the rate of the hydrosilylation reaction is even more reduced upon conducting the reaction under nitrogen (Supplementary Fig. 5). Under these conditions, the reaction slows down after 15 minutes and even seems to halt after 60 minutes (*ca.* 25% of **VPnone** is not consumed). Whilst the exact reason of this dramatic rate decrease is not clear at that time, previous examples in the literature point out rate acceleration of CuH-catalyzed processes under air.[5–9] However, it is important to note that, in all these conditions, the enantioselectivity of the hydrosilylation reaction remains virtually identical (63%-70% ee). These data indicate that the reaction time of the hydrosilylation step must be adapted for the sequential hydrosilylation/hydroamination process depending on the conditions.

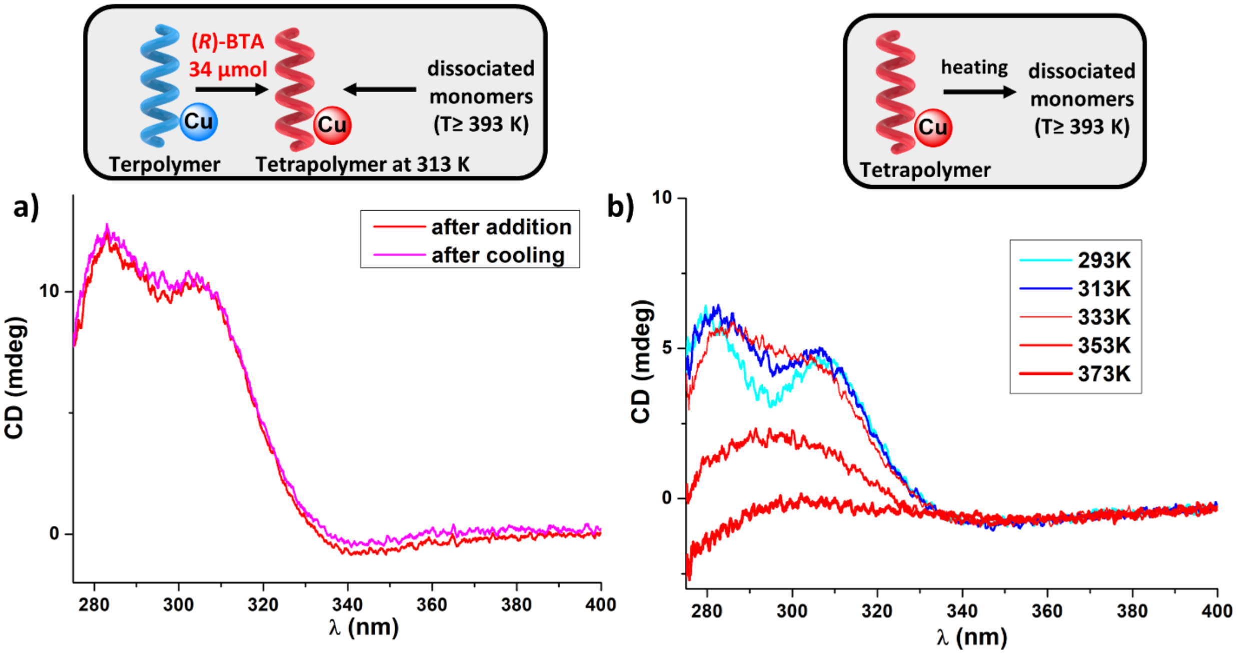

Supplementary Fig. 6. Switching the handedness of the BTA helical precatalysts. a) Comparison of the CD spectra (313 K) of the tetrapolymer upon formation from preexisting terpolymer and upon cooling from the monomeric state. Composition: **BTA P** (22 µmol), Cu(OAc) (11 µmol), P(3,5-$(CF_3)_2$-$C_6H_3)_3$ (11 µmol), **(*S*)-BTA** (11 µmol), **(*R*)-BTA** (34 µmol), **a-BTA** (22 µmol). b) Variable-temperature CD measurements of the solution containing the tetrapolymer (heating, 1 $K.min^{-1}$). Composition: the solution in a is diluted two times in order to get a less viscous solution that is easier to handle.

Comments on the data of Supplementary Fig. 6: CD spectra of the supramolecular tetrapolymer upon formation from preexisting terpolymer or after cooling from the monomeric state are virtually identical thus indicating that the thermodynamic state is reached after switching of the helix handedness. Variable temperature CD experiments in Supplementary Fig. 6b demonstrate that a (non-CD active) monomeric or quasi monomeric state is reached upon heating to 373 K.

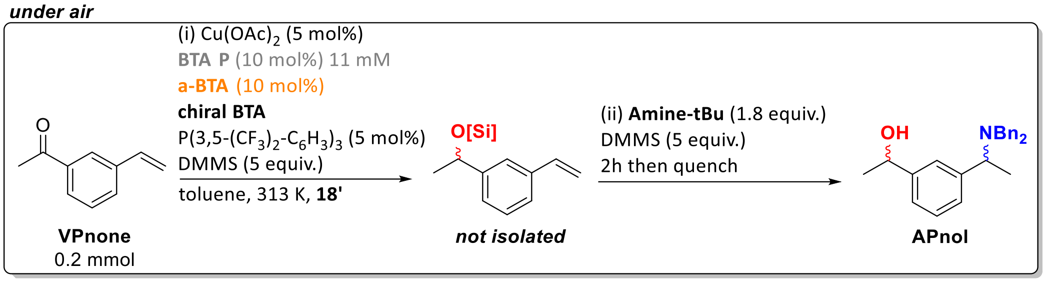

entry	schematic representation of the helical catalyst	chiral BTA	NMR yield in **APnol** (±10%)	% of the main stereoisomer of **APnol**	ee, dr	ee1 (HS)	ee2 (HA)
1	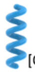 [Cu]	**(*S*)-BTA**	29%	72% (*R*,*S*)	89% (*R*,*S*), 3.2:1	59%	76%
2	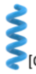 [Cu]	**(*S*)-BTA/(*R*)-BTA** (50% ee)	27%	72% (*R*,*S*)	93% (*R*,*S*), 3.0:1	64%	75%

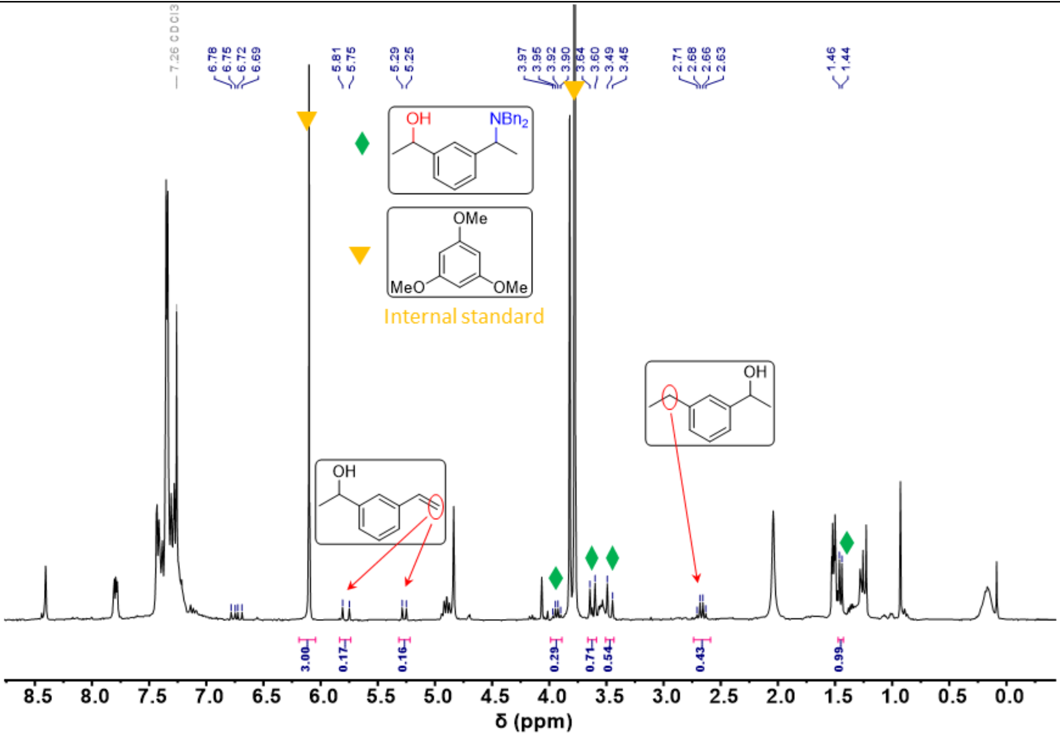

Supplementary Fig. 7. HS/HA cascade transformation of **VPnone** with the BTA helical catalyst having the same handednesses for both reactions (sequential process). The total time of 18 minutes for the hydrosilylation step corresponds to the time of HS step + time for the handedness switch in the selectivity-reversal experiments of Table 2, entries 1-4. Reaction scheme and ^{1}H NMR spectrum of the crude sample. In addition to **APnol** obtained in low yield (29%), 22% and 17% of **EPnol** and **VPnol** are detected. The optimal selectivity is reached whatever chiral induction occurs through a single enantiomer of the BTA monomer (similarly to the initial conditions before inversion of the catalyst handedness) or by means of a scalemic mixture of the BTA monomers (similarly to the conditions after inversion of the catalyst handedness).

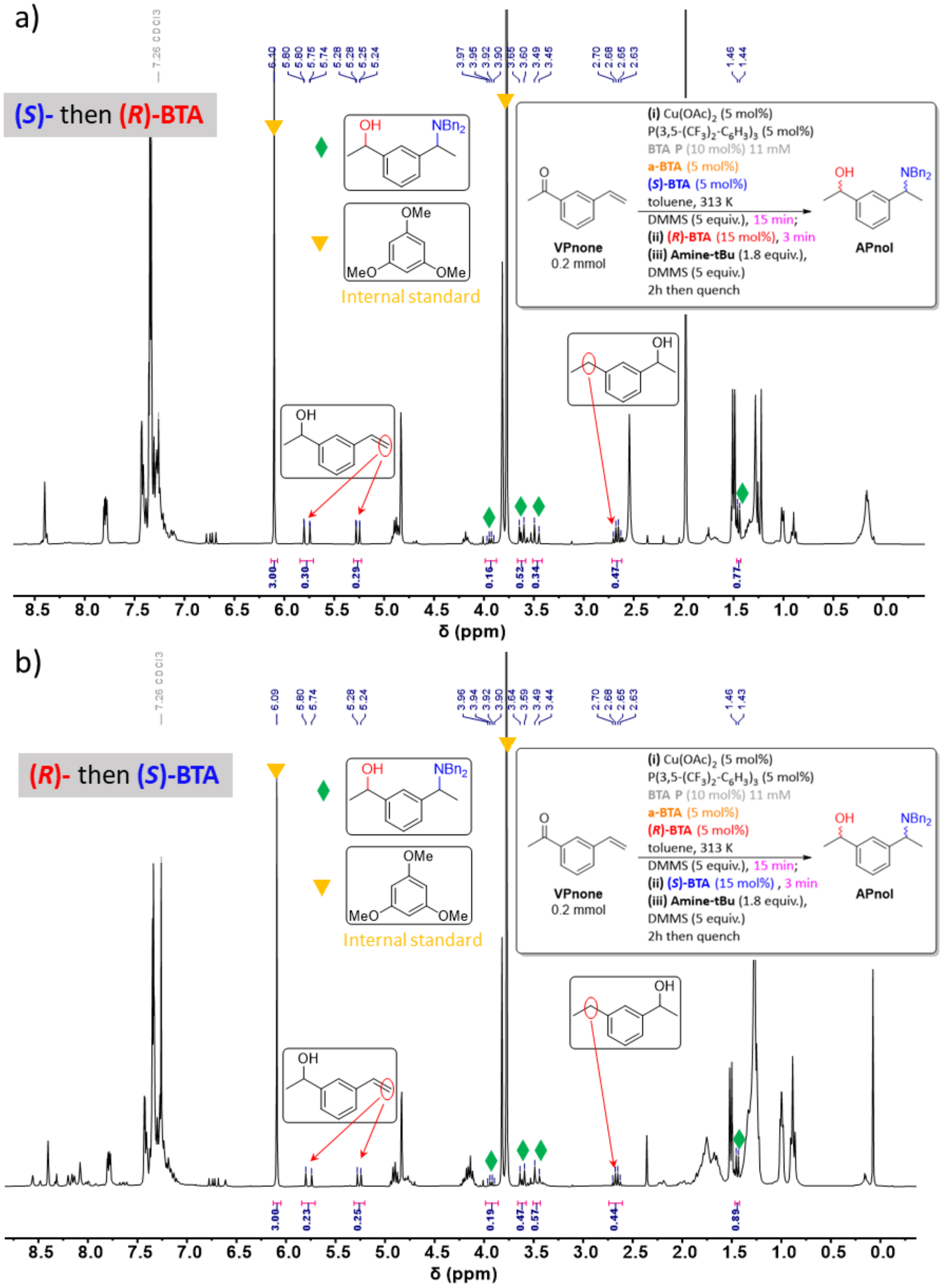

Supplementary Fig. 8. HS/HA cascade transformation of **VPnone** with the BTA helical catalyst having opposite handednesses for both reactions (sequential process, Table 2, entries 3 and 4). Reaction schemes and ^{1}H NMR spectra of the crude samples. Reactions are performed without exclusion of air (a) **(*S*)-** then **(*R*)-BTA** and (b) **(*R*)-** then **(*S*)-BTA**. In addition to **APnol** obtained in low yield (*ca.* 20%), *ca.* 22% and 25% of **EPnol** and **VPnol** are detected, respectively. The lower yield of the sequential process is not related to the addition of the switching agent (see Supplementary Fig. 7), *i.e.* the chiral monomer, but is rather due to catalyst deactivation occurring under air.

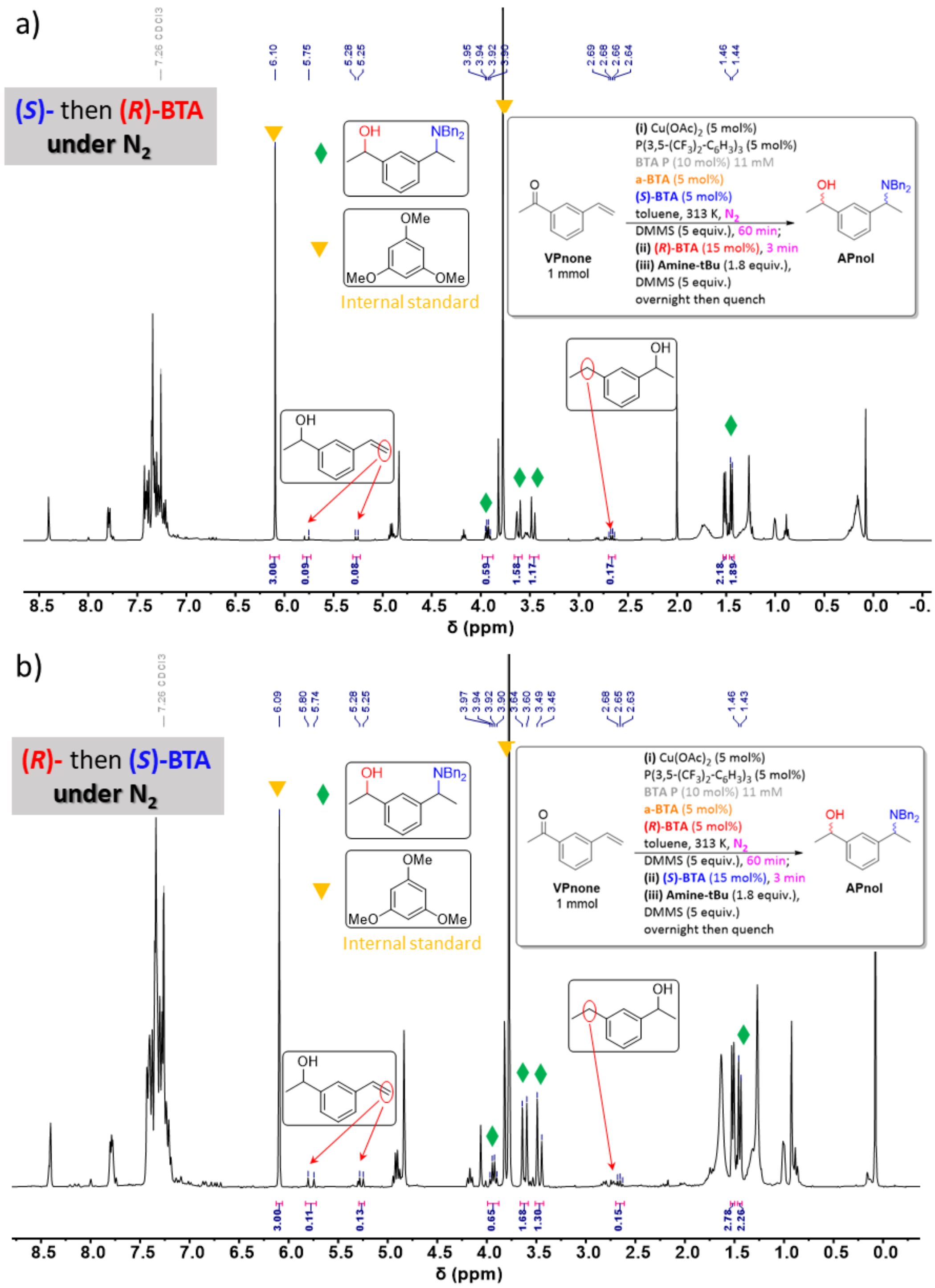

Supplementary Fig. 9. HS/HA cascade transformation of **VPnone** with the BTA helical catalyst having opposite handednesses for both reactions (sequential process, Table 2, entries 5 and 6). Reaction schemes and ^{1}H NMR spectra of the crude samples. Reactions are performed under anhydrous conditions and exclusion of air. (a) **(*S*)-** then **(*R*)-BTA** and (b) **(*R*)-** then **(*S*)-BTA**. In addition to **APnol** obtained in good yield (65±5%), *ca.* 10% and 8% of **VPnol** and **EPnol** are detected, respectively, confirming that catalyst deactivation is minimized under these conditions relatively to the sequential reactions conducted under air (Supplementary Fig. 8).

Supplementary Table 4. Screened conditions for the sequential HS/HA transformation of VPnone involving the BTA helical catalyst having opposite handednesses for both reactions.[a]

under nitrogen, conditions of entries 1 and 2

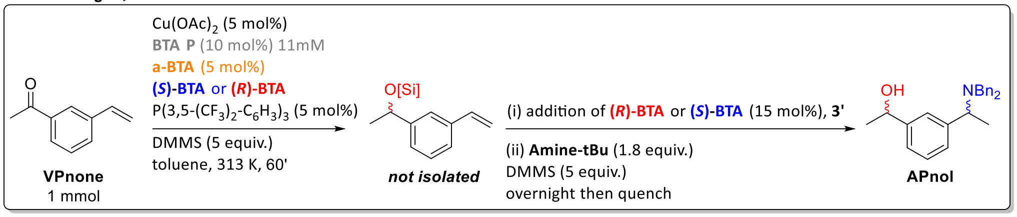

entry	schematic representation of the helical catalyst	modifications relatively to the scheme	NMR yield (±5%), isolated yield in **APnol**	% of main stereoisomer of **APnol**	ee, dr	ee1 (HS), ee2 (HA)
1	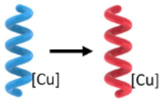	none	63%, 38%	50% (***R***,***R***)	79% (***R***,***R***), 1.3:1	20%, -68%
2	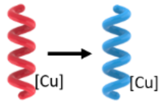	none	65%, 40%	51% (***S***,***S***)	81% (***S***,***S***), 1.3:1	-21%, 71%
3	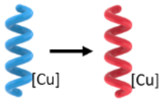	120' for HS (instead of 60')	61%, 42%	48% (***R***,***R***)	76% (***R***,***R***), 1.2:1	16%, -68%
4	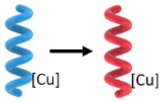	an additional 2.5 equiv. of DMMS added after 60' + 120' for HS	64%, 35%	54% (***R***,***R***)	80% (***R***,***R***), 1.5:1	37%, -59%

[a] Reactions have been conducted on 1 mmol scale of **VPnone** under strict exclusion of air and water (see catalytic procedures). Both NMR (via an internal standard on a crude sample) and isolated yields in **APnol** are provided. The enantiomeric excess and diastereomeric ratio are obtained from the chiral HPLC analyses of the purified samples. Entries 1 and 2 correspond to entries 5 and 6 of Table 2.

Comments on Supplementary Table 4**:** The lower selectivities in (***R*,*R*)-APnol** and (***S*,*S*)-APnol** for entries 1 and 2 relatively to the reaction performed under O_2 is due to an estimated conversion of **VPnone** of *ca.* 70% before the stereochemical switch, consistent with the reaction monitoring in Supplementary Fig. 5.[2] Our attempt to increase **VPnone** conversion by increasing the time of the HS step to 2 hours was not successful since an identical selectivity in **APnol** was obtained (entry 3). To circumvent this apparent inhibition of the HS catalyst, additional DMMS was added after 1 hour (entry 4). It indeed leads to a higher conversion of **VPnone** before the stereochemical switch (ee1 of 37%, estimated conversion of *ca.* 80%) but at the expense of a lower selectivity for the HA step indicating that the stereochemical switch was not complete at the onset of the HA reaction. It is likely that the increased amount in DMMS engaged in that specific case (which herein represents 15% of the medium in volume) leads to less soluble and thus less dynamic BTA terpolymers. In overall, performing the reaction under nitrogen significantly increases the yield of the sequential reaction but drastically changes the kinetics of the HS step that makes difficult to achieve optimal selectivity under these conditions.

[2] The calculations are made by considering that conversion occurs only before or after the switch, i.e. with catalysts displaying opposite enantioselectivities. These values are actually lower limit values if one considers that conversion occurs also during the stereochemical switch, e.g. with a racemic catalyst.

Control of the selectivity of the reaction by the catalyst (Supplementary Figs. 10-11)

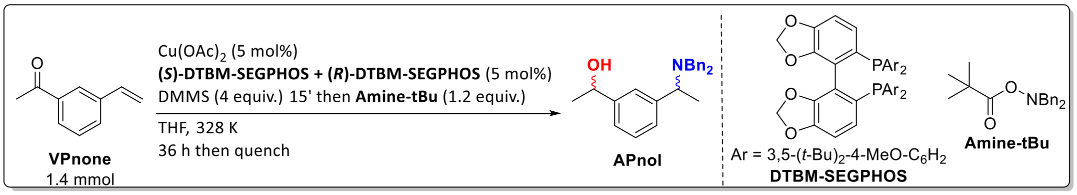

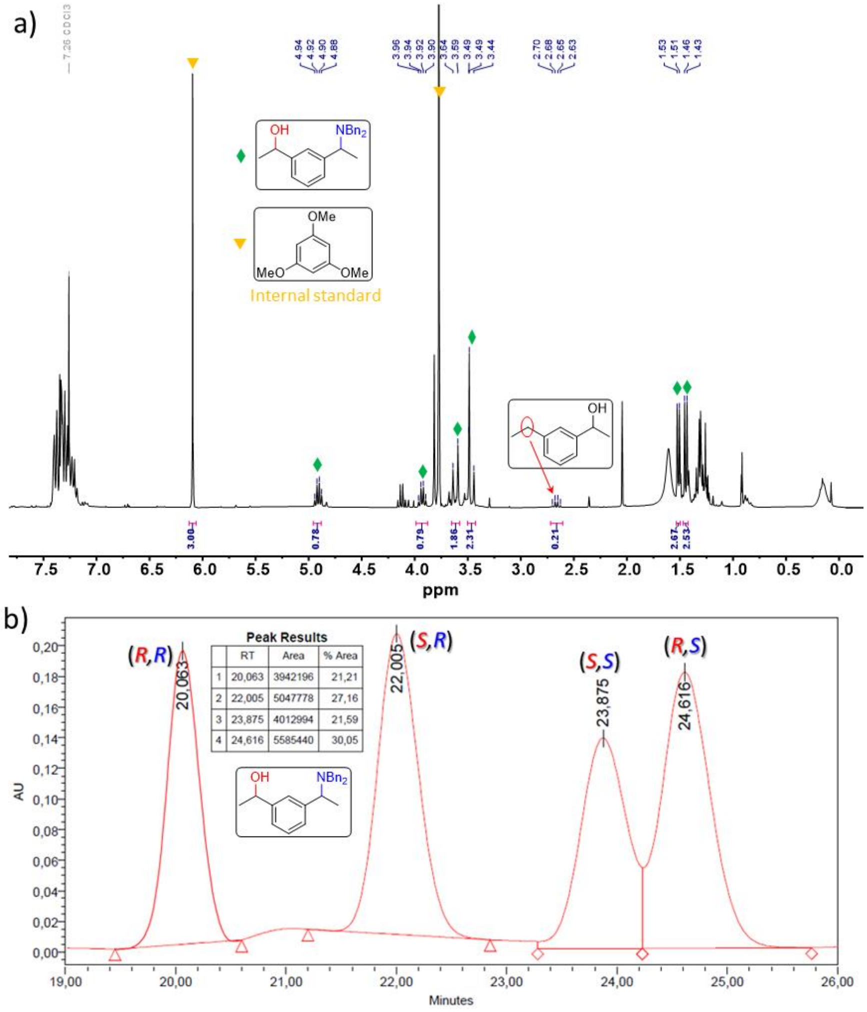

Supplementary Fig. 10. Sequential hydrosilylation (HS) and hydroamination (HA) cascade reaction of **VPnone** catalyzed by **(*rac*)-DTBM-SEGPHOS**. (a) ^{1}H NMR spectrum of the crude sample with selected NMR signals for **APnol** (green diamonds) and **EPnol** (*ca.* 10%). (b) Chiral HPLC trace of **APnol** present in the crude sample. Absolute configuration is established by the intrinsic selectivity of **(*S*)-DTBM-SEGPHOS** and **(*R*)-DTBM-SEGPHOS** and calculations as explained below.

Interpretation of the data: **VPnone** was mixed with a 1:1 mixture of **(*S*)-DTBM-SEGPHOS** and **(*R*)-DTBM-SEGPHOS**, in presence of [Cu(OAc)$_2$], with dimethoxymethylsilane (DMMS) and *N*,*N*-dibenzyl-*O*-pivaloylhydroxylamine (**Amine-tBu**) as the reducing and aminating agents, respectively. The reaction conducted in a sequential manner, *i.e.* by engaging **Amine-tBu** after completion of the hydrosilylation of the ketone function, yields the expected amino alcohol, 1-[3-(1-dibenzylaminoethyl)]-acetophenol (**APnol**), as a 1.0/1.3/1.0/1.4 mixture of the four stereoisomers according to its HPLC trace. The diastereomeric ratio (dr), close to 1:1, is an indication that the selectivity of the reaction is mostly controlled by the catalyst, not the substrate.

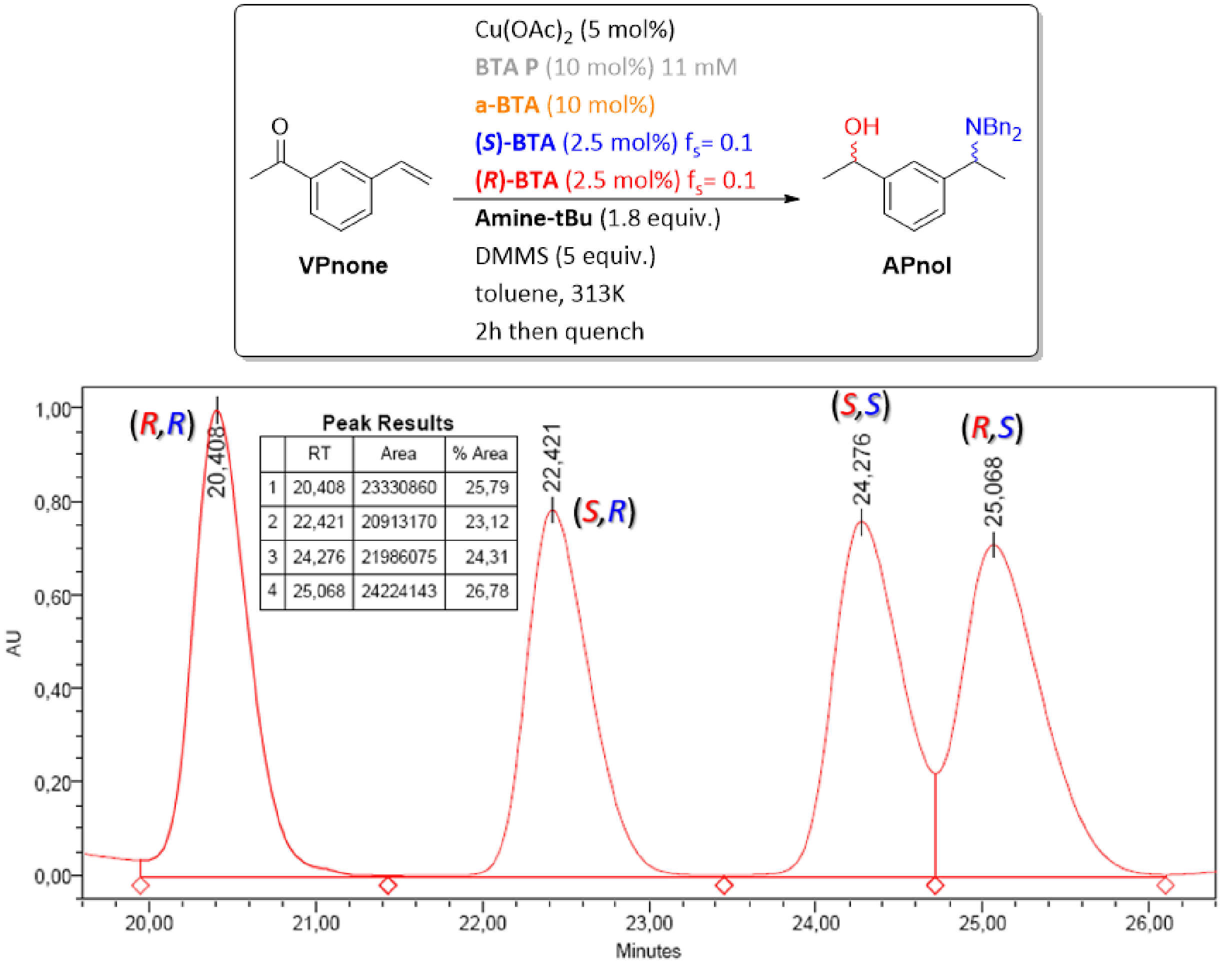

	RT	Area	% Area
1	20,408	23330860	25,79
2	22,421	20913170	23,12
3	24,276	21986075	24,31
4	25,068	24224143	26,78

Supplementary Fig. 11. Sequential hydrosilylation (HS) and hydroamination (HA) cascade reaction of **VPnone** catalyzed by a BTA helical catalyst incorporating an equimolar mixture of **(*S*)-BTA** and **(*R*)-BTA** as indicated in the reaction scheme. HPLC trace of isolated **APnol**.

Interpretation of the data: When **(*S*)-BTA** and **(*R*)-BTA** are engaged in equal amounts in the catalytic mixture, the resulting racemic helices furnish **APnol** as a virtually perfect equimolar mixture of its four stereoisomers, emphasizing the possibility to control the selectivity by the catalyst. This experiment also discards that kinetic resolution (leading to incomplete conversion of **VPnol** intermediate) might play a role in the outcome of the asymmetric reaction.

HPLC separation and characterization of the enantiopure stereoisomers of APnol (Supplementary Figs. 12-15, Supplementary Table 5)

Analytical separation: A racemic mixture of **APnol** stereoisomers (obtained from catalysis experiment performed with a BTA helical catalyst composed of an equimolar mixture of **(*S*)-BTA** and **(*R*)-BTA**, Fig. S8) was dissolved in a mixture of heptane and ethanol, injected on the chiral column, and detected with an UV detector at 254 nm. The flow-rate is 1 mL/min.

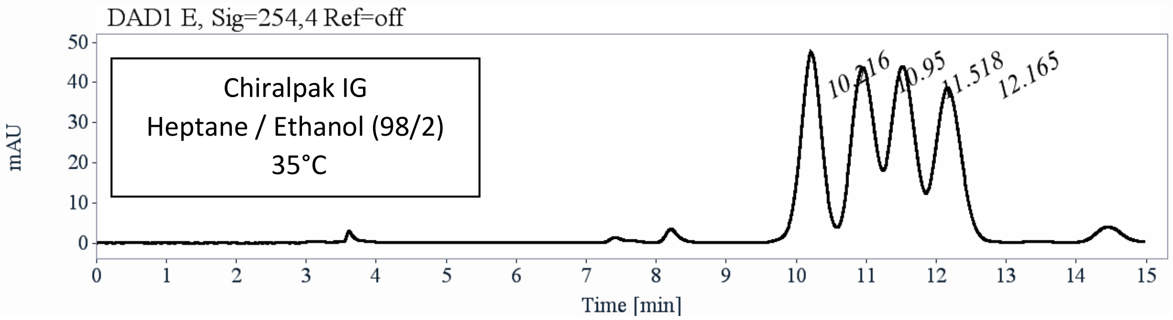

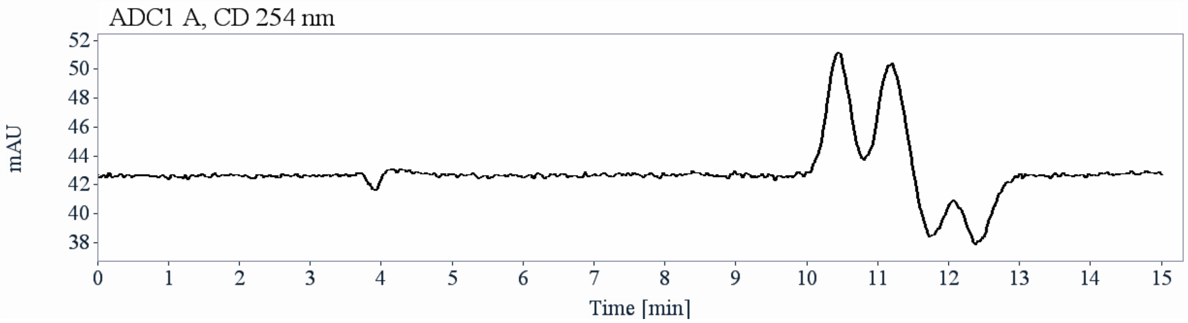

RT [min]	Area	Area%	
10.22	894	23.78	1st eluted on Chiralpak IG ($\square_D$ +104) (+, CD 254 nm)
10.95	911	24.25	2nd eluted on Chiralpak IG ($\square_D$ + 68) (+, CD 254 nm)
11.52	1003	26.69	3rd eluted on Chiralpak IG ($\square_D$ - 104) (-, CD 254 nm)
12.16	950	25.28	4th eluted on Chiralpak IG ($\square_D$ - 68) (-, CD 254 nm)
Sum	3758	100.00	

Supplementary Fig. 12. HPLC analysis of a racemic mixture of APnol.

Preparative separation: The separation was done in three steps: **(1)** on Chiralpak AD-H (250x10 mm), hexane/ethanol (97/3) as mobile phase, flow-rate= 5 mL/min, UV detection at 230 nm, to separate the (+)-isomers from the (-)-isomers. **(2)** on Lux-Cellulose-1 (250x10 mm), hexane/isopropanol (95/5) as mobile phase, flow-rate= 5 mL/min, UV detection at 254 nm, to separate the isomer with α_D= +104 from the isomer with α_D= +68. (**3**) on Chiralpak AD-H (250 x 10 mm), hexane/isopropanol (95/5) as mobile phase, flow-rate= 5 mL/min, UV detection at 254 nm, to separate the isomer with α_D= -104 from the isomer with α_D= -68.

Collected stereoisomers: • 1st eluted on Chiralpak IG, 10.22 min, (α_D +104): 10 mg with dr > 100:1 and ee > 99.5%. • 2nd eluted on Chiralpak IG, 10.95 min, (α_D + 68): 15 mg with dr > 100:1 and ee > 99.5%. • 3rd eluted on Chiralpak IG, 11.52 min, (α_D - 104): 3.8 mg with dr > 100:1 and ee > 99.5%. • 4th eluted on Chiralpak IG, 12.16 min, (α_D - 68): 11 mg with dr> 100:1 and ee > 99.5%.

Supplementary Table 5. Optical rotations of the enantiopure stereoisomers.[a]

λ (nm)	**(*R*,*R*)-APnol**, first eluted $[\alpha]_{\lambda}^{25}$ (CH_2Cl_2, c =0.54)	**(*S*,*R*)-APnol**, second eluted $[\alpha]_{\lambda}^{25}$ (CH_2Cl_2, c =0.5)	**(*S*,*S*)-APnol**, third eluted $[\alpha]_{\lambda}^{25}$ (CH_2Cl_2, c =0.55)	**(*R*,*S*)-APnol**, fourth eluted $[\alpha]_{\lambda}^{25}$ (CH_2Cl_2, c =0.53)
589	+ 104	+ 68	- 104	- 68
578	+ 109	+ 71	- 109	- 71
546	+ 127	+ 84	- 127	- 84
436	+ 229	+ 156	- 229	- 156
405	+ 289	+ 200	- 289	- 200
365	+ 421	+ 300	- 421	- 300

[a] Optical rotations were measured on a Jasco P-2000 polarimeter with a halogen lamp (589, 578, 546, 436, 405 and 365 nm), in a 10 cm cell, thermostated at 298 K with a Peltier controlled cell holder. Absolute and relative configurations can be established as indicated in the general methods.

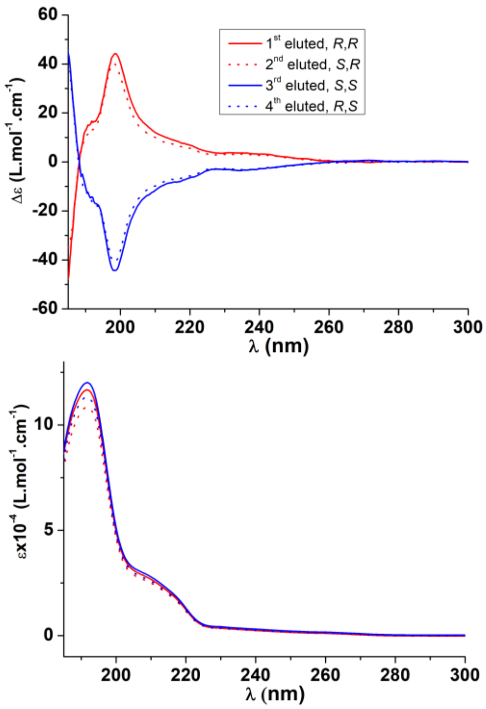

Supplementary Fig. 13. CD analysis of the enantiopure stereoisomers of APnol. ECD and UV spectra were measured on a JASCO J-815 spectrometer equipped with a JASCO Peltier cell holder PTC-423 to maintain the temperature at 298.0 ± 0.2°K. A CD quartz cell of 1 mm of optical pathlength was used. The CD spectrometer was purged with nitrogen before recording each spectrum, which was baseline subtracted. The baseline was always measured for the same solvent and in the same cell as the samples. The spectra are presented without smoothing and further data processing. Acquisition parameters: 0.1 nm as intervals, scanning speed 50 nm/min, bandwidth 2 nm, and 3 accumulations per sample. All separated stereoisomers were dissolved in acetonitrile (concentration≈ 0.16 mM). UV-Vis absorption spectra were extracted from CD analyses on each of the above samples and obtained after correction of the absorption of air, solvent, and cell contribution at the same temperature.
CD and UV-Vis spectra of **APnol** stereoisomers with their elution order (on Chiralpak IG) and absolute and relative configurations. CD spectra of the **(*R*,*R*)-APnol** and **(*S*,*R*)-APnol** stereoisomers on one side, and of the **(*S*,*S*)-APnol** and **(*R*,*S*)-APnol** stereoisomers on the other side, are hardly distinguishable.

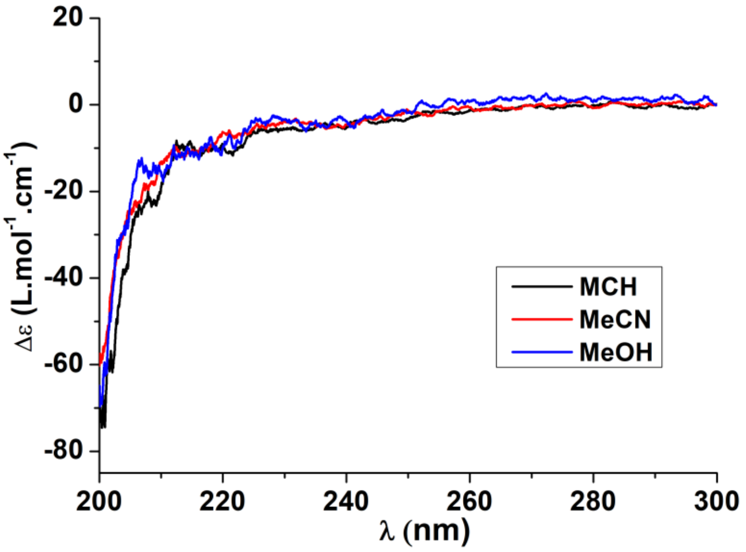

Supplementary Fig. 14. CD spectra of the fourth eluted stereoisomer, ***(R,S)*-APnol**, in various solvents. CD spectra were recorded with the CD spectrometer and conditions reported in the "General procedures and methods". CD spectra of ***(R,S)*-APnol** (0.15 mM) in methylcyclohexane (MCH), acetonitrile (MeCN) and methanol (MeOH). All CD spectra are identical thus indicating that the solvent does not significantly affect the chiroptical properties of the stereoisomers of **APnol**.

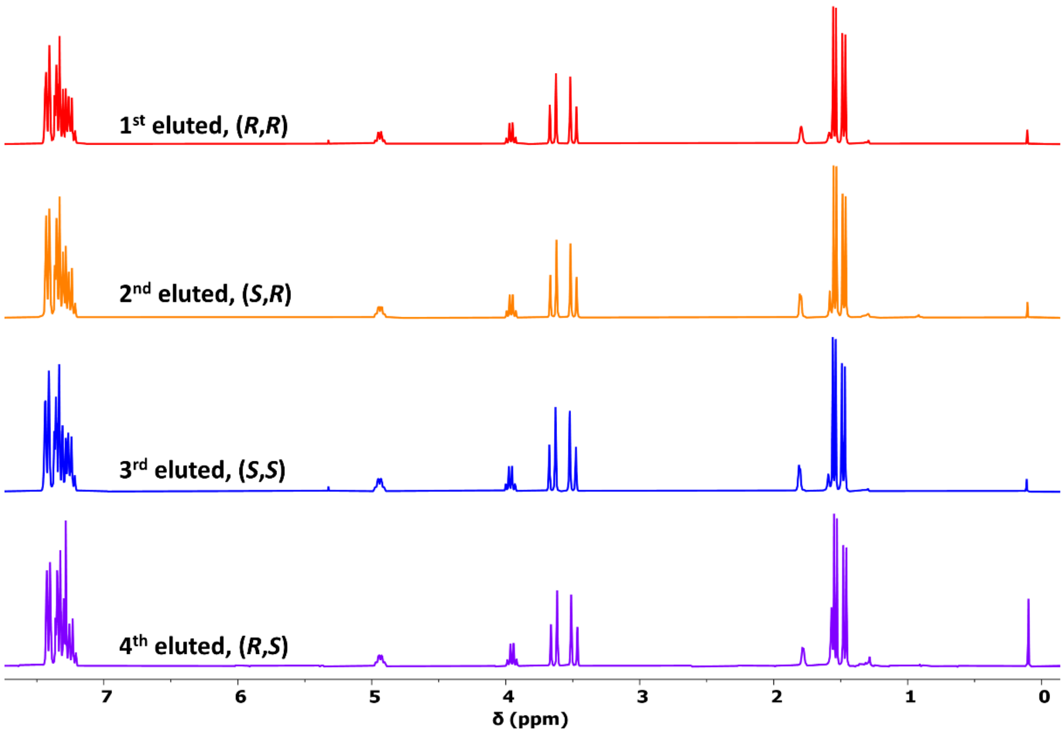

Supplementary Fig. 15. ^{1}H NMR spectra of the enantiopure stereoisomers: All separated stereoisomers were dissolved in $CDCl_3$ and analysed by ^{1}H NMR. All spectra are virtually identical. See further characterization data of **APnol** in "Characterization of the catalytic products".

Determination of the absolute and relative configurations of APnol (Supplementary Figs. 16-19)

(i) Cascade reaction conducted with enantiopure DTBM-SEGPHOS

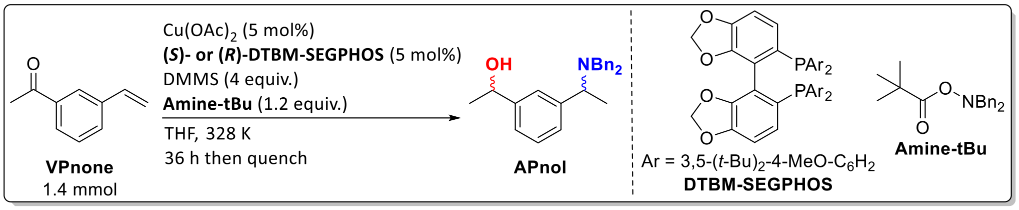

ligand	isolated yield in **APnol**	% of main stereoisomer of **APnol**	ee, dr	ee1 (HS), ee2 (HA)
**(*R*)-DTBM-SEGPHOS**	56%	90% (*R*,*S*)	99% (*R*,*S*), 9:1	84%, 94%
**(*S*)-DTBM-SEGPHOS**	50%	88% (*S*,*R*)	99% (*S*,*R*), 7.3:1	-80%, -94%

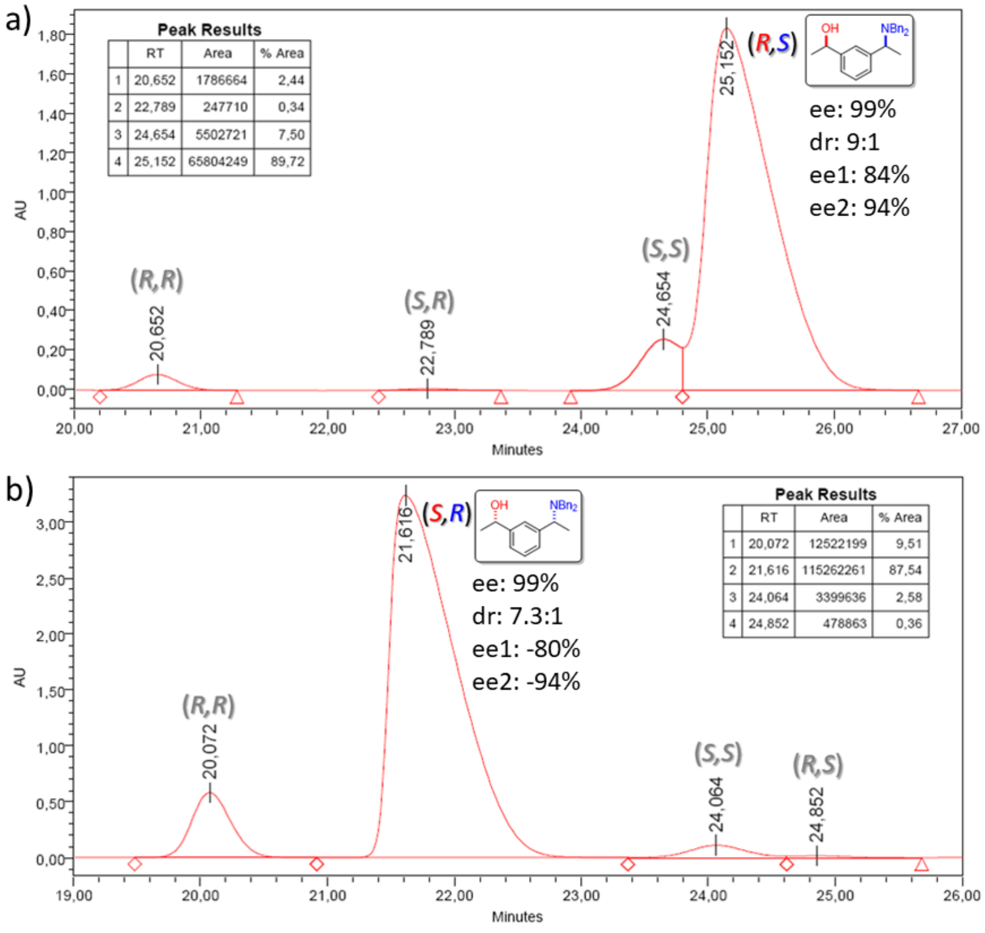

Supplementary Fig. 16. Asymmetric hydrosilylation/hydroamination cascade reaction of **VPnone** with DTBM-SEGPHOS as ligand (concomitant process). Table summarizing the catalytic results. Chiral HPLC traces of isolated **APnol** from reaction catalyzed by (a) **(*R*)-DTBM-SEGPHOS** and (b) **(*S*)-DTBM-SEGPHOS**.

Interpretation of the data: DTBM-SEGPHOS is a covalent diphosphine ligand widely investigated for reactions involving Cu-H active species.[10–12] However, cascade hydrosilylation (HS) /hydroamination (HA) reaction of non-conjugated vinyl and ketone functions have not been reported yet. Performing the hydrosilylation/hydroamination cascade reaction of **VPnone** with **(*R*)-DTBM-SEGPHOS** under conditions resembling those reported by Buchwald and co-workers for the asymmetric HS/HA of enals,[13] yields **APnol** in 56% yield, 99% enantiomeric excess (ee) and a diastereomeric ratio of 9:1. As expected, **(*S*)-DTBM-SEGPHOS** provides the corresponding enantiomer with similar yield and selectivity. In these reactions, 3-ethylacetophenol (**EPnol**) also forms in small amount, probably as a result of the protonation of the alkyl copper catalytic species by residual water (see **EPnol** characterization in the part "characterization of the catalytic products").[14,15] It is interesting to compare the catalytic selectivity of DTBM-SEGPHOS for the cascade transformation of **VPnone** to that previously reported for monofunctional substrates bearing either the ketone or the vinyl function. It can be concluded from the literature that **(*S*)-DTBM-SEGPHOS** yields (*S*)-acetophenol (96% ee)[2] and (*R*)-*N*,*N*-dibenzyl-1-phenylethan-1-amine (97% ee)[16] from the HS of acetophenone and the HA of styrene, respectively.[3] Therefore we expect the main isomer of the cascade reaction to be **(*S*,*R*)-APnol**. Calculations have been performed to definitely ascertain the absolute configuration.

(ii) MM/MD calculations

(*S*,*R*)-APnol was first built and submitted to an exhaustive conformational search. The seven torsions about single bonds that are responsible of conformational changes were systematically varied by steps of 60° or 120°, depending on the angle. The search was repeated by varying the conditions to build the successive conformers: using the structure of the initial conformer or of the previous one, and with phenyls at the same scale as the other parts of the molecule or having bond lengths reduced to one tenth (to avoid the formation of entangled rings). Each structure was optimized by MM, relaxing all degrees of freedom using the Smart Minimizer algorithm, with convergence criteria of 0.0001 $kcal.mol^{-1}$ and 0.005 $kcal.mol^{-1}.Å^{-1}$. About 89000 structures were generated in total, which were reduced to about 2000 after removing (i) structures whose chirality changed during the optimization process (154) and, (ii) redundant structures based on torsion ranges and energy differences (< 0.1 $kcal.mol^{-1}$). Among these ~2000 structures, 19 conformers account for 50% of the population, based on a Boltzmann distribution at 298 K. However, that distribution does not take entropy effects into account. To have a more realistic distribution, we performed a MD and counted the number of times the different conformers appeared during the dynamics. The MD was performed in the canonical (N,V,T) ensemble, using similar energy parameters as for MM. The Nose bath thermal coupling[17] was used to maintain the temperature at 298 K, with a coupling constant of 0.01, and the equations of motion were integrated by the Verlet velocity algorithm.[18] The MD lasted 2 μs with a 1-fs time step, and 20000 structures were saved. The analysis of the generated structures shows that about 50% of them can be related to 20 conformers obtained in the MM study (they differ from the MM local minima by small deviations in internal coordinates due to thermal agitation).

Then, we simulated UV-Vis absorption and CD spectra of **(*S*,*R*)-APnol** using a TD-DFT method with the hseh1pbe hybrid exchange correlation functional, the 6-311+g(d,p) basis set, and the first 200 excited states;

[3] Upon considering the ee of each step for CuH-catalyzed HS/HA reaction of **VPnone** in presence of DTBM-Segphos, it appears that the ee for the HA step is consistent with that obtained for the HA of styrene but the ee for the HS step is lower than that reported for the HS of acetophenone (see reference 2). This might due to the fact that the HS is performed at 295 K for acetophenone versus 313 K for **VPnone**.

the intensities were broadened with a standard deviation of 4 nm. The calculations were performed using the Gaussian16 package [M. J. Frisch, *et al.*, Gaussian 16 Revision A.03, Gaussian Inc., Wallingford, CT, USA, 2016.]. Three sets of spectra were built, called MM19, MD20 and MD29: MM19 corresponds to the 19 most stable MM conformers, each one contributing to the total intensity by its relative Boltzmann weight; MD20 corresponds to the 20 MM conformers that occur most frequently in MD, each one contributing to the total intensity by its relative weight in the MD population; MD29 is similar to MD20, except that to calculate the signal of the most frequent conformer, instead of using a MM local minimum, ten structures of this conformer were selected in the MD trajectory, and their signals were averaged. These additional calculations were used to investigate the effect of deviations in internal coordinates due to thermal agitation, and were applied to the most frequent conformer only to limit computational time. The spectra are displayed below. The solvent has no influence on the CD signal of **APnol** (**Supplementary Fig. 14**) thus strengthening the validity of the simulated CD spectra.

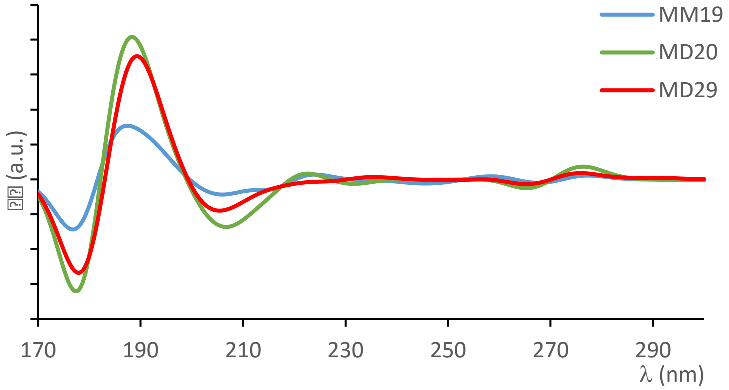

Supplementary Fig. 17. Simulated CD spectra of **(*S*,*R*)-APnol**

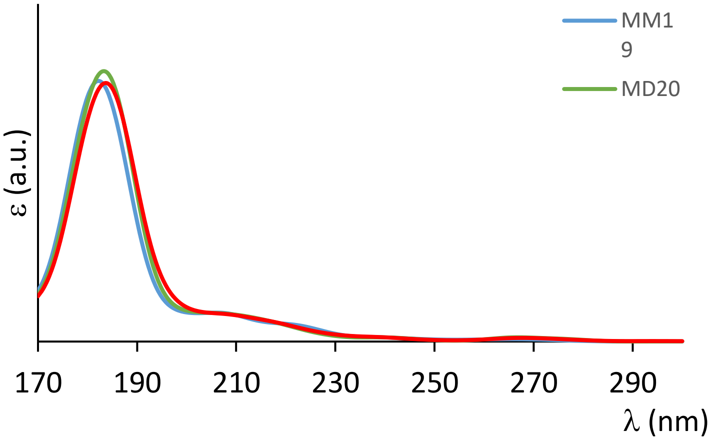

Supplementary Fig. 18. Simulated UV-Vis absorption spectra of **(*S*,*R*)-APnol**

The CD and UV-Vis spectra of **(*S*,*R*)-APnol** modelled from three sets of conformers show that the method to obtain them and weigh their contribution only influences the intensity of the CD spectra, not the sign of the bands. The CD spectra are mainly composed of an intense bisignated CD signal with a positive band at 190 nm and a negative one at 180 nm. A similar positive couplet[19] is observed in the experimental CD spectra of the 1st and 2nd stereoisomers of **APnol** eluted from ChiralPak IG (**Supplementary Fig. 13**), the former being formed as the main stereoisomer when the catalysis is performed with **(*S*)-DTBM SEGPHOS** (**Supplementary Fig. 16b**).

To evaluate the influence of the chirality of **APnol** on the CD and UV-Vis absorption spectra, we selected the conformer of **(*S*,*R*)-APnol** most present in MD (12.5% of the time), built its stereoisomers, optimized their geometry by MM, and modelled their spectra. The *S*,*R* and *R*,*S* conformers are more stable than the *R*,*R* and *S*,*S* conformers by 0.6 kcal/mol, implying that for the *R*,*R* and *S*,*S* stereoisomers these conformers are certainly present in different proportions in their population. However, the computed CD spectra for the different **APnol** stereoisomers clearly reveal that the sign of the different CD bands is dictated by the configuration of the stereogenic center in α position of the amine, whilst the configuration of the stereogenic center in α position of the hydroxy group only affects the intensity of these bands. Upon considering only the two highest energy bands, the (*S*,*R*) and (*R*,*R*) stereoisomers of **APnol** exhibit a positive couplet whilst the (*S*,*S*) and (*R*,*S*) stereoisomers exhibit a negative one.

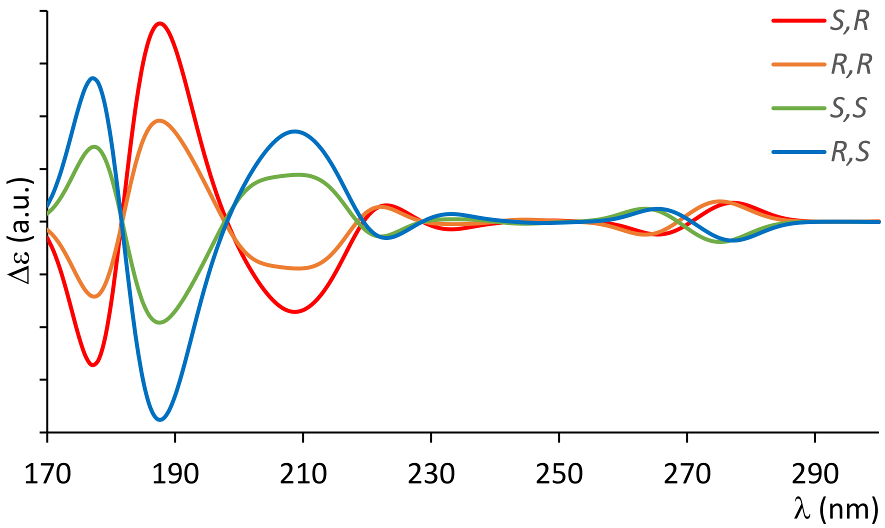

Supplementary Fig. 19. Simulated CD spectra of all the stereoisomers of **APnol**

These calculations allow to ascertain the absolute stereochemistry at the stereogenic centre in α position of the amine function for the four stereoisomers but not that of the stereogenic centre next to the hydroxy group because the experimental CD spectra of the corresponding stereoisomers are virtually identical (**Supplementary Fig. 13**). Considering the intrinsic selectivity of **(*S*)-DTBM SEGPHOS** (which provides the (*S*)-enantiomer of acetophenol in the hydrosilylation of acetophenone),[2] the second eluted stereoisomer from ChiralPak IG is assigned to **(*S*,*R*)-APnol**. The absolute and relative configurations are thus deduced from the experimental and modelled CD spectra as well as optical rotations measured from the isolated stereoisomers of **APnol** yielding the following assignment: **(*R*,*R*)-APnol** (1st peak); **(*S*,*R*)-APnol** (2nd peak); **(*S*,*S*)-APnol** (3rd peak); and **(*R*,*S*)-APnol** (4th peak).

This combined experimental/modelling study does not only establish the absolute and relative configurations of **APnol** but also further supports that the ketone and vinyl functions of **VPnone** are transformed by the Cu-H catalyst coordinated to **DTBM SEGPHOS** in an independent manner.

Determination of the ee, dr, ee1 and ee2 from the HPLC traces (Supplementary Fig. 20)

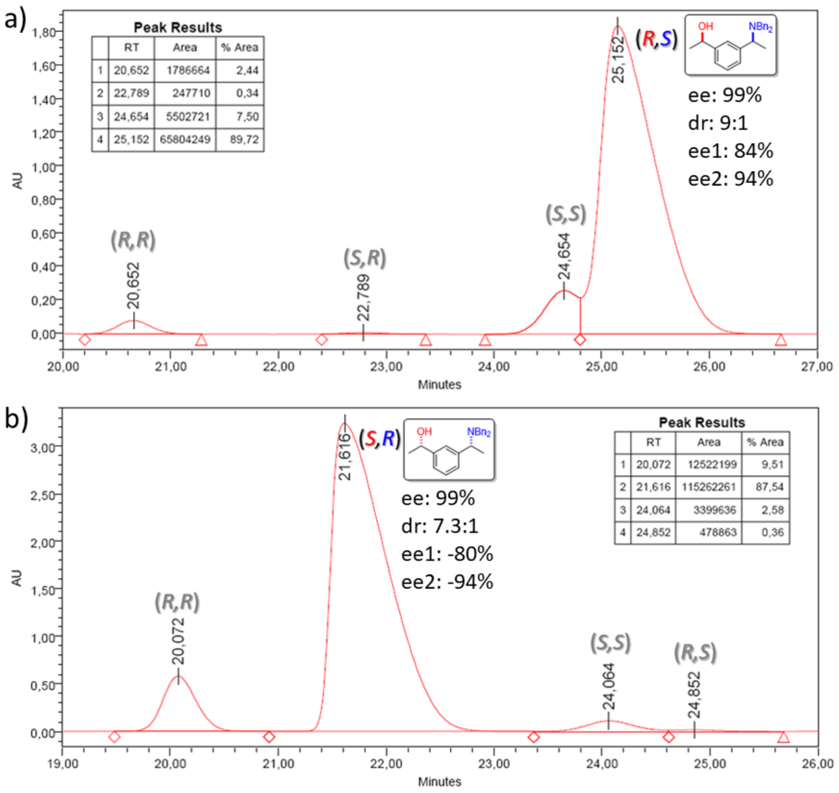

c)

$$ee1 = \frac{(R,S)+(R,R)-(S,R)-(S,S)}{(R,S)+(R,R)+(S,R)+(S,S)}\,;\; ee2 = \frac{(R,S)+(S,S)-(S,R)-(R,R)}{(R,S)+(S,S)+(S,R)+(R,R)}$$

$$ee = \left|\frac{(R,S)-(S,R)}{(R,S)+(S,R)}\right| \;or\; \left|\frac{(S,S)-(R,R)}{(S,S)+(R,R)}\right|;\; ee = \frac{|ee1| + |ee2|}{1 + (|ee1| \times |ee2|)}$$

$$dr = \frac{(S,S)+(R,R)}{(S,R)+(R,S)} \;or\; \frac{(S,R)+(R,S)}{(S,S)+(R,R)};\; dr = \frac{1 + (|ee1| \times |ee2|)}{1 - (|ee1| \times |ee2|)}$$

Supplementary Fig. 20. Determination of the selectivity of the reaction. Chiral HPLC traces of **APnol** isolated from reaction catalyzed by (a) **(*R*)-DTBM-SEGPHOS** and (b) **(*S*)-DTBM-SEGPHOS** (also shown in Supplementary Fig. 16a-b). (c) Ee1, ee2 and ee are extracted from the HPLC traces as indicated in the formulas. Ee and dr can be expressed as a function of ee1 and ee2 as indicated. Ee1 corresponds to the ee of the HS step and is set as positive for the (*R*)-enantiomer of the alcohol. Ee2 corresponds to the ee of the HA step and is set as positive for the (*S*)-enantiomer of the amine.

Note: For all catalytic reactions performed throughout this paper, the ee in **APnol** is related to the ee of the HS and HA steps according to the formula indicated above and thus benefits from the statistical enhancement of the enantiomeric excess as illustrated by the ee in **APnol** being significantly higher than those of the independent steps (compare the value of ee versus ee1 and ee2 in the catalytic tables).

Selected ^{1}H NMR spectra (Supplementary Figs. 21-24)

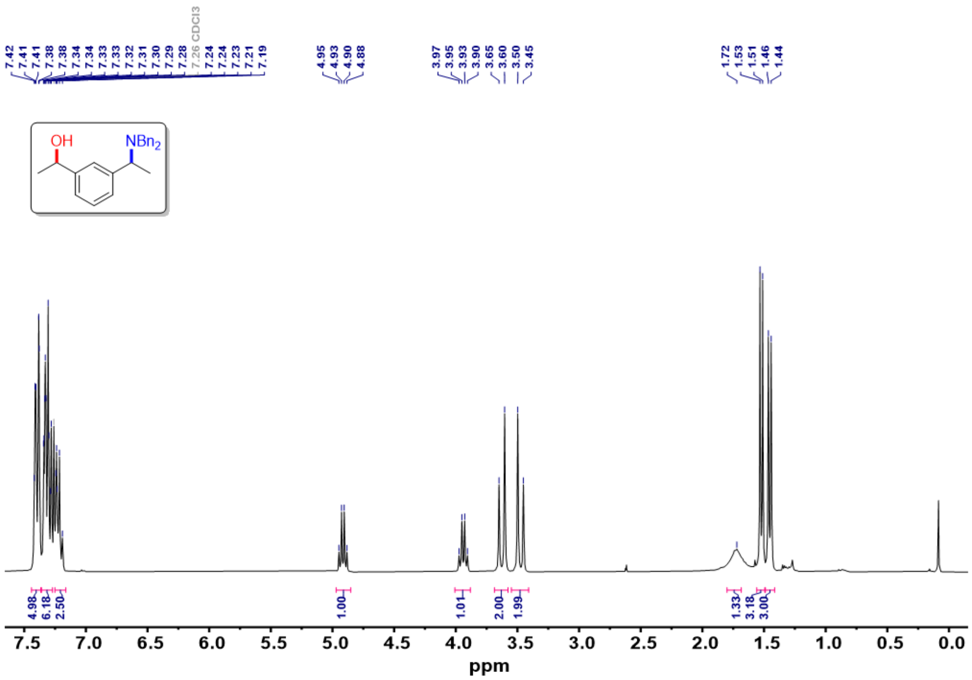

Supplementary Fig. 21. ^{1}H NMR spectrum of isolated **APnol** (300 MHz, $CDCl_3$, 300 K). Reaction catalyzed by **(*R*)-DTBM-SEGPHOS**. For the HPLC trace, see Supplementary Fig. 16a.

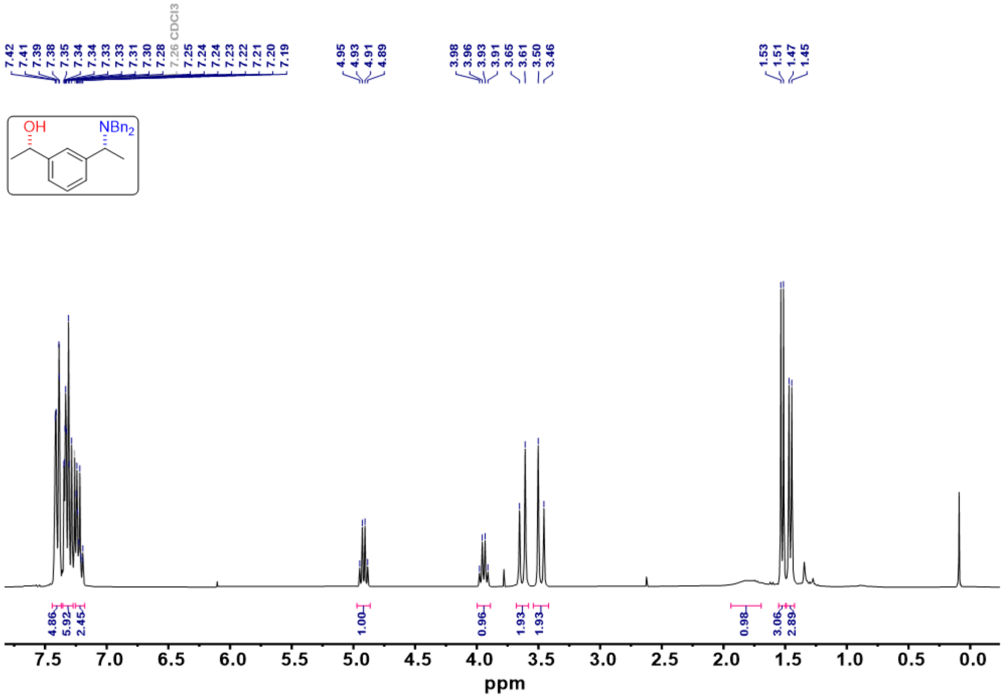

Supplementary Fig. 22. ^{1}H NMR spectrum of isolated **APnol** (300 MHz, $CDCl_3$, 300 K). Reaction catalyzed by **(*S*)-DTBM-SEGPHOS**. For the HPLC trace, see Supplementary Fig. 16b.

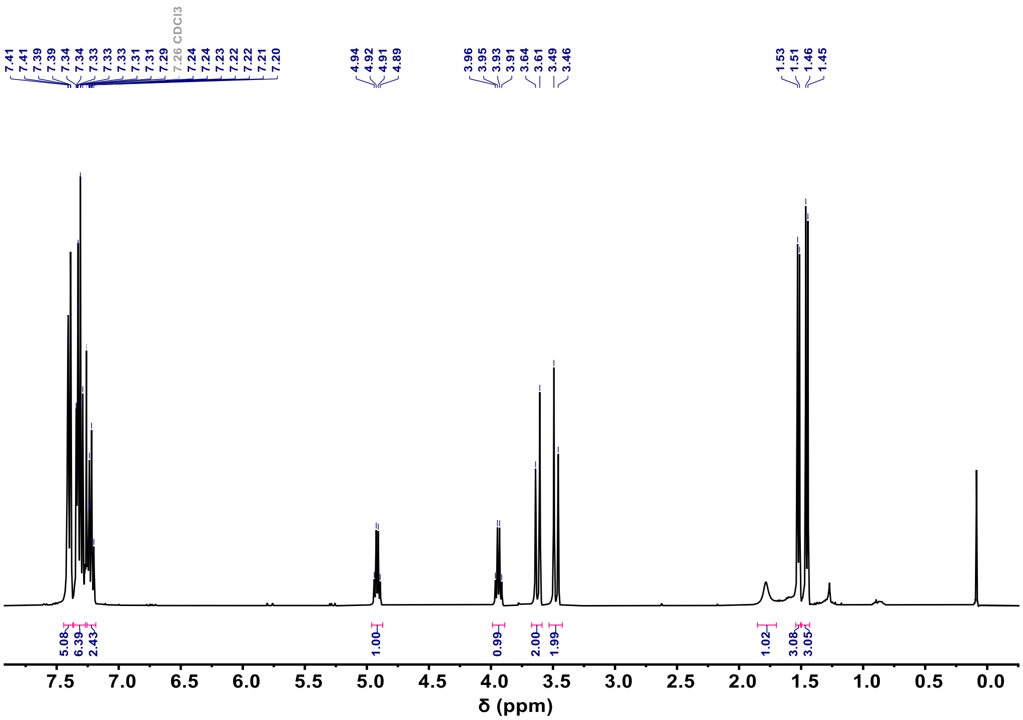

Supplementary Fig. 23. ^{1}H NMR spectrum of isolated **APnol** (300 MHz, $CDCl_3$, 300 K). Reaction catalyzed by BTA helical catalyst: 5 mol% **(*S*)-BTA**, 5 mol% **a-BTA**, 5 mol% P(3,5-$(CF_3)_2$-$C_6H_3)_3$, then 15 mol% **(*R*)-BTA**, 1 mmol scale, reaction under N_2, **Table 2, entry 5**.

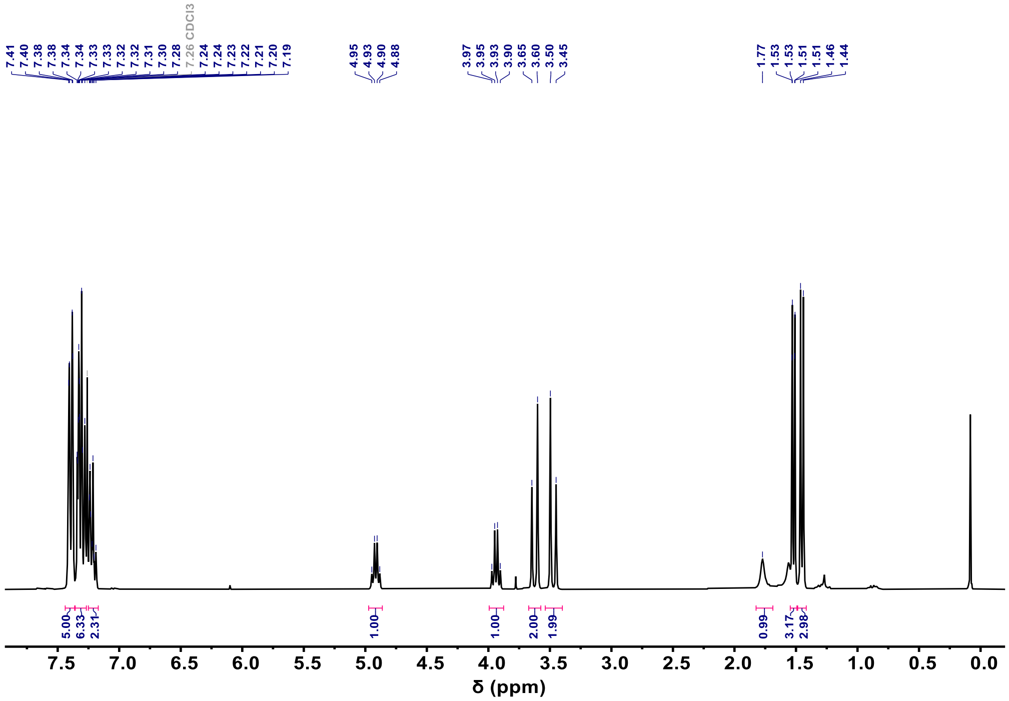

Supplementary Fig. 24. ^{1}H NMR spectrum of isolated **APnol** (300 MHz, $CDCl_3$, 300 K). Reaction catalyzed by BTA helical catalyst: 5 mol% **(*R*)-BTA**, 5 mol% **a-BTA**, 5 mol% P(3,5-$(CF_3)_2$-$C_6H_3)_3$, then 15 mol% **(*S*)-BTA**, 1 mmol scale, reaction under N_2, **Table 2, entry 6**.

Selected HPLC traces (Supplementary Figs. 25-40)

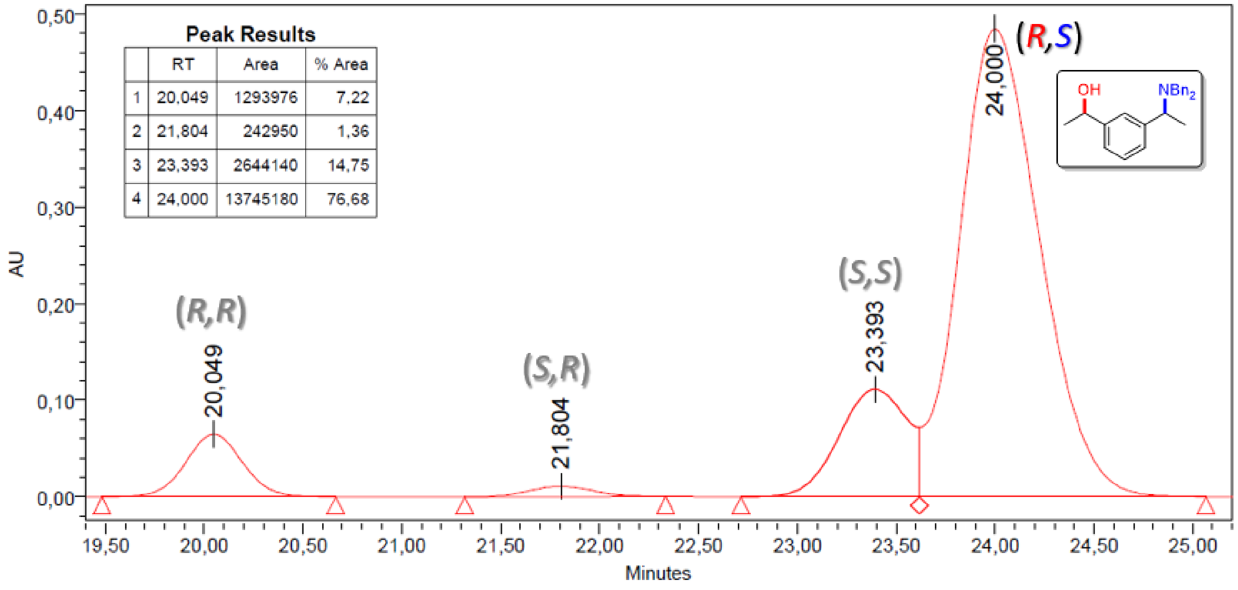

Supplementary Fig. 25. HPLC trace of **APnol**, crude sample. Reaction catalyzed by BTA helical catalyst: 5 mol% **(*S*)-BTA**, 10 mol% **a-BTA**, **Supplementary Table 1, entry 1**. See the corresponding ^{1}H NMR spectrum of in **Supplementary Fig. 3**.

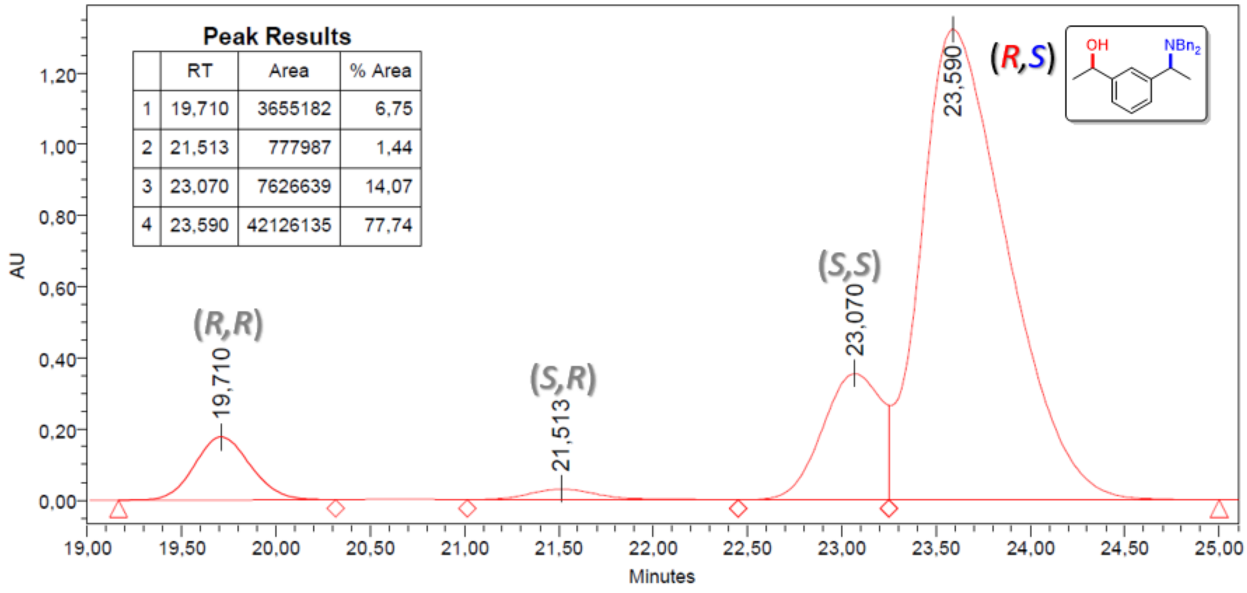

Supplementary Fig. 26. HPLC trace of **APnol**, crude sample. Reaction catalyzed by BTA helical catalyst: 5 mol% **(*S*)-BTA**, 10 mol% **a-BTA**, 5 mol% **P(3,5-(CF_3)$_2$-C_6H_3)$_3$**, **Table 1, entry 1** and **Supplementary Table 1, entry 8**.

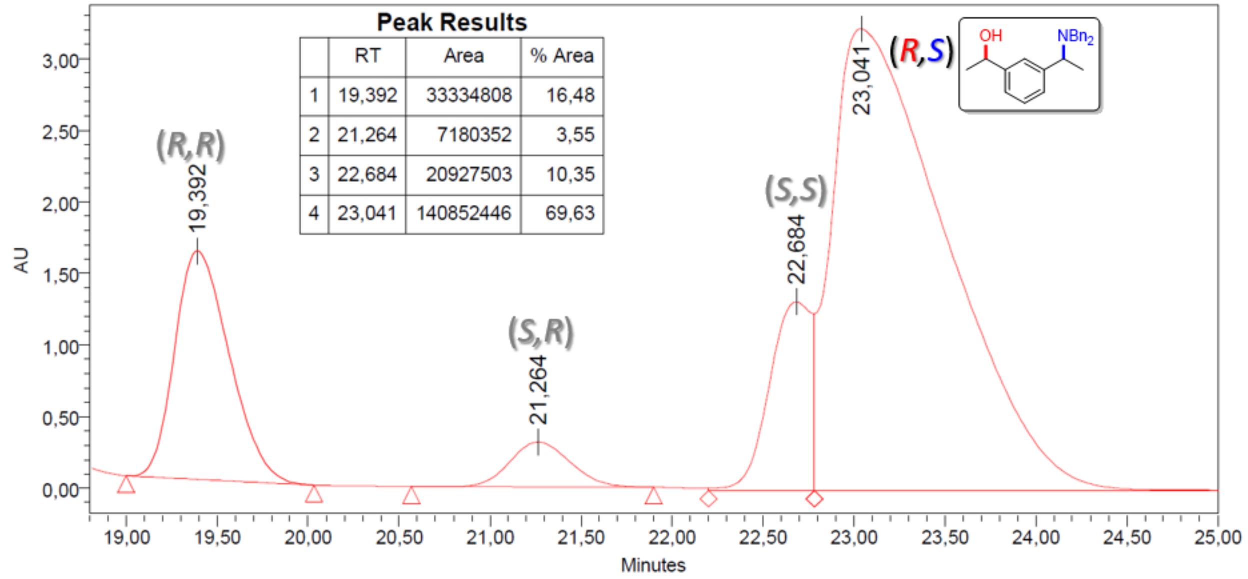

Supplementary Fig. 27. HPLC trace of **APnol**, crude sample. Reaction catalyzed by BTA helical catalyst: 5 mol% **(*S*)-BTA**, no **a-BTA**, 5 mol% **P(3,5-(CF_3)$_2$-C_6H_3)$_3$**, **Supplementary Table 1, entry 14**.

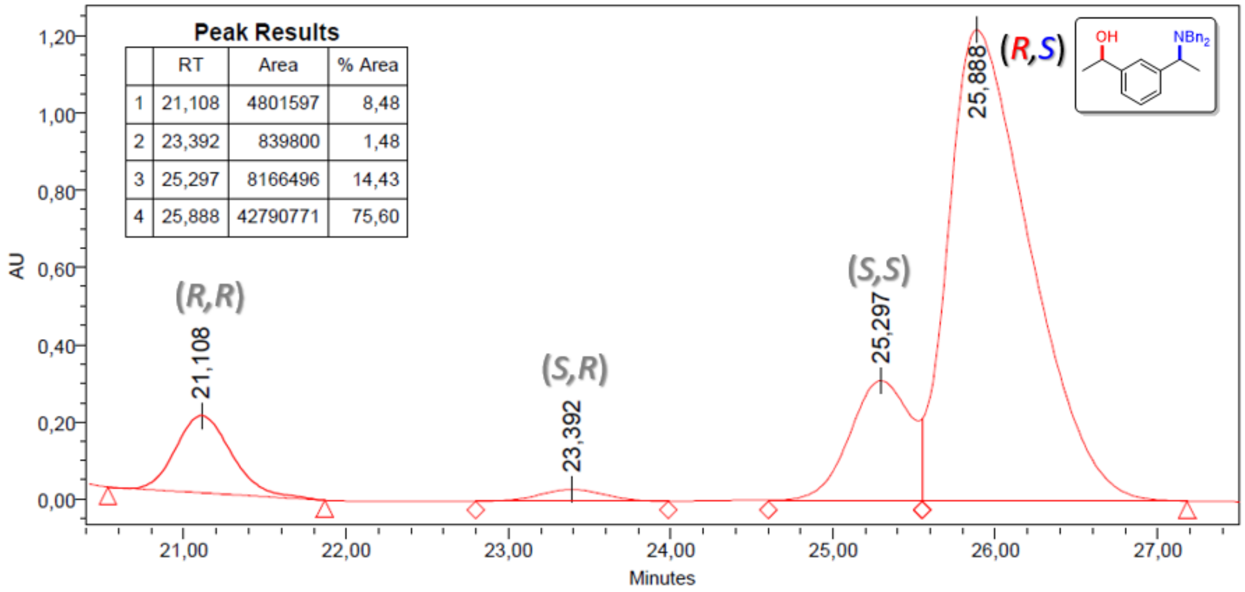

Supplementary Fig. 28. HPLC trace of **APnol**, crude sample. Reaction catalyzed by BTA helical catalyst: 5 mol% **(*S*)-BTA**, 5 mol% **a-BTA**, 5 mol% **P(3,5-(CF_3)$_2$-C_6H_3)$_3$**, **Supplementary Table 1, entry 15**.

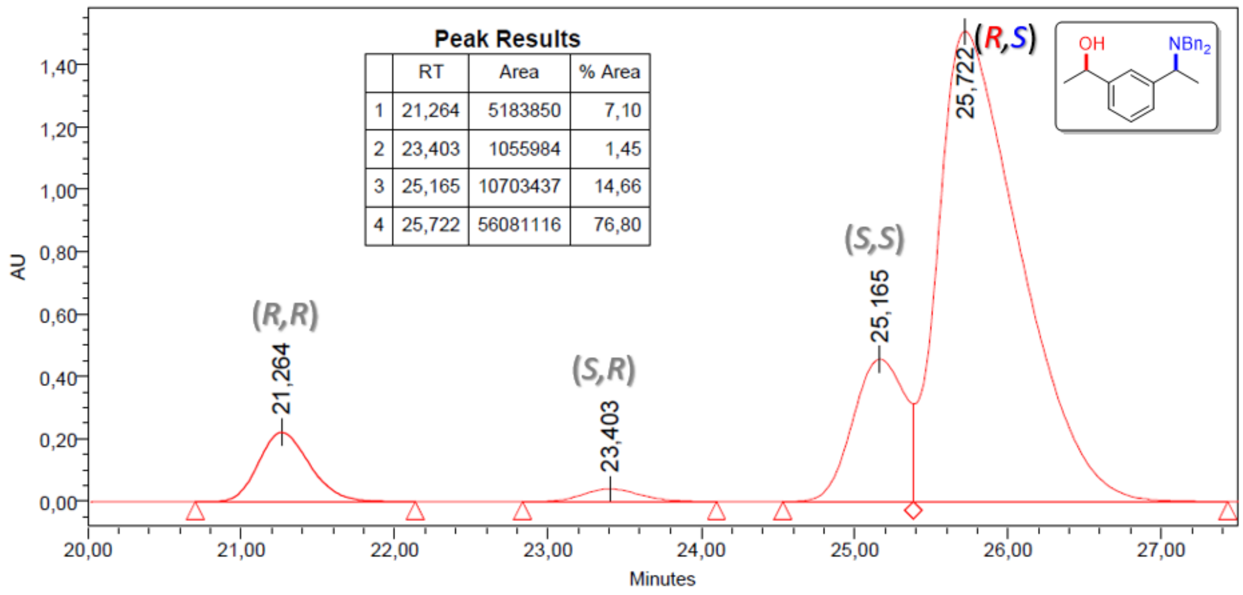

Supplementary Fig. 29. HPLC trace of isolated **APnol**. Reaction catalyzed by BTA helical catalyst: 5 mol% **(*S*)-BTA**, 10 mol% **a-BTA**, 5 mol% **P(3,5-(CF_3)$_2$-C_6H_3)$_3$**, 1 mmol scale, **Supplementary Table 3, entry 1 (also in Fig. 3)**.

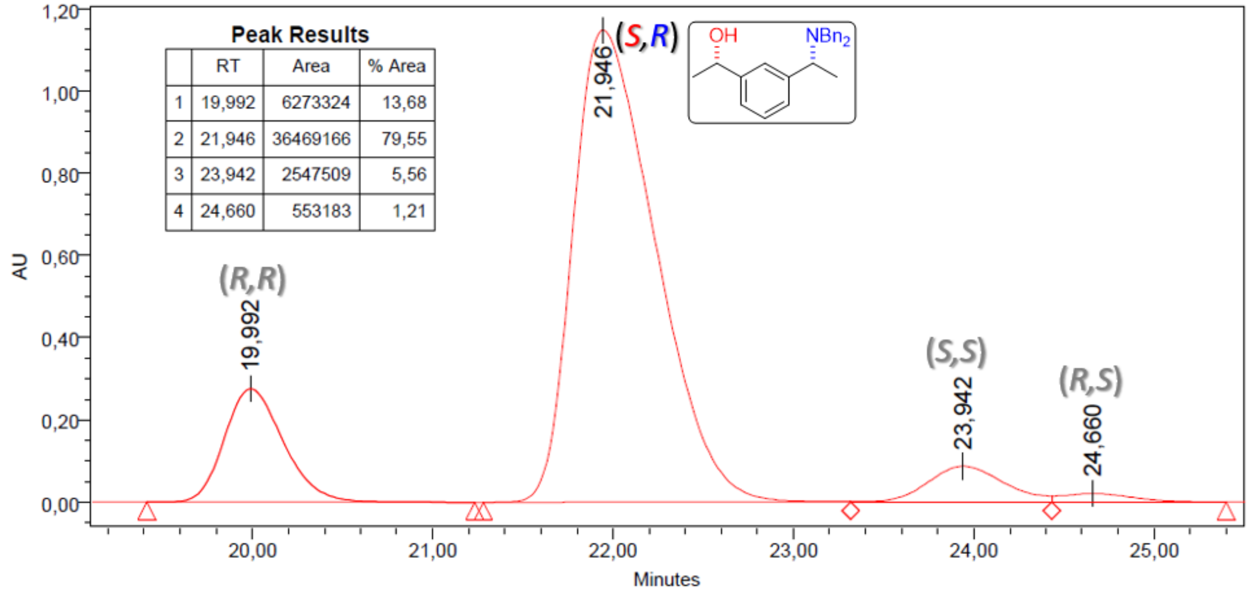

Supplementary Fig. 30. HPLC trace of isolated **APnol**. Reaction catalyzed by BTA helical catalyst: 5 mol% **(*R*)-BTA**, 10 mol% **a-BTA**, 5 mol% **P(3,5-(CF_3)$_2$-C_6H_3)$_3$**, 1 mmol scale, **Supplementary Table 3, entry 2 (also in Fig. 3)**.

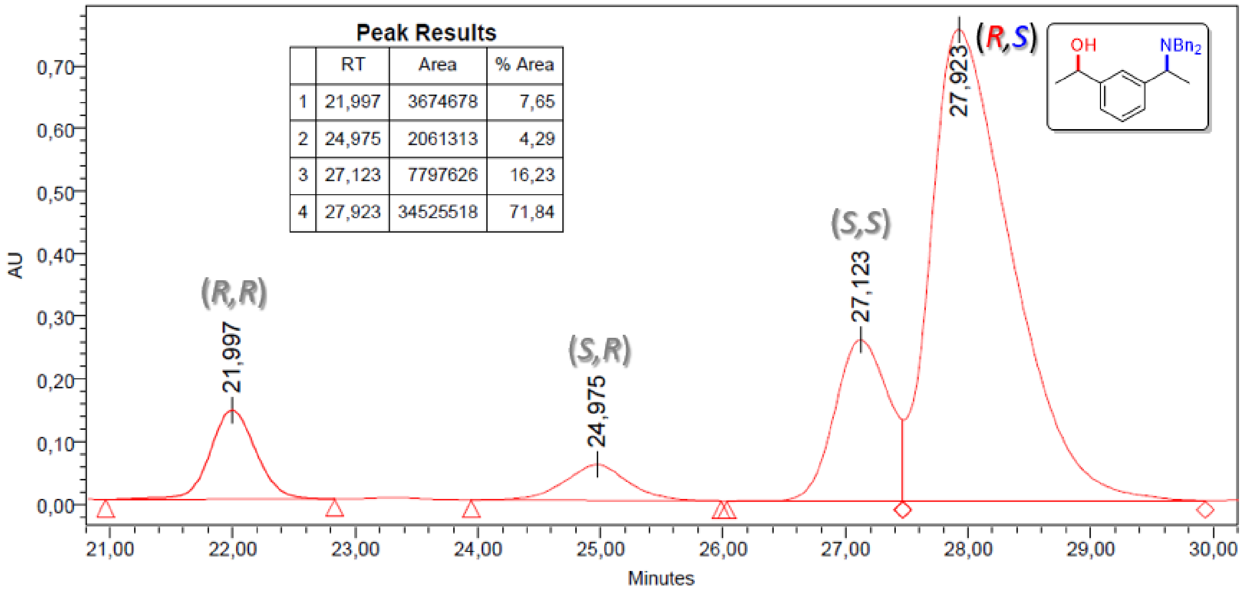

Supplementary Fig. 31. HPLC trace of **APnol**, crude sample. Reaction catalyzed by BTA helical catalyst: 5 mol% **(*S*)-BTA**, 10 mol% **a-BTA**, 5 mol% **P(3,5-(CF_3)$_2$-C_6H_3)$_3$**, with a sequential HS and HA transformations but no switch of the catalyst handedness, **Supplementary Fig. 7**.

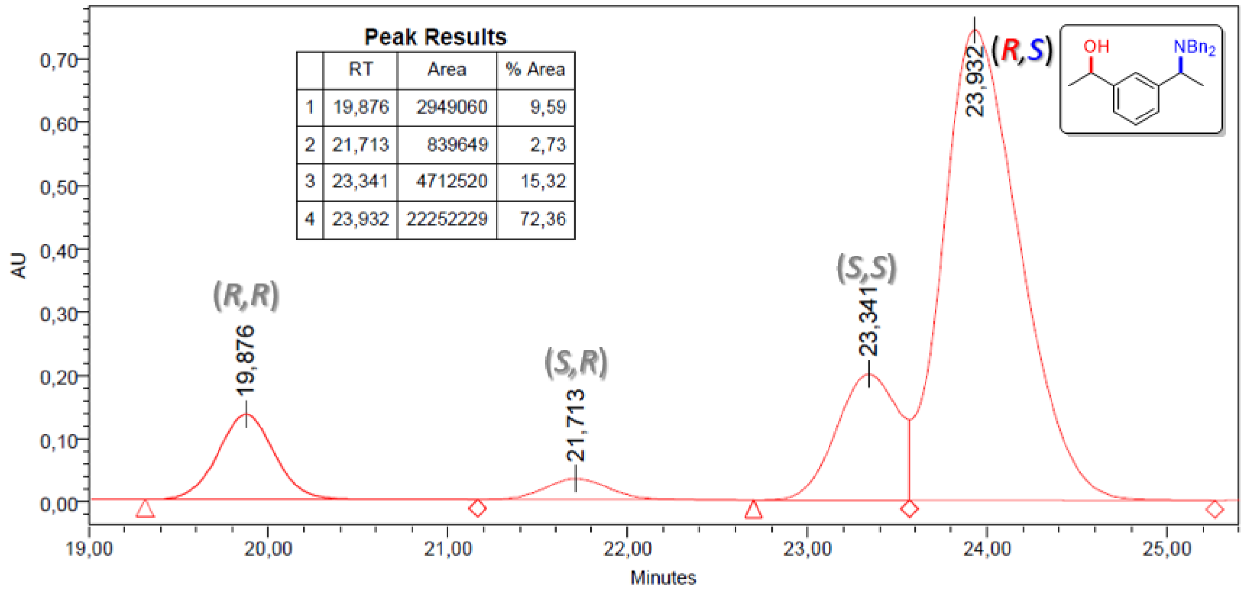

Supplementary Fig. 32. HPLC trace of **APnol**, crude sample. Reaction catalyzed by BTA helical catalyst: 5 mol% **(*S*)-BTA**, 15 mol% **(*R*)-BTA**, 10 mol% **a-BTA**, 5 mol% **P(3,5-(CF_3)$_2$-C_6H_3)$_3$**, with a sequential HS and HA transformations but no switch of the catalyst handedness, **Supplementary Fig. 7**.

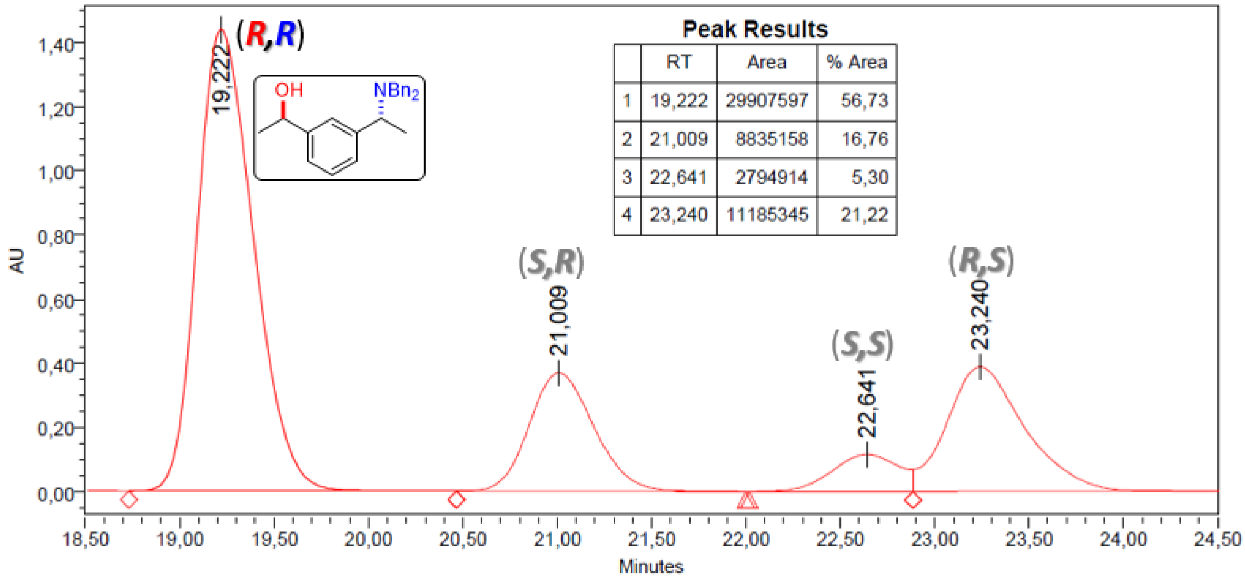

Supplementary Fig. 33. HPLC trace of **APnol**, crude sample. Reaction catalyzed by BTA helical catalyst: 5 mol% **(*S*)-BTA**, 10 mol% **a-BTA**, 5 mol% **P(3,5-(CF_3)$_2$-C_6H_3)$_3$**, then 15 mol% **(*R*)-BTA**, **Table 2, entry 1**.

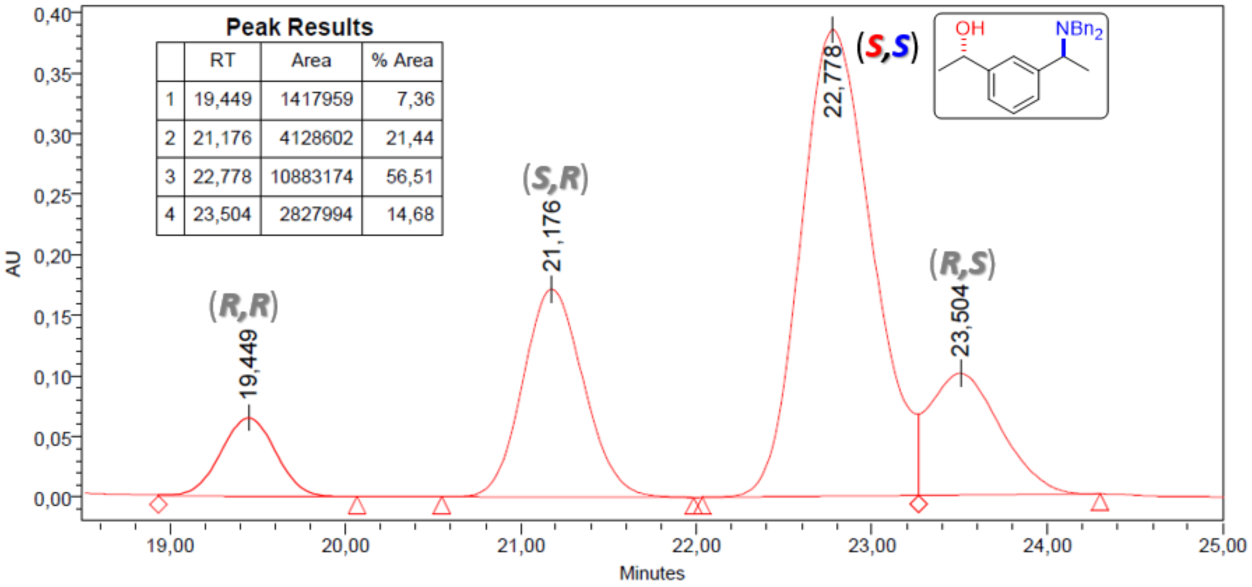

Supplementary Fig. 34. HPLC trace of **APnol**, crude sample. Reaction catalyzed by BTA helical catalyst: 5 mol% **(*R*)-BTA**, 10 mol% **a-BTA**, 5 mol% **P(3,5-(CF_3)$_2$-C_6H_3)$_3$**, then 15 mol% **(*S*)-BTA**, **Table 2, entry 2**.

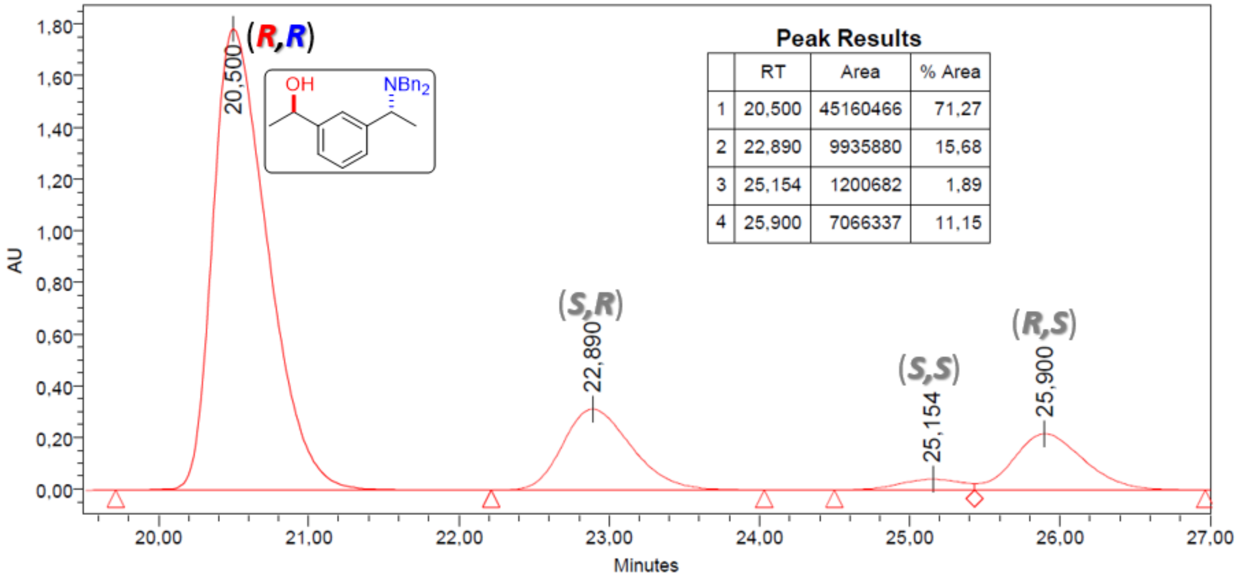

Supplementary Fig. 35. HPLC trace of **APnol**, crude sample. Reaction catalyzed by BTA helical catalyst: 5 mol% **(*S*)-BTA**, 5 mol% **a-BTA**, 5 mol% **P(3,5-(CF$_3$)$_2$-C$_6$H$_3$)$_3$**, then 15 mol% **(*R*)-BTA**, **Table 2, entry 3 (also in Fig. 3)**. See the corresponding ^{1}H NMR spectrum in **Supplementary Fig. 8a**.

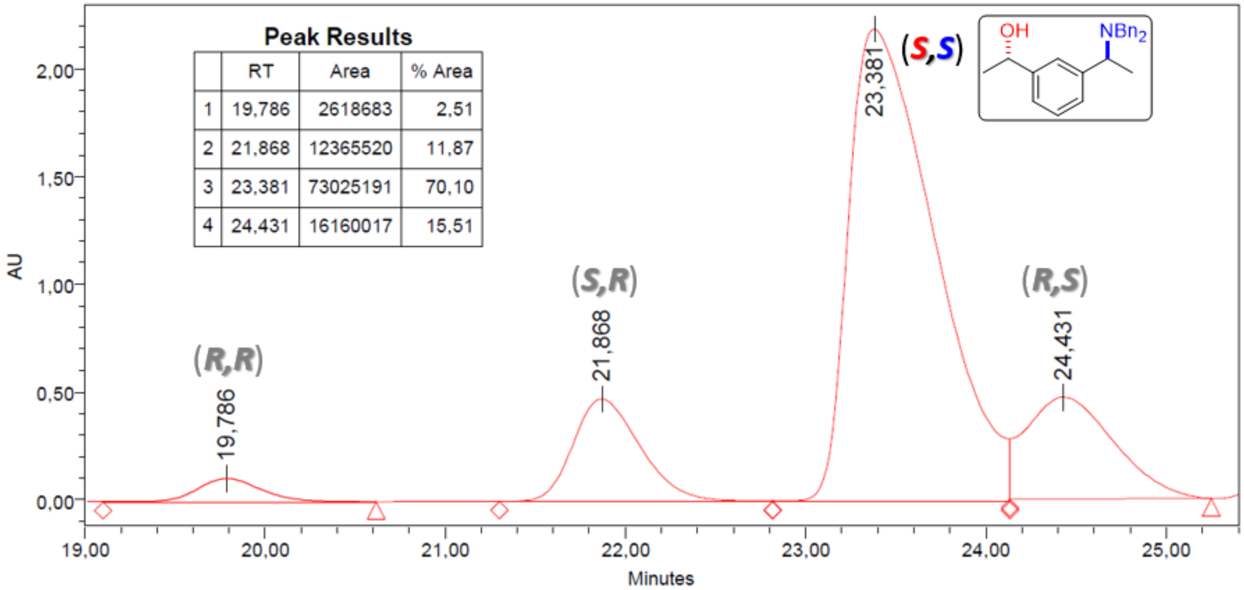

Supplementary Fig. 36. HPLC trace of **APnol**, crude sample. Reaction catalyzed by BTA helical catalyst: 5 mol% **(*R*)-BTA**, 5 mol% **a-BTA**, 5 mol% **P(3,5-(CF$_3$)$_2$-C$_6$H$_3$)$_3$**, then 15 mol% **(*S*)-BTA**, **Table 2, entry 4 (also in Fig. 3)**. See the corresponding ^{1}H NMR spectrum in **Supplementary Fig. 8b**.

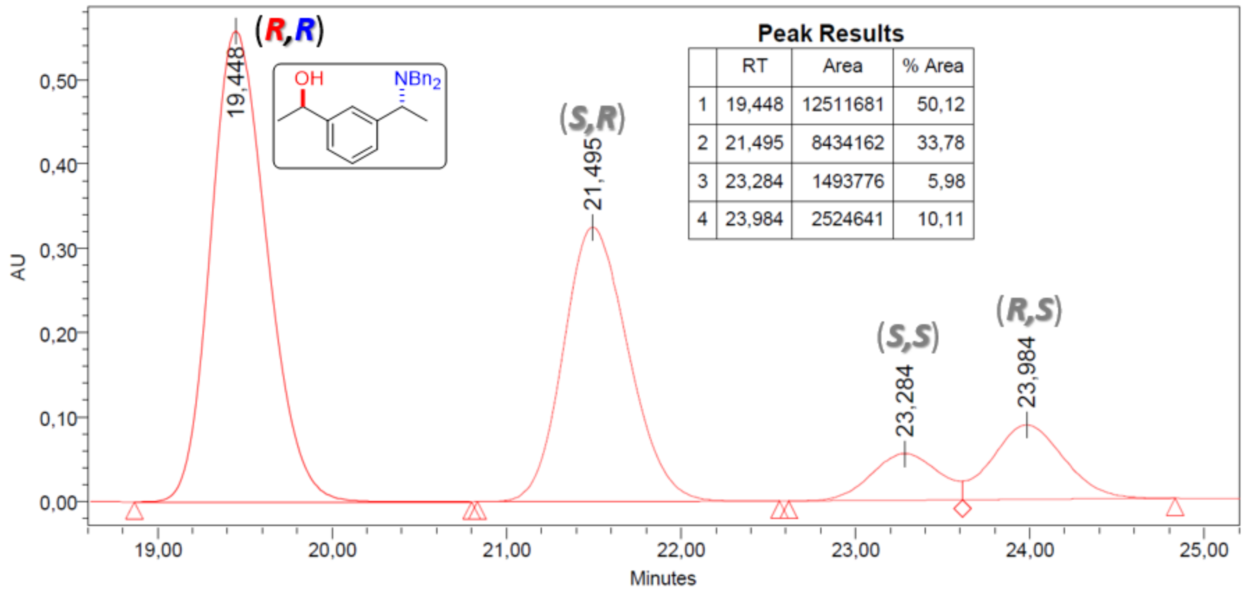

Supplementary Fig. 37. HPLC trace of isolated **APnol**. Reaction catalyzed by BTA helical catalyst: 5 mol% **(*S*)-BTA**, 5 mol% **a-BTA**, 5 mol% **P(3,5-$(CF_3)_2$-C_6H_3)$_3$**, then 15 mol% **(*R*)-BTA**, 1 mmol scale, reaction, under N_2, **Table 2, entry 5**. See the corresponding ^{1}H NMR spectrum in **Supplementary Fig. 9a**.

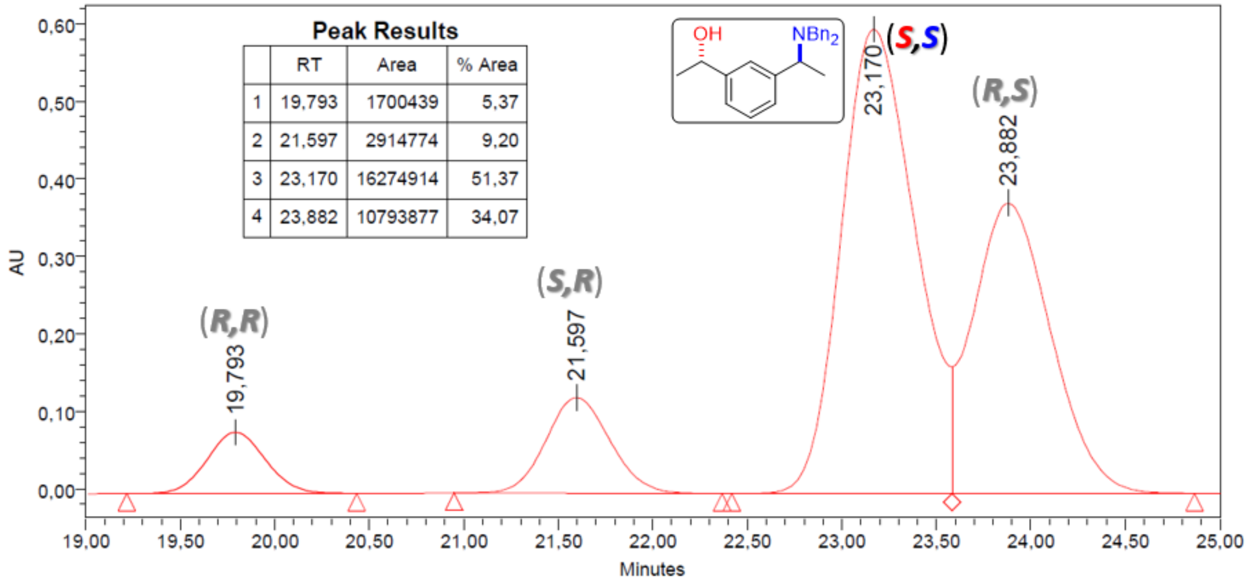

Supplementary Fig. 38. HPLC trace of isolated **APnol**. Reaction catalyzed by BTA helical catalyst: 5 mol% **(*R*)-BTA**, 5 mol% **a-BTA**, 5 mol% **P(3,5-$(CF_3)_2$-C_6H_3)$_3$**, then 15 mol% **(*S*)-BTA**, 1 mmol scale, reaction under N_2, **Table 2, entry 6**. See the corresponding ^{1}H NMR spectrum in **Supplementary Fig. 9b**.

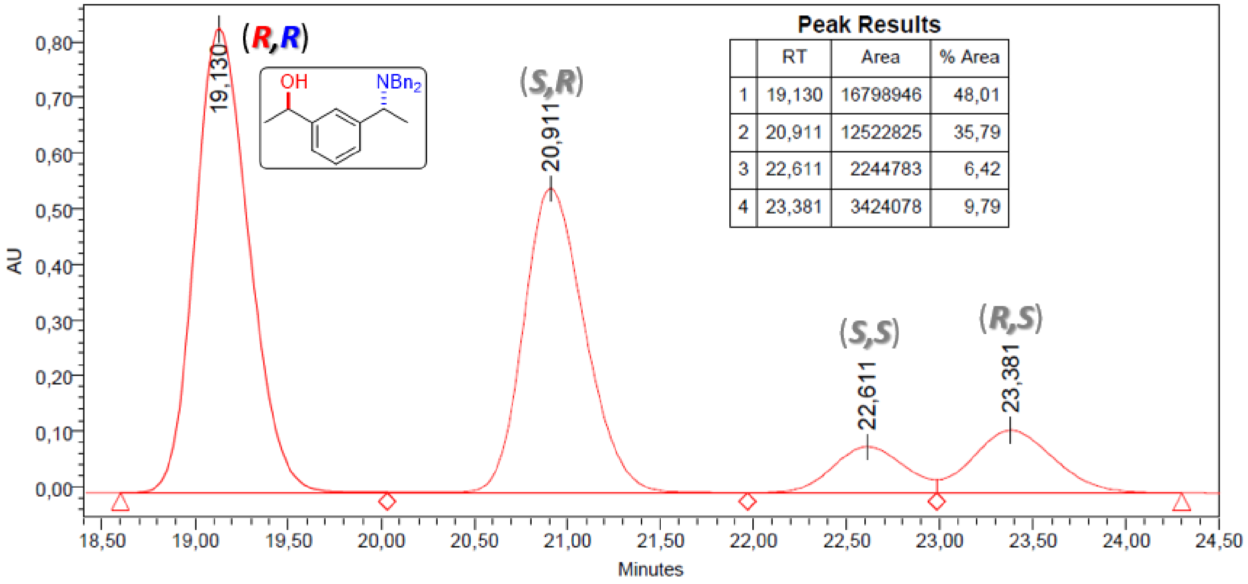

Supplementary Fig. 39. HPLC trace of isolated **APnol**. Reaction catalyzed by BTA helical catalyst: 5 mol% **(*S*)-BTA**, 5 mol% **a-BTA**, 5 mol% **P(3,5-(CF$_3$)$_2$-C$_6$H$_3$)$_3$**, then 15 mol% **(*R*)-BTA**, 1 mmol scale, reaction under N$_2$, **Supplementary Table 4, entry 3**.

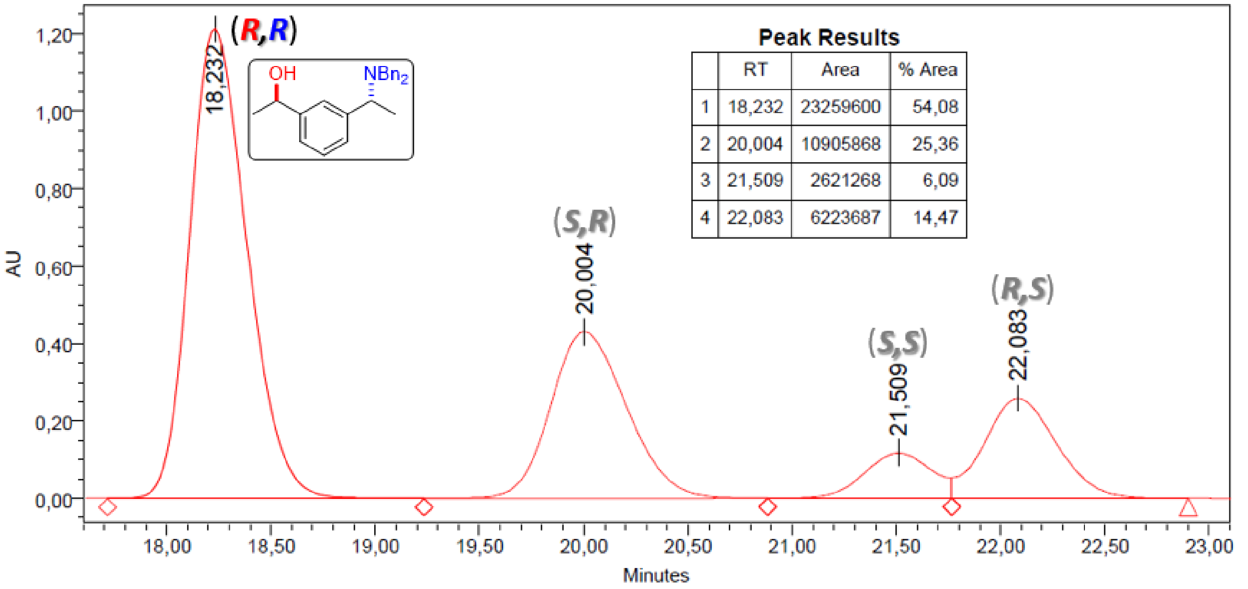

Supplementary Fig. 40. HPLC trace of isolated **APnol**. Reaction catalyzed by BTA helical catalyst: 5 mol% **(*S*)-BTA**, 5 mol% **a-BTA**, 5 mol% **P(3,5-(CF$_3$)$_2$-C$_6$H$_3$)$_3$**, then 15 mol% **(*R*)-BTA**, 1 mmol scale, reaction under N$_2$, **Supplementary Table 4, entry 4**.

Characterization of the catalytic products (Supplementary Figs. 41-46)

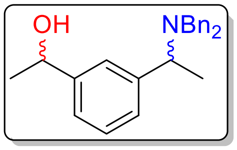

1-[3-(1-dibenzylaminoethyl)]-acetophenol (**APnol**).

The product was isolated from the HS/HA transformation of **VPnone** with the BTA helical catalyst as described in the catalytic procedures (1 mmol scale reaction). Enantiopure stereoisomers of **APnol** were also isolated from preparative chiral HPLC. Analytical data: ^{1}H NMR (300 MHz, $CDCl_3$) δ 7.44 – 7.36 (m, 5H), 7.36 – 7.27 (m, 7H), 7.25 – 7.16 (m, 2H), 4.92 (q, *J* = 6.4 Hz, 1H), 3.94 (q, *J* = 6.9 Hz, 1H), 3.63 (d, *J* = 13.8 Hz, 2H), 3.47 (d, *J* = 13.8 Hz, 2H), 1.72 (br s, 1H), 1.52 (d, *J* = 6.5 Hz, 3H), 1.45 (d, *J* = 6.9 Hz, 3H). Stereoisomers exhibit some different 13C chemical shifts: ^{13}C{^{1}H} NMR (101 MHz, $CDCl_3$) δ 145.57 (Carom., (*S*,*S*)/(*R*,*R*)), 145.53 (Carom., (*S*,*R*)/(*R*,*S*)), 143.38 (Carom., (*S*,*R*)/(*R*,*S*)), 143.33 (Carom., (*S*,*S*)/(*R*,*R*)), 140.55 (C.arom., NBn_2), 128.78 (CHarom., NBn_2), 128.34 (CHarom., NBn_2), 128.27 (CHarom., (*S*,*S*)/(*R*,*R*)), 128.25 (CHarom., (*S*,*R*)/(*R*,*S*)), 127.33 (CHarom., (*S*,*R*)/(*R*,*S*)), 127.30 (CHarom., (*S*,*S*)/(*R*,*R*)), 126.89 (CHarom., NBn_2), 125.27 (CHarom., (*S*,*S*)/(*R*,*R*)), 125.21 (CHarom., (*S*,*R*)/(*R*,*S*)), 124.01 (CHarom., (*S*,*R*)/(*R*,*S*)), 123.87 (CHarom., (*S*,*S*)/(*R*,*R*)), 70.74 (C*H*OH), 56.49 (C*H*NBn_2, (*S*,*R*)/(*R*,*S*)), 56.42 (C*H*NBn_2, (*S*,*S*)/(*R*,*R*)), 53.83 (CH_2, (*S*,*R*)/(*R*,*S*)), 53.81 (CH_2, (*S*,*S*)/(*R*,*R*)), 25.33 (CH_3, next to CHOH, (*S*,*S*)/(*R*,*R*)), 25.28 (CH_3, next to CHOH, (*S*,*R*)/(*R*,*S*)), 13.99 (CH_3, next to $CHNBn_2$, (*S*,*R*)/(*R*,*S*)), 13.91 (CH_3, next to $CHNBn_2$, (*S*,*S*)/(*R*,*R*)). HRMS (ESI, m/z) for $C_{24}H_{28}NO$, $[M+H]^+$: calculated: 346.2165; found: 346.2156. IR (ATR diamond, cm^{-1}): 696 (s), 737 (s), 793 (m), 907 (m), 1009 (m), 1026 (m), 1072 (m), 1126 (m), 1167 (m), 1242 (m), 1315 (m), 1366 (m), 1452 (m), 1493 (m), 1603 (m), 1722 (w), 1807 (w), 1873 (w), 1950 (w), 2800 (br), 2880 (br), 2933 (br), 2968 (m), 3024 (m), 3064 (m), 3346 (br). Optical rotation, CD/UV-Vis absorption spectrum and ^{1}H NMR spectrum for each stereoisomer of **APnol** are reported in **Supplementary Table 5**, **Supplementary Fig. 13** and **Supplementary Fig. 15**, respectively.

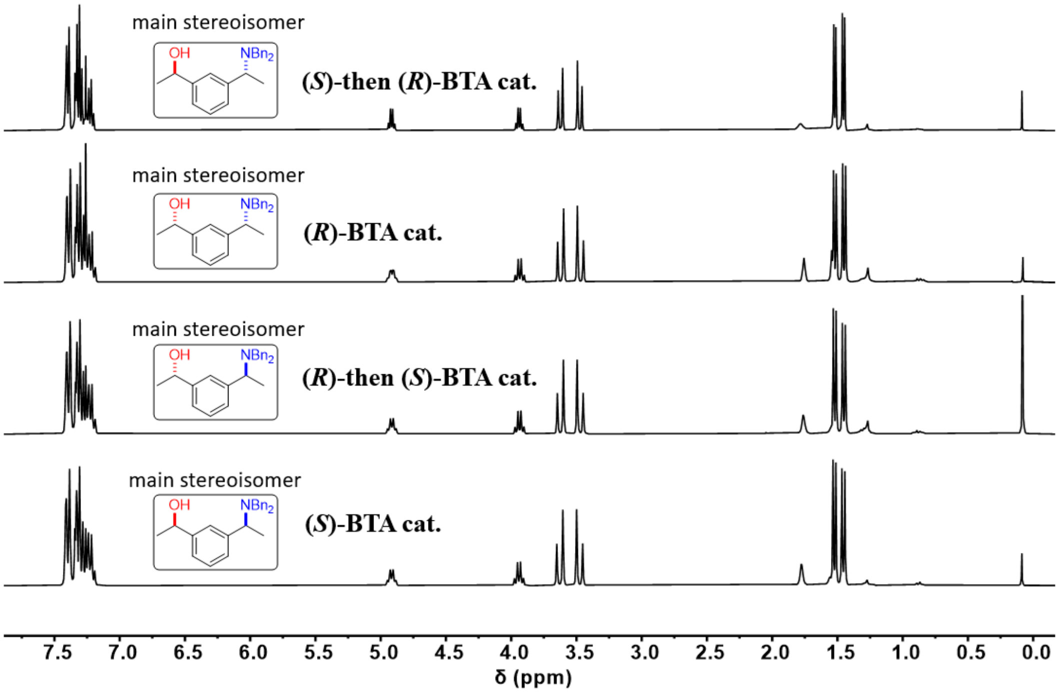

Supplementary Fig. 41. ^{1}H NMR spectra (400 MHz, $CDCl_3$, 300 K) of the stereoisomers of **APnol** isolated from the catalytic experiments performed with BTA helical catalysts (only the main stereoisomer is drawn).

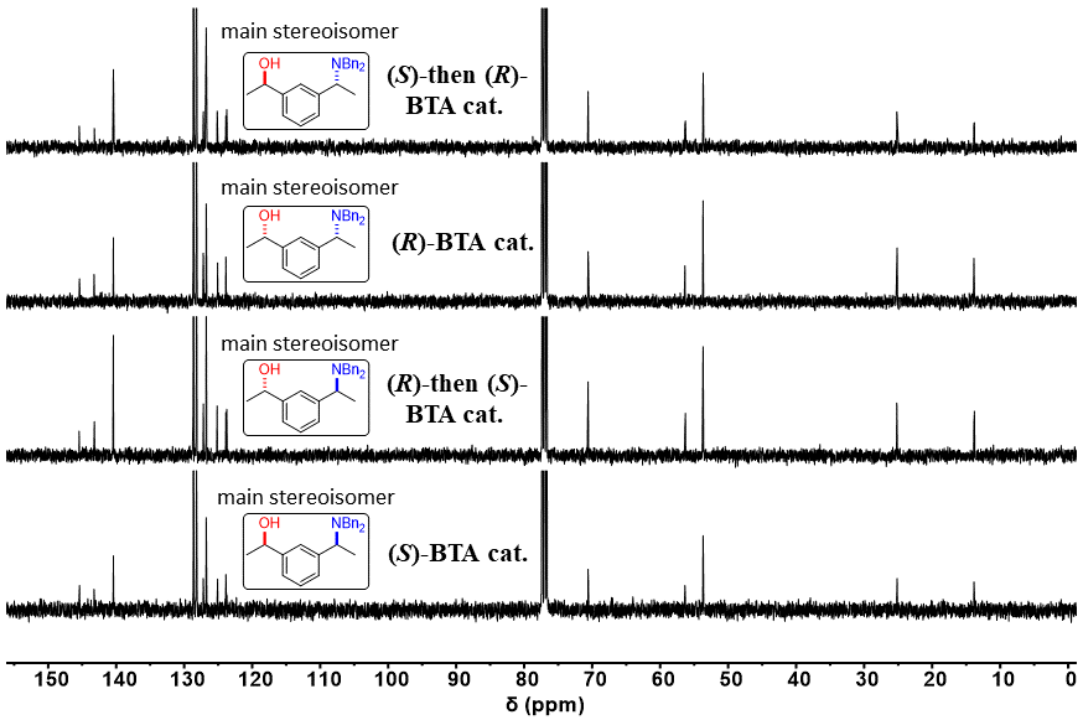

Supplementary Fig. 42. $^{13}C\{^{1}H\}$ NMR spectra (101 MHz, $CDCl_3$, 300 K) of the stereoisomers of **APnol** isolated from the catalytic experiments with BTA helical catalysts (only the main stereoisomer is drawn).

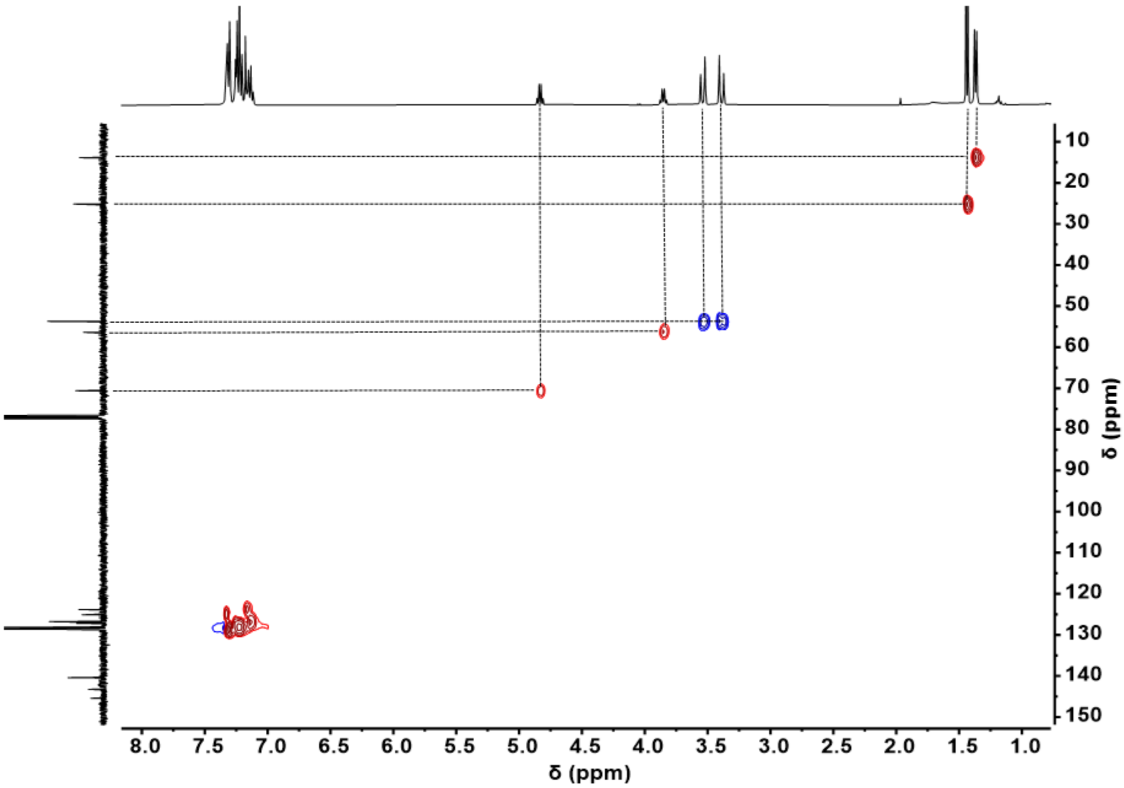

Supplementary Fig. 43. HSQC spectrum of **APnol** isolated from the catalysis performed with the BTA helical catalyst embedding **(*R*)-BTA (Supplementary Table 3, entry 2)**.

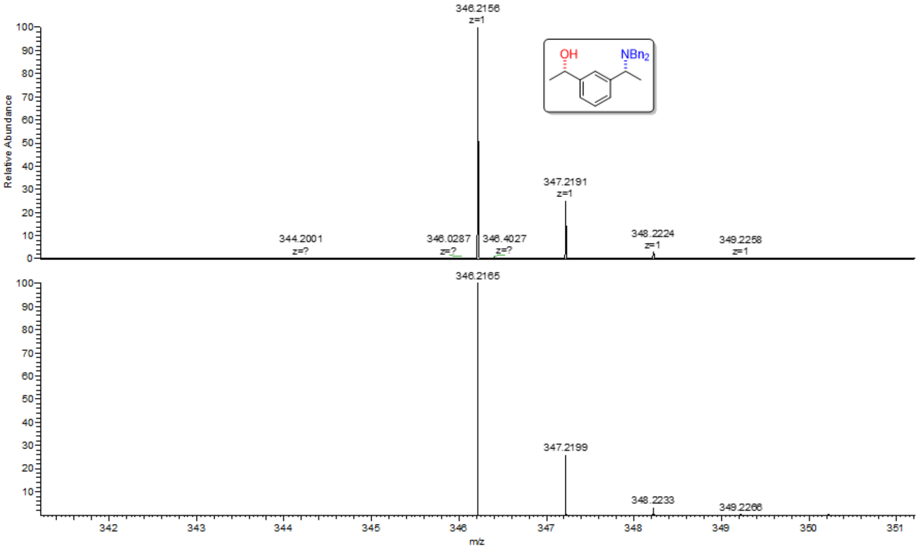

Supplementary Fig. 44. Experimental and simulated isotopic distribution for [**APnol+H**]$^+$.

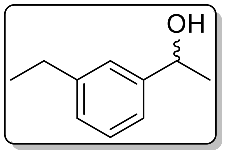

3-ethylacetophenol (**EPnol**).

The product was isolated from the HS/HA transformation of **VPnone** catalyzed by an equimolar mixture of **(*S*)-DTBM SEGPHOS** and **(*R*)-DTBM SEGPHOS**. Purified by flash column chromatography over silica gel, eluting with petroleum ether/dichloromethane (gradient from 100/0 to 30/70). Analytical data are consistent with the literature.[20]

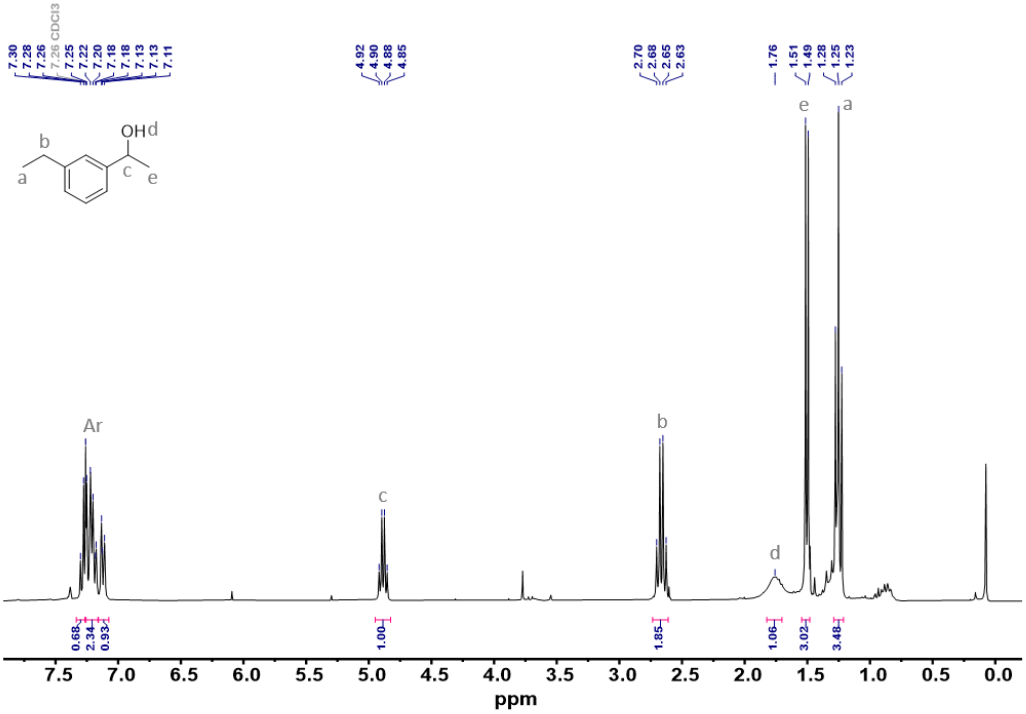

Supplementary Fig. 45. ^{1}H NMR spectrum of **EPnol** (300 MHz, $CDCl_3$, 300 K).

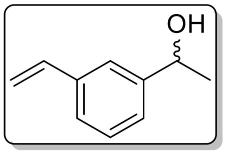

3-vinylacetophenol (**VPnol**).

The product was isolated from the HS of **VPnone** as follows: an oven-dried reaction tube was loaded with **BTA P** (43.4 mg, 45 µmol, 10.0 mol%), $Cu(OAc)_2$ (4.2 mg, 22.5 µmol, 5.0 mol%), $P(3,5\text{-}(CF_3)_2\text{-}C_6H_3)_3$ (15.2 mg, 22.5 µmol, 5 mol%) and anhydrous THF (500 µL), and the mixture was stirred for 10 minutes. The solvent was then removed under vacuum and the tube was kept in vacuum (10^{-3} mbar) for 1 hour. **(*S*)-BTA** (23.8 mg, 22.5 µmol, 5 mol%), **a-BTA** (49.2 mg, 45 µmol, 10.0 mol%), and anhydrous toluene (4 mL) were added to the tube and the mixture was briefly heated to reflux and stirred for 10 minutes at room temperature. **VPnone** (65.8 mg, 450 µmol, 100 mol%) were added to the tube. The reaction mixture was heated to 313 K, DMMS was added to the tube (280 µL, 2.25 mmol, 500 mol%) and the mixture was stirred for 2 hours. The reaction mixture was cooled down to room temperature. A saturated solution of NH_4F (4 mL) in MeOH was added and the mixture was stirred until it became transparent. Then a saturated aqueous solution of Na_2CO_3 (4 mL) as well as EtOAc (2 mL) were added. The phases were separated and the aqueous layer was extracted with ethyl acetate (2×2 mL). The combined organic phases were concentrated in vacuum and purified by flash column chromatography over silica gel, eluting with dichloromethane to yield **VPnol** as a faint yellow oil (42 mg, 62% yield). Analytical data are consistent with the literature.[21]

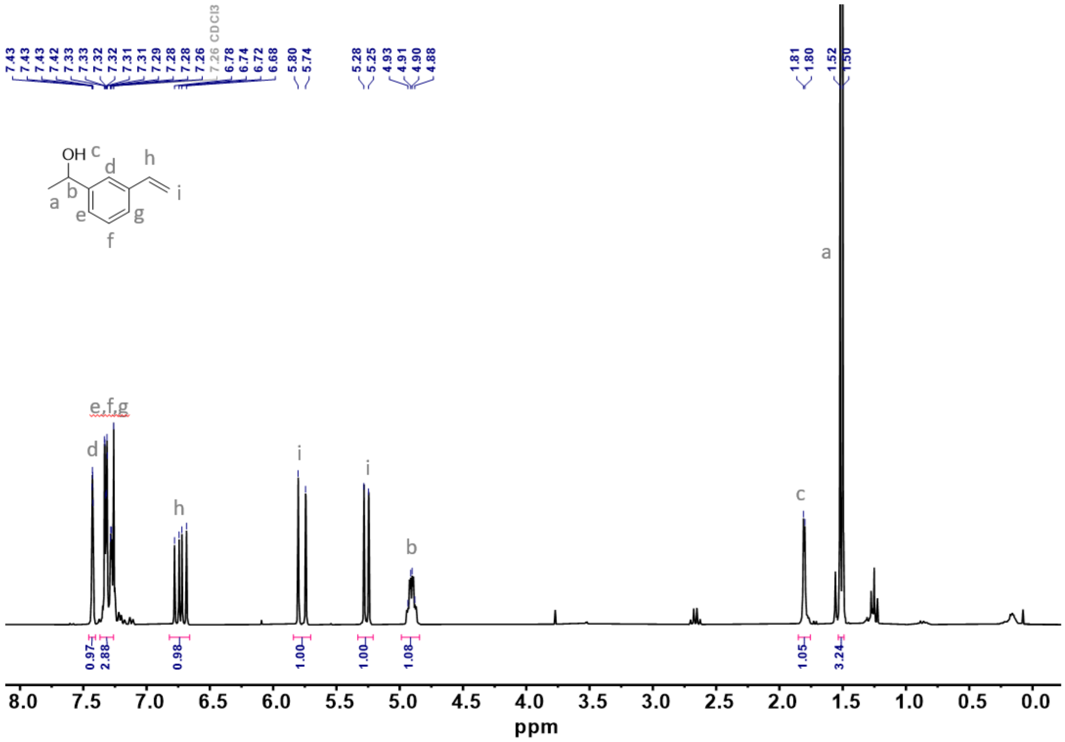

Supplementary Fig. 46. ^{1}H NMR spectrum of **VPnol** (300 MHz, $CDCl_3$, 300 K). Traces of **EPnol** are present in the sample.

Synthesis of 3-vinylacetophenone, VPnone (Supplementary Figs. 47-48)

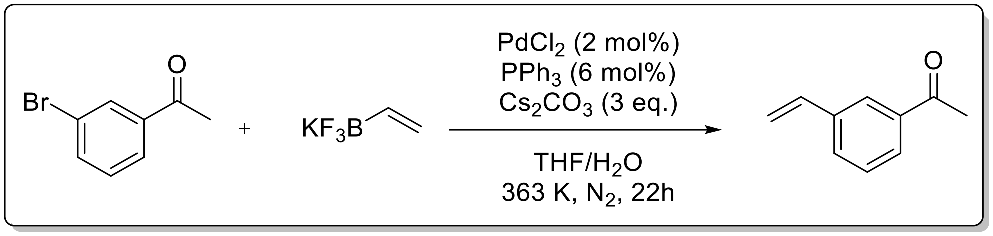

The synthesis was adapted from the reported procedure in order to yield multi-gram amount of **VPnone**.[23]

3'-bromoacetophenone (4.0 g, 1.0 eq, 20 mmol), potassium vinyl trifluoroborate (3.5 g, 1.3 eq, 26 mmol), $PdCl_2$ (71 mg, 0.02 eq, 0.4 mmol), PPh_3 (315 mg, 0.06 eq, 1.2 mmol) and Cs_2CO_3 (19.6 g, 0.02 eq, 60 mmol) were added into a Schlenk tube equipped with a sealing cap, followed by the addition of 100 mL of a THF/H_2O mixture (9/1). The tube was then evacuated and backfilled with N_2 for a total of 3 times. The mixture was stirred at 363 K for 22 hours. After cooling down to room temperature, the mixture was washed with 80 mL of H_2O and extracted with dichloromethane (3×30 mL). The crude product was concentrated under vacuum and purified by flash column chromatography over silica gel eluting with petroleum ether/ethyl acetate 20/1, yielding **VPnone** as a colorless liquid (2.6 g, 89% yield). Analytical data are consistent with the literature.[22] ^{1}H NMR (400 MHz, $CDCl_3$) δ 7.98 (d, *J*= 1.9 Hz, 1H), 7.84 (dt, *J*= 7.7 Hz, 1.5 Hz, 1H), 7.61 (d, *J*= 7.7 Hz, 1H), 7.42 (t, *J*= 7.7 Hz, 1H), 6.76 (dd, *J*= 17.6 Hz, 10.9 Hz, 1H), 5.83 (d, *J*= 16.8 Hz, 1H), 5.34 (d, *J*= 11.5 Hz, 1H), 2.62 (s, 3H). ^{13}C{^{1}H} NMR (101 MHz, $CDCl_3$) δ 198.07, 138.05, 137.45, 135.99, 130.59, 128.79, 127.67, 126.04, 115.28, 26.70. HRMS (ESI, m/z) for $C_{10}H_{11}O$, $[M+H]^+$: calculated: 147.0804; found: 147.0808.

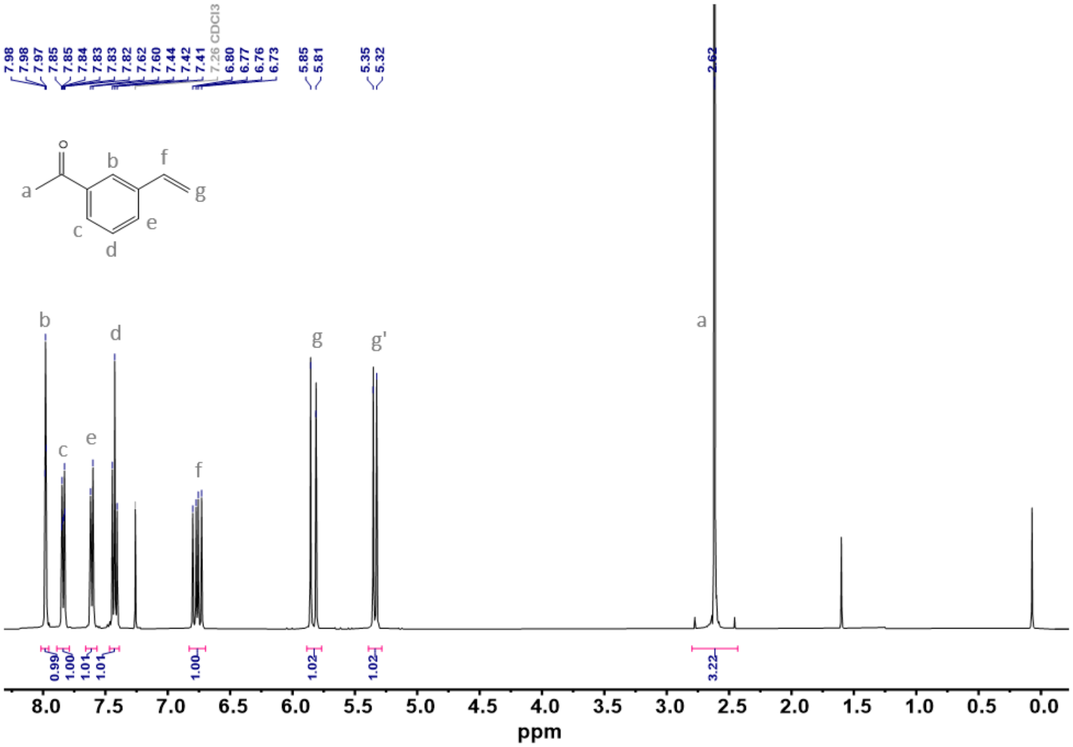

Supplementary Fig. 47. ^{1}H NMR spectrum of **VPnone** (400 MHz, $CDCl_3$, 300 K).

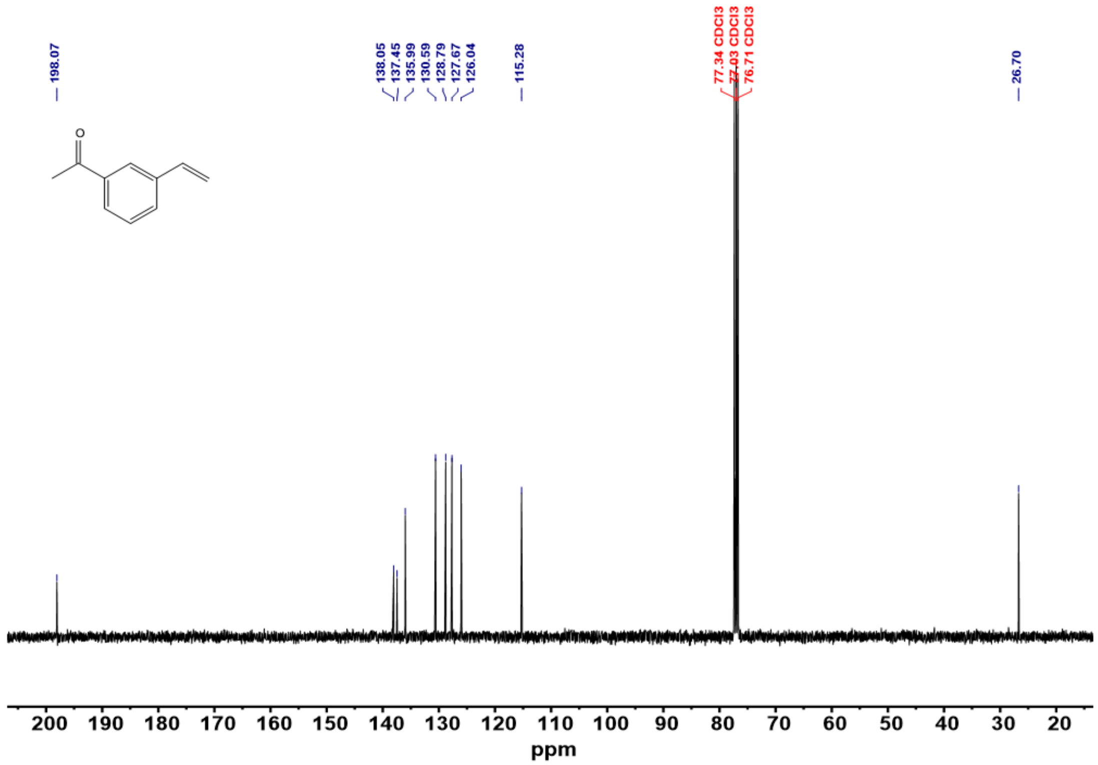

Supplementary Fig. 48. $^{13}C\{^{1}H\}$ NMR spectrum of **VPnone** (101 MHz, $CDCl_3$, 300 K).

Catalytic procedures

Note that the excess of DMMS (an eye-irritant chemical) is quenched with ammonium fluoride to avoid evaporation following the procedure described in reference [13]. The enantiomeric excess (ee) and diastereoisomeric ratio (dr) of **APnol** and the enantiomeric excess of **VPnol** were determined by HPLC analysis of the crude or purified samples on Chiralpak IG.

Catalysis with (*rac*)-DTBM-SEGPHOS, Supplementary Fig. 10:

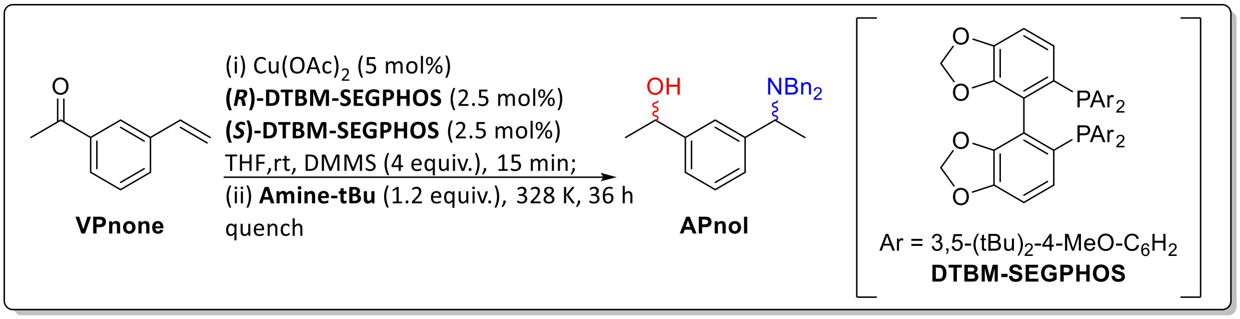

An oven-dried reaction tube was loaded with equimolar mixture of **(*S*)-DTBM-SEGPHOS** and **(*R*)-DTBM-SEGPHOS** (total: 20.4 mg, 0.017 mmol, 5.0 mol%), $Cu(OAc)_2$ (3.2 mg, 0.017 mmol, 5.0 mol%) and dry THF (0.5 mL), followed by addition of DMMS (146 mg, 169 µL, 1.36 mmol, 400 mol%). The mixture was stirred for 10 minutes until the color changed from blue to orange. A second reaction tube equipped with a stirring bar was charged with **VPnone** (50 mg, 0.34 mmol, 100 mol%). The catalyst solution from the first tube was added via syringe to the second tube and the resulting mixture was allowed to stir at room temperature for 15 minutes. The aminating agent **Amine-tBu** (122 mg, 0.41 mmol, 120 mol%) was then added and the tube was transferred to an oil bath heated to 328 K and stirred for 36 hours. The reaction mixture was allowed to cool to room temperature and the solvent was removed under vacuum. A saturated solution of NH_4F in MeOH (5 mL) was added and the mixture was stirred at rt for 10 min, followed by addition of a saturated aqueous solution of Na_2CO_3 (5 mL) and EtOAc (2 mL). The phases were separated and the aqueous layer was extracted with EtOAc (2 x 2 mL). 1,3,5-trimethoxybenzene (57.2 mg, 0.34 mmol, 100 mol%) was added to the combined organic phases and the NMR yield of 79% (±5%) was established after evaporation of the solvents. The ^{1}H NMR spectrum of the crude material and the HPLC trace of **APnol** are shown in **Supplementary Fig. 10**.

Catalysis with (*S*)-DTBM-SEGPHOS, Supplementary Fig. 16:

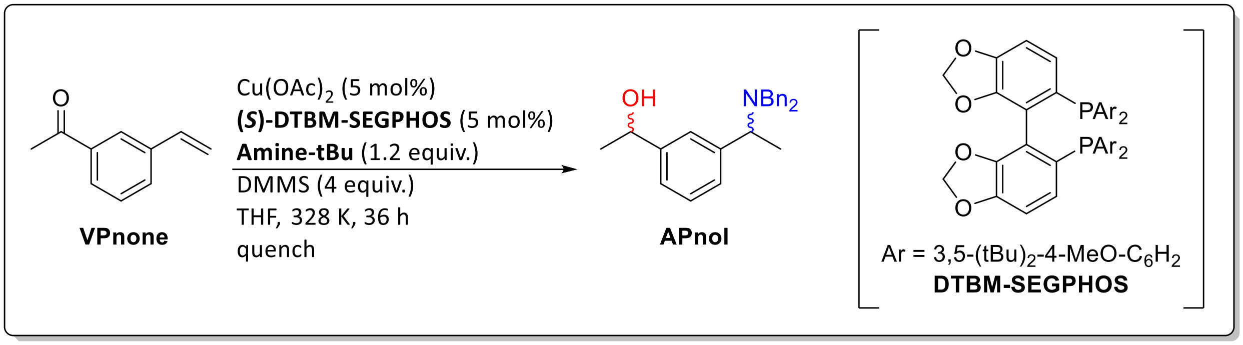

An oven-dried reaction tube was loaded with **(*S*)-DTBM-SEGPHOS** (80.7 mg, 0.068 mmol, 5.0 mol%), $Cu(OAc)_2$ (12.4 mg, 0.068 mmol, 5.0 mol%) and dry THF (1 mL), followed by addition of DMMS (582 mg, 676 µL, 5.48 mmol, 400 mol%). The mixture was stirred for 10 minutes until the color changed from blue to orange. A second reaction tube equipped with a stirring bar was charged with **VPnone** (200 mg, 1.37 mmol, 100 mol%) and **Amine-tBu** (488 mg, 1.64 mmol, 120 mol%). The catalyst solution from the first tube was added via syringe to the second tube and the resulting mixture was allowed to stir at 328 K for 36 hours. The reaction mixture was allowed to cool to room temperature and the solvent was removed under vacuum. A saturated solution of NH_4F in MeOH (10 mL) was added and the mixture was stirred at rt for 10 min, followed by addition of a saturated aqueous solution of Na_2CO_3 (10 mL) and EtOAc (10 mL). The phases were separated and the aqueous layer was extracted with EtOAc (2×10 mL). The combined organic phases were concentrated in vacuum and purified by flash column chromatography over silica gel, eluting with petroleum ether/dichloromethane (gradient from 100/0 to 30/70) to yield **APnol** as a faint yellow oil (236 mg, 50% yield). The ^1H NMR spectrum and the HPLC trace of **APnol** are shown in **Supplementary Fig. 22** and **Supplementary Fig. 16b**, respectively.

The same procedure was followed for the catalysis performed with **(*R*)-DTBM-SEGPHOS** yielding **APnol** in 56% yield. The ^1H NMR spectrum and the HPLC trace of **APnol** are shown in **Supplementary Fig. 21** and **Supplementary Fig. 16a**, respectively.

<u>Standard conditions with BTA helical catalyst, concomitant process, Supplementary Table 1, entry 1:</u>

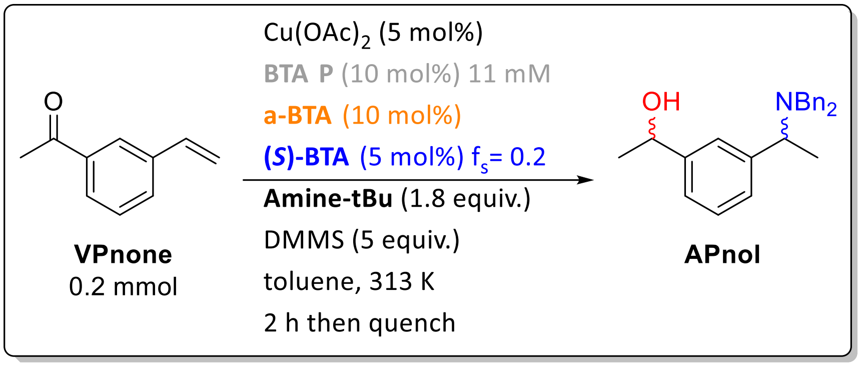

An oven-dried reaction tube was loaded with **BTA P** (21.7 mg, 22.5 µmol, 10.0 mol%), $Cu(OAc)_2$ (2.1 mg, 11.3 µmol, 5.0 mol%) and anhydrous THF (500 µL), and the mixture was stirred for 10 minutes. The solvent was then removed under vacuum and the tube was kept in vacuum (10^{-3} mbar) for 1 hour. **(*S*)-BTA** (11.9 mg, 11.3 µmol, 5 mol%), **a-BTA** (24.6 mg, 22.5 µmol, 10.0 mol%), and anhydrous toluene (2 mL) were added to the tube and the mixture was briefly heated to reflux and stirred for 10 minutes at room temperature. **VPnone** (32.9 mg, 225 µmol, 100 mol%) and **Amine-tBu** (120 mg, 405 µmol, 180 mol%) were added to the tube. The reaction mixture was heated to 313 K. DMMS was added to the tube (140 µL, 1125 µmol, 500 mol%) and the mixture was stirred for 2 hours. The reaction mixture was cooled down to room temperature. A saturated solution of NH_4F (2 mL) in MeOH was added and the mixture was stirred until it became transparent. Then a saturated aqueous solution of Na_2CO_3 (2 mL) as well as EtOAc (1 mL) were added. The phases were separated and the aqueous layer was extracted with EtOAc (2×1 mL). 1,3,5-trimethoxybenzene (37.8 mg, 225 µmol, 100 mol%) was added to the combined organic phases and the NMR yield was established after evaporation of the solvents (66% ±5%). The ^{1}H NMR spectrum and the HPLC trace of the **APnol** crude sample are shown in **Supplementary Fig. 3** and **Supplementary Fig. 25**, respectively.

Catalysis with BTA racemic helices, Supplementary Fig. 11:

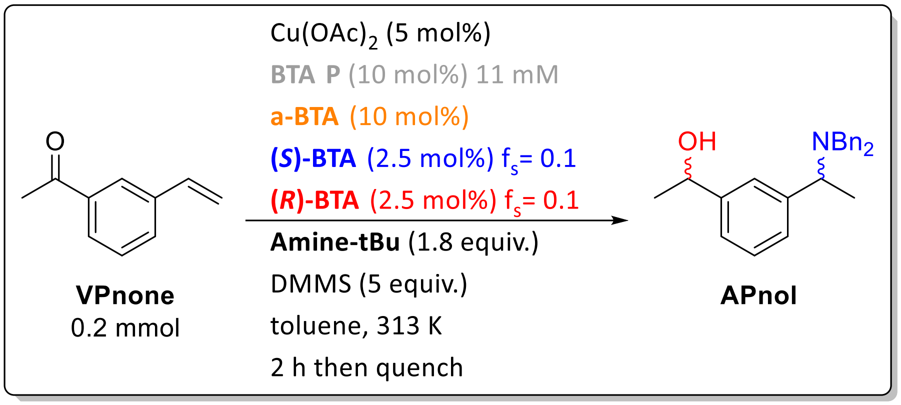

An oven-dried reaction tube was loaded with **BTA P** (21.7 mg, 22.5 µmol, 10.0 mol%), $Cu(OAc)_2$ (2.1 mg, 11.3 µmol, 5.0 mol%) and anhydrous THF (500 µL), and the mixture was stirred for 10 minutes. The solvent was then removed under vacuum and the tube was kept in vacuum (10^{-3} mbar) for 1 hour. **(*S*)-BTA** (5.9 mg, 5.6 µmol, 2.5 mol%), **(*R*)-BTA** (5.9 mg, 5.6 µmol, 2.5 mol%) **a-BTA** (24.6 mg, 22.5 µmol, 10.0 mol%), and anhydrous toluene (2 mL) were added to the tube and the mixture was briefly heated to reflux and stirred for 10 minutes at room temperature. **VPnone** (32.9 mg, 225 µmol, 100 mol%) and **Amine-tBu** (120 mg, 405 µmol, 180 mol%) were added to the tube. The reaction mixture was heated to 313 K. DMMS was added to the tube (140 µL, 1125 µmol, 500 mol%) and the mixture was stirred for 2 hours. The reaction mixture was cooled down to room temperature. A saturated solution of NH_4F (2 mL) in MeOH was added and the mixture was stirred until it became transparent. Then a saturated aqueous solution of Na_2CO_3 (2 mL) as well as EtOAc (1 mL) were added. The phases were separated and the aqueous layer was extracted with EtOAc (2×1 mL). 1,3,5-trimethoxybenzene (37.8 mg, 225 µmol, 100 mol%) was added to the combined organic phases and the NMR yield was established after evaporation of the solvents (71±5%). The HPLC trace of **APnol** is shown in **Supplementary Fig. 11**.

Procedure for monitoring of the concomitant HS/HA of VPnone, Supplementary Fig. 4d-f:

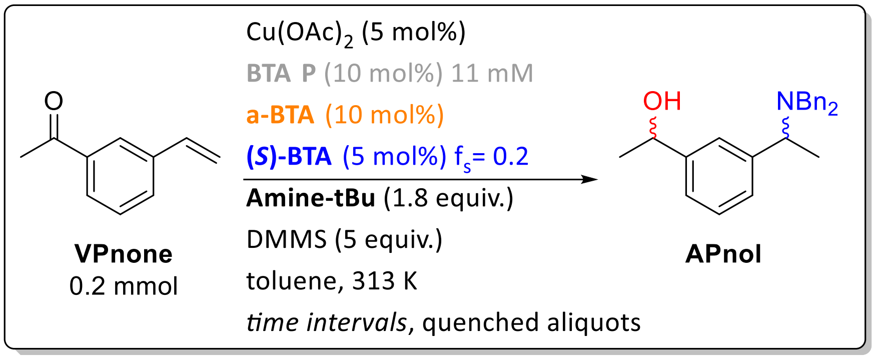

An oven-dried reaction tube was loaded with **BTA P** (21.7 mg, 22.5 μmol, 10.0 mol%), $Cu(OAc)_2$ (2.1 mg, 11.3 μmol, 5.0 mol%) and anhydrous THF (500 μL), and stirred for 10 minutes on a shaking machine. The solvent was then removed under vacuum and the tube was kept in vacuum (10^{-3} mbar) for 1 hour. **(*S*)-BTA** (11.9 mg, 11.3 μmol, 5 mol%), **a-BTA** (24.6 mg, 22.5 μmol, 10.0 mol%), and anhydrous toluene (2 mL) were added to the tube and the mixture was briefly heated to reflux and stirred for 10 minutes at room temperature. **VPnone** (32.9 mg, 225 μmol, 100 mol%), **Amine-tBu** (120 mg, 405 μmol, 180 mol%) and 1,3,5-trimethoxybenzene (37.8 mg, 225 μmol, 100 mol%) were added to the tube. The reaction mixture was stirred and heated to 313 K, followed by addition of DMMS (140 μL, 1125 μmol, 500 mol%, t0). Aliquots (200 μL) of the resulting mixture were collected after 3', 6', 9', 12' and 15', quenched with a saturated solution of NH_4F in MeOH. ^1H NMR spectra were recorded by dissolving in $CDCl_3$ the crude mixtures obtained after evaporation of the solvents.

Screening of secondary phosphine ligands, Supplementary Table 1:

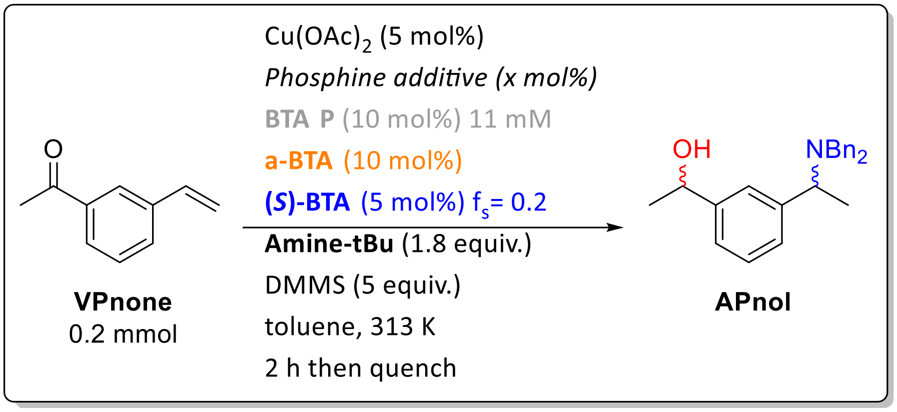

An oven-dried reaction tube was loaded with **BTA P** (21.7 mg, 22.5 µmol, 10.0 mol%), $Cu(OAc)_2$ (2.1 mg, 11.3 µmol, 5.0 mol%), ***phosphine additive (amount, x mol%)*** and anhydrous THF (500 µL), and the mixture was stirred for 10 minutes. The solvent was then removed under vacuum and the tube was kept in vacuum (10^{-3} mbar) for 1 hour. **(*S*)-BTA** (11.9 mg, 11.3 µmol, 5 mol%), **a-BTA** (24.6 mg, 22.5 µmol, 10.0 mol%), and anhydrous toluene (2 mL) were added to the tube and the mixture was briefly heated to reflux and stirred for 10 minutes at room temperature. **VPnone** (32.9 mg, 225 µmol, 100 mol%) and **Amine-tBu** (120 mg, 405 µmol, 180 mol%) were added to the tube. The reaction mixture was heated to 313 K. DMMS was added to the tube (140 µL, 1125 µmol, 500 mol%) and the mixture was stirred for 2 hours. The reaction mixture was cooled down to room temperature. A saturated solution of NH_4F (2 mL) in MeOH was added and the mixture was stirred until it became transparent. Then a saturated aqueous solution of Na_2CO_3 (2 mL) as well as EtOAc (1 mL) were added. The phases were separated and the aqueous layer was extracted with EtOAc (2×1 mL). 1,3,5-trimethoxybenzene (37.8 mg, 225 µmol, 100 mol%) was added to the combined organic phases and the NMR yield was established after evaporation of the solvents.

<u>Isolated yields with helical BTA catalyst, concomitant process, Supplementary Table 3:</u>

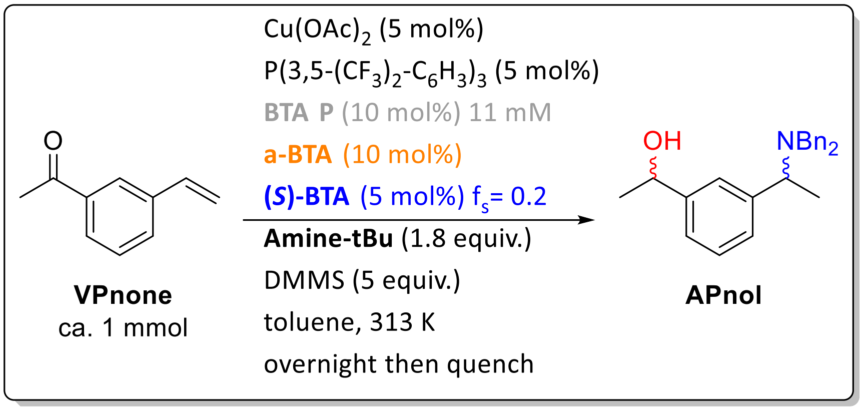

An oven-dried 50 mL Schlenk tube was loaded with **BTA P** (108.5 mg, 0.113 mmol, 10.0 mol%), $Cu(OAc)_2$ (10.3 mg, 0.056 mmol, 5.0 mol%), P(3,5-$(CF_3)_2$-$C_6H_3)_3$ (38 mg, 0.056 mmol, 5 mol%) and anhydrous THF (2.5 mL), and the mixture was stirred for 10 minutes. The solvent was then removed under vacuum and the tube was kept in vacuum (10^{-3} mbar) for 1 hour. **(*S*)-BTA** (59.5 mg, 0.056 mmol, 5 mol%), **a-BTA** (123 mg, 0.113 mmol, 10.0 mol%) and anhydrous toluene (10 mL) were added to the tube and the mixture was briefly heated to reflux and stirred for 10 minutes at room temperature. **VPnone** (164.5 mg, 1.13 mmol, 100 mol%) and **Amine-tBu** (600 mg, 2.02 mmol, 180 mol%) were added to the tube. The Schlenk tube was sealed with a rubber septum, then evacuated and backfilled with N_2 three times. An atmosphere of nitrogen was maintained during the following addition. The reaction mixture was stirred and heated to 313 K, prior to the addition of DMMS (598 mg, 695 µL, 5.63 mmol, 500 mol%) via syringe. After overnight, the reaction mixture was cooled down to room temperature. A saturated solution of NH_4F (10 mL) in MeOH was added and the mixture was stirred until it became transparent. Then a saturated aqueous solution of Na_2CO_3 (10 mL) as well as EtOAc (5 mL) were added. The phases were separated and the aqueous layer was extracted with EtOAc (2×5 mL). The organic phases were collected and evaporated under vacuum. The crude product was then purified by flash column chromatography, eluting with petroleum ether/dichloromethane (gradient from 100/0 to 30/70), to yield **APnol** as a faint yellow oil (212 mg, 51% yield). The ^{1}H NMR spectrum and the HPLC trace of **APnol** are shown **Supplementary Fig. 41** and **Supplementary Fig. 29**, respectively (also **Fig. 3** for the HPLC trace).

Catalysis with **(*R*)-BTA** instead of **(*S*)-BTA** was conducted following the same procedure, yielding **APnol** in 48% yield. The ^{1}H NMR spectrum and the HPLC trace of **APnol** are shown in **Supplementary Fig. 41** and **Supplementary Fig. 30**, respectively (also **Fig. 3** for the HPLC trace).

__Procedure for monitoring of the HS of VPnone, Supplementary Figs. 4-5:__

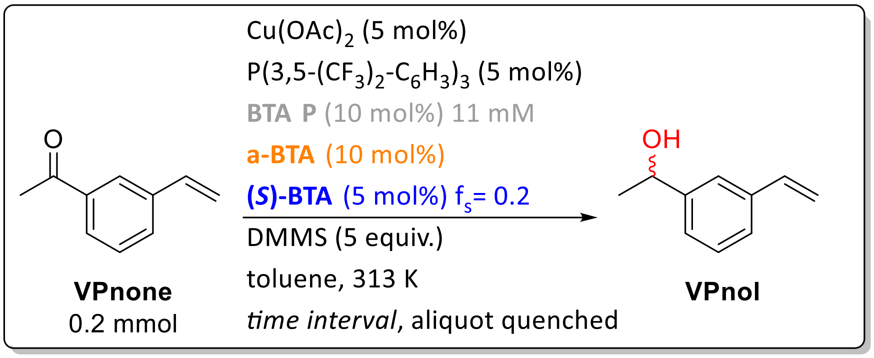

Under air (**Supplementary Fig. 4**): An oven-dried reaction tube was loaded with **BTA P** (21.7 mg, 22.5 µmol, 10.0 mol%), $Cu(OAc)_2$ (2.1 mg, 11.3 µmol, 5.0 mol%), $P(3,5\text{-}(CF_3)_2\text{-}C_6H_3)_3$ (7.6 mg, 11.3 µmol, 5 mol%) and anhydrous THF (500 µL), and stirred for 10 minutes on a shaking machine. The solvent was then removed under vacuum and the tube was kept in vacuum (10^{-3} mbar) for 1 hour. **(*S*)-BTA** (11.9 mg, 11.3 µmol, 5 mol%), **a-BTA** (24.6 mg, 22.5 µmol, 10.0 mol%) and anhydrous toluene (2 mL) were added to the tube and the mixture was briefly heated to reflux and stirred for 10 minutes at room temperature. **VPnone** (32.9 mg, 225 µmol, 100 mol%) and 1,3,5-trimethoxybenzene (37.8 mg, 225 µmol, 100 mol%) were added to the tube. The reaction mixture was stirred and heated to 313 K, followed by the addition of DMMS (140 µL, 1125 µmol, 500 mol%, t0). Aliquots (200 µL) of the resulting mixture were collected after 3’, 6’, 9’, 12’ and 15’, quenched with a saturated solution of NH_4F in MeOH. ^{1}H NMR spectra were recorded by dissolving in $CDCl_3$ the crude mixtures obtained after evaporation of the solvents.

Under nitrogen (**Supplementary Fig. 5**): An oven-dried 25 mL Schlenk tube was loaded with **BTA P** (21.7 mg, 22.5 µmol, 10.0 mol%), $Cu(OAc)_2$ (2.1 mg, 11.3 µmol, 5.0 mol%),), $P(3,5\text{-}(CF_3)_2\text{-}C_6H_3)_3$ (7.6 mg, 11.3 µmol, 5 mol%) and anhydrous THF (2.5 mL), and stirred for 10 minutes on a shaking machine. The solvent was then removed under vacuum and the tube was kept in vacuum (10^{-3} mbar) for 1 hour. **(*S*)-BTA** (11.9 mg, 11.3 µmol, 5 mol%), **a-BTA** (12.3 mg, 11.3 µmol, 5.0 mol%) and anhydrous toluene (2 mL) were added to the tube and the mixture was briefly heated to reflux and stirred for 10 minutes at room temperature. The reaction mixture was then transferred into a desiccator containing P_2O_5 and dried for 1 day. The tube was the removed from the desiccator, briefly heated to reflux and stirred for 10 minutes at room temperature. **VPnone** (32.9 mg, 225 µmol, 100 mol%) and 1,3,5-trimethoxybenzene (37.8 mg, 225 µmol, 100 mol%) were added to the tube. The Schlenk tube was sealed with a rubber septum, then evacuated and backfilled with nitrogen three times. An atmosphere of nitrogen was maintained during the following additions. The reaction mixture was stirred and heated to 313 K and DMMS (140 µL, 1125 µmol, 500 mol%, t0) was added. Aliquots (200 µL) of the resulting mixture were collected after 15’, 30’, 60’ and 90’, quenched with a saturated solution of NH_4F in MeOH. ^{1}H NMR spectra were recorded by dissolving in $CDCl_3$ the crude mixtures obtained after evaporation of the solvents.

Procedure for the sequential reaction without switching of the handedness of the BTA helical catalyst, under air, Supplementary Fig. 7:

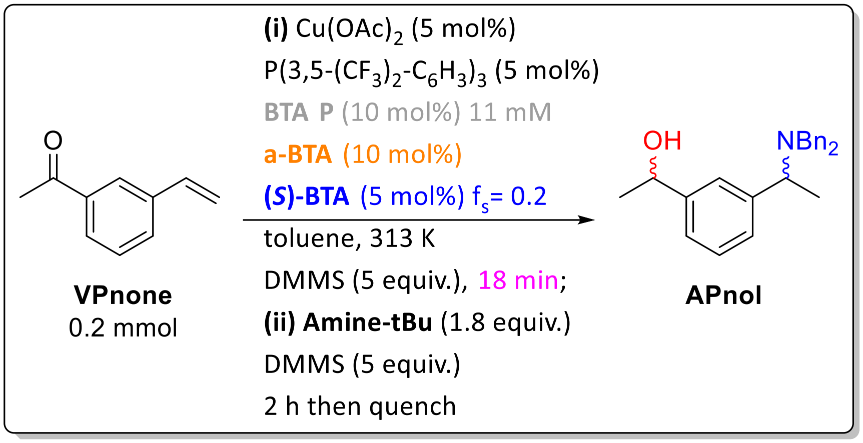

An oven-dried reaction tube was loaded with **BTA P** (21.7 mg, 22.5 μmol, 10.0 mol%), $Cu(OAc)_2$ (2.1 mg, 11.3 μmol, 5.0 mol%), $P(3,5-(CF_3)_2-C_6H_3)_3$ (7.6 mg, 11.3 μmol, 5.0 mol%) and anhydrous THF (500 μL), and stirred for 10 minutes on a shaking machine. The solvent was then removed under vacuum and the tube was kept in vacuum (10^{-3} mbar) for 1 hour. **(*S*)-BTA** (11.9 mg, 11.3 μmol, 5 mol%), **a-BTA** (24.6 mg, 22.5 μmol, 10.0 mol%) and anhydrous toluene (2 mL) were added to the tube and the mixture was briefly heated to reflux and stirred for 10 minutes at room temperature. **VPnone** (32.9 mg, 225 μmol, 100 mol%) was added to the tube. The reaction mixture was heated to 313 K and DMMS (140 μL, 1125 μmol, 500 mol%) was added. The mixture was stirred for 18 minutes at 313 K. Then **Amine-tBu** (120 mg, 405 μmol, 180 mol%) as well as DMMS (140 μL, 1125 μmol, 500 mol%) were added successively. After 2 hours, the reaction mixture was cooled down to room temperature. A saturated solution of NH_4F (2 mL) in MeOH was added to quench the mixture and the mixture was stirred until it became transparent. Then a saturated aqueous solution of Na_2CO_3 (2 mL) as well as EtOAc (1 mL) were added. The phases were separated and the aqueous layer was extracted with EtOAc (2×1 mL). 1,3,5-trimethoxybenzene (37.8 mg, 225 μmol, 100 mol%) was added to the combined organic phases and the NMR yield was established after evaporation of the solvents. The ^1H NMR spectrum and the HPLC trace of **APnol** are shown in **Supplementary Fig. 7** and **Supplementary Fig. 31**, respectively.

Procedure for the sequential reaction with switching of the handedness of the BTA helical catalyst, under air, Table 2, entry 1:

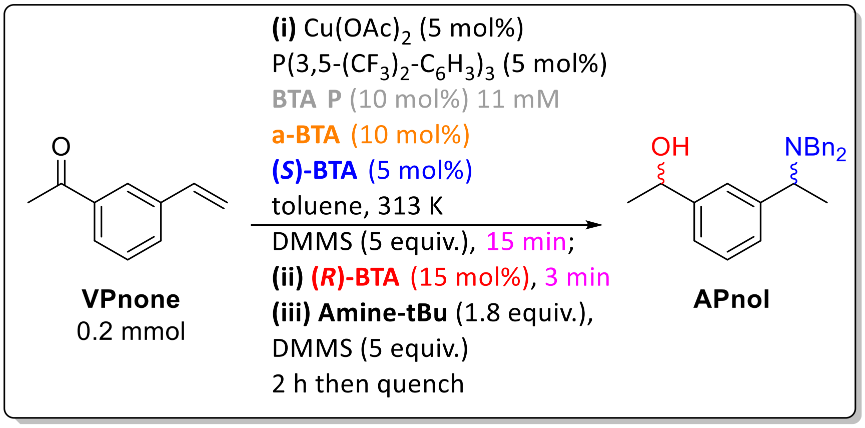

An oven-dried reaction tube was loaded with **BTA P** (21.7 mg, 22.5 µmol, 10.0 mol%), $Cu(OAc)_2$ (2.1 mg, 11.3 µmol, 5.0 mol%), $P(3,5\text{-}(CF_3)_2\text{-}C_6H_3)_3$ (7.6 mg, 11.3 µmol, 5 mol%) and anhydrous THF (500 µL), and stirred for 10 minutes on a shaking machine. The solvent was then removed under vacuum and the tube was kept in vacuum (10^{-3} mbar) for 1 hour. **(*S*)-BTA** (11.9 mg, 11.3 µmol, 5 mol%), **a-BTA** (24.6 mg, 22.5 µmol, 10.0 mol%) and anhydrous toluene (2 mL) were added to the tube and the mixture was briefly heated to reflux and stirred for 10 minutes at room temperature. **VPnone** (32.9 mg, 225 µmol, 100 mol%) was added to the tube. The reaction mixture was heated to 313 K and DMMS (140 µL, 1125 µmol, 500 mol%) was added. The reaction mixture was stirred for 15 minutes at 313 K. A solution of **(*R*)-BTA** (35.7 mg, 33.9 µmol, 15 mol%) in toluene (0.2 mL) was then added and the mixture was allowed to stir for additional 3 minutes at 313 K. Then **Amine-tBu** (120 mg, 405 µmol, 180 mol%) as well as DMMS (140 µL, 1125 µmol, 500 mol%) were added successively. After 2 hours, the reaction mixture was cooled down to room temperature. A saturated solution of NH_4F (2 mL) in MeOH was added to quench the mixture and the mixture was stirred until it became transparent. Then a saturated aqueous solution of Na_2CO_3 (2 mL) as well as EtOAc (1 mL) were added. The phases were separated and the aqueous layer was extracted with EtOAc (2×1 mL). 1,3,5-trimethoxybenzene (37.8 mg, 225 µmol, 100 mol%) was added to the combined organic phases and the NMR yield was established after evaporation of the solvents. The mixture was then filtered over silica plug and eluted with dichloromethane prior to HPLC analysis. The HPLC trace of **APnol** is shown in **Supplementary Fig. 33**.

Catalysis starting with **(*R*)-BTA** and with addition of **(*S*)-BTA** after the HS step (**Table 2**, entry 2) was conducted following the same procedure. The HPLC trace of **APnol** is shown in **Supplementary Fig. 34.**

Procedure for the sequential reaction with switching of the handedness of the BTA helical catalyst, optimized conditions under air, Table 2, entry 3:

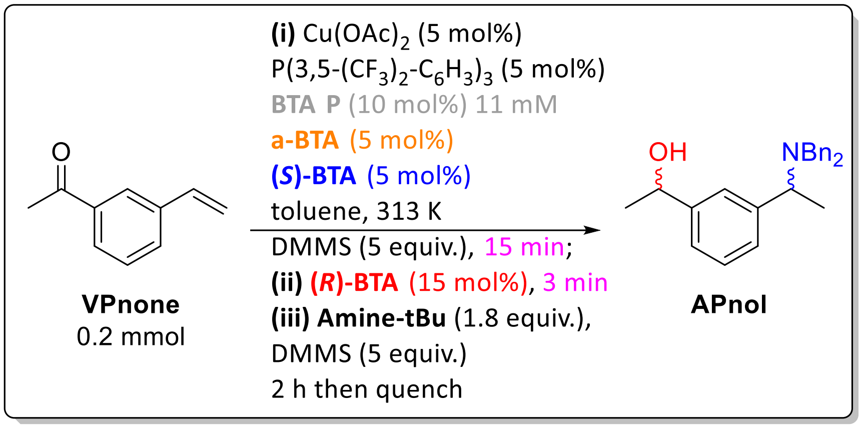

An oven-dried reaction tube was loaded with **BTA P** (21.7 mg, 22.5 µmol, 10.0 mol%), $Cu(OAc)_2$ (2.1 mg, 11.3 µmol, 5.0 mol%), $P(3,5-(CF_3)_2-C_6H_3)_3$ (7.6 mg, 11.3 µmol, 5 mol%) and anhydrous THF (500 µL), was stirred for 10 minutes on a shaking machine. The solvent was then removed under vacuum and the tube was kept in vacuum (10^{-3} mbar) for 1 hour. **(*S*)-BTA** (11.9 mg, 11.3 µmol, 5 mol%), **a-BTA** (12.3 mg, 11.25 µmol, 5.0 mol%) and anhydrous toluene (2 mL) were added to the tube and the mixture was briefly heated to reflux and stirred for 10 minutes at room temperature. **VPnone** (32.9 mg, 225 µmol, 100 mol%) was added to the tube. The reaction mixture was heated to 313 K and DMMS (140 µL, 1125 µmol, 500 mol%) was added. The reaction mixture was stirred for 15 minutes at 313 K. A solution of **(*R*)-BTA** (35.7 mg, 33.9 µmol, 15 mol%) in toluene (0.2 mL) was then added and the mixture was allowed to stir for additional 3 minutes at 313 K. Then **Amine-tBu** (120 mg, 405 µmol, 180 mol%) as well as DMMS (140 µL, 1125 µmol, 500 mol%) were added successively. After 2 hours, the reaction mixture was cooled down to room temperature. A saturated solution of NH_4F (2 mL) in MeOH was added to quench the mixture and the mixture was stirred until it became transparent. Then a saturated aqueous solution of Na_2CO_3 (2 mL) as well as EtOAc (1 mL) were added. The phases were separated and the aqueous layer was extracted with EtOAc (2×1 mL). 1,3,5-trimethoxybenzene (37.8 mg, 225 µmol, 100 mol%) was added to the combined organic phases and the NMR yield was established after evaporation of the solvents. The mixture was then filtered over silica plug and eluted with dichloromethane prior to HPLC analysis. The ^{1}H NMR spectrum and the HPLC trace of **APnol** are shown in **Supplementary Fig. 8** and **Supplementary Fig. 35** (also **Fig. 3**), respectively.

Catalysis starting with **(*R*)-BTA** and with addition of **(*S*)-BTA** after the HS step (Table 2, entry 4) was conducted following the same procedure. The ^{1}H NMR spectrum and the HPLC trace of **APnol** are shown in **Supplementary Fig. 8** and **Supplementary Fig. 36** (also **Fig. 3**), respectively.

Procedure for the sequential reaction with switching of the handedness of the BTA helical catalyst, under N_2 and anhydrous conditions, Table 2, entry 5:

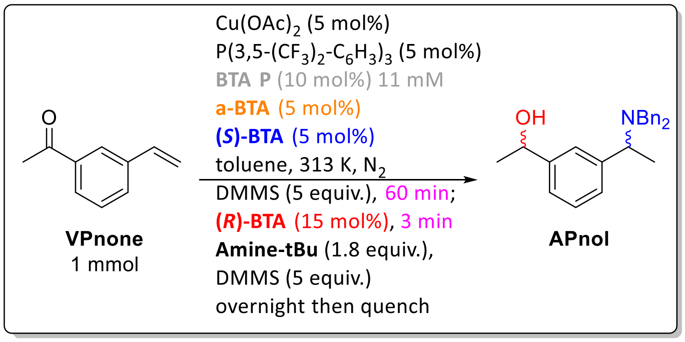

An oven-dried 25 mL Schlenk tube was loaded with **BTA P** (108.5 mg, 0.113 mmol, 10.0 mol%), $Cu(OAc)_2$ (10.3 mg, 0.056 mmol, 5.0 mol%), P(3,5-$(CF_3)_2$-$C_6H_3)_3$ (38 mg, 0.056 mmol, 5 mol%) and anhydrous THF (2.5 mL), and stirred for 10 minutes on a shaking machine. The solvent was then removed under vacuum and the tube was kept in vacuum (10^{-3} mbar) for 1 hour. **(*S*)-BTA** (59.5 mg, 0.056 mmol, 5 mol%), **a-BTA** (61.5 mg, 0.056 mmol, 5.0 mol%) and anhydrous toluene (10 mL) were added to the tube and the mixture was briefly heated to reflux and stirred for 10 minutes at room temperature. The reaction mixture was then transferred into a desiccator containing P_2O_5 and dried for 1 day. The tube was the removed from the desiccator, briefly heated to reflux and stirred for 10 minutes at room temperature. **VPnone** (164.5 mg, 1.13 mmol, 100 mol%) was added to the tube. The Schlenk tube was sealed with a rubber septum, then evacuated and backfilled with nitrogen three times. An atmosphere of nitrogen was maintained during the following additions. The reaction mixture was heated to 313 K and DMMS (598 mg, 695 µL, 5.63 mmol, 500 mol%) was added. The reaction was stirred for 60 minutes at 313 K. A solution of **(*R*)-BTA** (178.5 mg, 169.5 µmol, 15 mol%) in toluene (0.2 mL) was then added and the mixture was allowed to stir for additional 3 minutes. Then **Amine-tBu** (600 mg, 2.02 mmol, 180 mol%) as well as DMMS (598 mg, 695 µL, 5.63 mmol, 500 mol%) were added successively and the mixture was stirred overnight. The reaction mixture was then cooled down to room temperature. A saturated solution of NH_4F (10 mL) in MeOH was added to quench the mixture, and the mixture was stirred until it became transparent. Then a saturated aqueous solution of Na_2CO_3 (10 mL) as well as EtOAc (5 mL) were added. The phases were separated and the aqueous layer was extracted with EtOAc (2×5 mL). 1,3,5-trimethoxybenzene (189 mg, 1.13 mmol, 100 mol%) was added to the combined organic phases and the NMR yield was established after evaporation of the solvents. The crude product was then purified by flash column chromatography, eluting with petroleum ether/dichloromethane (gradient from 100/0 to 30/70), to yield **APnol** as a faint yellow oil (151 mg, 38% yield). The ^{1}H NMR spectrum of the crude sample, the ^{1}H NMR spectrum of the purified **APnol** and the HPLC trace of purified **APnol** are shown in **Supplementary Fig. 9**, **Supplementary Fig. 23** and **Supplementary Fig. 37**, respectively.

Catalysis starting with **(*R*)-BTA** and with addition of **(*S*)-BTA** after the HS step (**Table 2**, entry 6) was conducted following the same procedure. The ^{1}H NMR spectrum of the crude sample, the ^{1}H NMR spectrum of the purified sample and the HPLC trace of purified **APnol** are shown in in **Supplementary Fig. 9**, **Supplementary Fig. 24** and **Supplementary Fig. 38**, respectively.

Preparation of the solution for CD analyses of the BTA pre-catalyst (CD data for Fig. 2)

<u>CD analysis before stereochemical switch</u>: an oven-dried reaction tube was loaded with **BTA P** (21.7 mg, 22.5 μmol), $Cu(OAc)_2$ (2.1 mg, 11.3 μmol), $P(3,5\text{-}(CF_3)_2\text{-}C_6H_3)_3$ (7.6 mg, 11.3 μmol, 5 mol%) and anhydrous THF (500 μL), and stirred for 10 minutes. The solvent was then removed under vacuum and the tube was kept in vacuum (10^{-3} mbar) for 1 hour. **(*S*)-BTA** (11.9 mg, 11.3 μmol), **a-BTA** (24.6 mg, 22.5 μmol), and anhydrous toluene (2 mL) were added to the tube and the mixture was briefly heated to reflux and stirred for 10 minutes at room temperature. The tube was then transferred to oil bath heated to 313 K. An aliquot of the resulting mixture was collected and added to a spectrosil quartz cell of 0.05 mm pathlength, and the CD spectrum was recorded at 313 K.

<u>CD analysis after stereochemical switch</u>: **(*R*)-BTA** (35.7 mg, 33.9 μmol) in anhydrous toluene (0.2 mL) was then added to the aforementioned reaction tube at 313 K. The mixture was allowed to stir for 2 minutes. An aliquot was collected and added to a spectrosil quartz cell of 0.05 mm pathlength, and the CD spectrum was recorded at 313 K. The end of the CD analysis corresponds to *ca.* 3 minutes after the addition of **(*R*)-BTA**.

Supplementary references

[1] D. Niu, S. L. Buchwald, *J. Am. Chem. Soc.* **2015**, *137*, 9716–9721.
[2] B. H. Lipshutz, K. Noson, W. Chrisman, A. Lower, *J. Am. Chem. Soc.* **2003**, *125*, 8779–8789.
[3] P. Aoun, A. Hammoud, M. A. Martínez-Aguirre, L. Bouteiller, M. Raynal, *Catal. Sci. Technol.* **2022**, *12*, 834–842.
[4] H. Kong, Li, Yan, Hammoud, Ahmad, Dubreucq, Ludovic, Troufflard, Claire, Maruchenko, Régina, Bouteiller, Laurent, Raynal, Matthieu, *ChemistryEurope* **2023**, *1*, e202300027.
[5] S. Sirol, J. Courmarcel, N. Mostefai, O. Riant, *Org. Lett.* **2001**, *3*, 4111–4113.
[6] J. Courmarcel, N. Mostefaï, S. Sirol, S. Choppin, O. Riant, *Isr. J. Chem.* **2001**, *41*, 231–240.
[7] J. Wu, J.-X. Ji, A. S. C. Chan, *Proc. Natl. Acad. Sci.* **2005**, *102*, 3570–3575.
[8] N. Mostefaï, S. Sirol, J. Courmarcel, O. Riant, *Synthesis* **2007**, 1265–1271.
[9] X.-C. Zhang, Y. Wu, F. Yu, F.-F. Wu, J. Wu, A. S. C. Chan, *Chem. – Eur. J.* **2009**, *15*, 5888–5891.
[10] C. Deutsch, N. Krause, *Chem. Rev.* **2008**, *108*, 2916–2927.
[11] M. T. Pirnot, Y.-M. Wang, S. L. Buchwald, *Angew. Chem. Int. Ed.* **2016**, *55*, 48–57.
[12] R. Y. Liu, S. L. Buchwald, *Acc. Chem. Res.* **2020**, *53*, 1229–1243.
[13] S.-L. Shi, Z. L. Wong, S. L. Buchwald, *Nature* **2016**, *532*, 353–356.
[14] A. Saxena, B. Choi, H. W. Lam, *J. Am. Chem. Soc.* **2012**, *134*, 8428–8431.
[15] S. Ichikawa, S. L. Buchwald, *Org. Lett.* **2019**, *21*, 8736–8739.
[16] S. Zhu, N. Niljianskul, S. L. Buchwald, *J. Am. Chem. Soc.* **2013**, *135*, 15746–15749.
[17] S. Nosé, *Mol. Phys.* **1984**, *52*, 255–268.
[18] L. Verlet, *Phys. Rev.* **1967**, *159*, 98–103.
[19] N. S. Shahana Nizar, M. Sujith, K. Swathi, C. Sissa, A. Painelli, K. George Thomas, *Chem. Soc. Rev.* **2021**, *50*, 11208–11226.
[20] J. M. Paolillo, A. D. Duke, E. S. Gogarnoiu, D. E. Wise, M. Parasram, *J. Am. Chem. Soc.* **2023**, *145*, 2794–2799.
[21] V. A. Steadman, S. B. Pettit, K. G. Poullennec, L. Lazarides, A. J. Keats, D. K. Dean, S. J. Stanway, C. A. Austin, J. A. Sanvoisin, G. M. Watt, H. G. Fliri, A. C. Liclican, D. Jin, M. H. Wong, S. A. Leavitt, Y.-J. Lee, Y. Tian, C. R. Frey, T. C. Appleby, U. Schmitz, P. Jansa, R. L. Mackman, B. E. Schultz, *J. Med. Chem.* **2017**, *60*, 1000–1017.
[22] G. A. Molander, A. R. Brown, *J. Org. Chem.* **2006**, *71*, 9681–9686.
